# Safety of first-line systemic therapy in patients with metastatic colorectal cancer: a network meta-analysis of randomized controlled trials

**DOI:** 10.1186/s12885-024-12662-3

**Published:** 2024-07-24

**Authors:** Yanrong Zhan, Xianwen Cheng, Pingping Mei, Shufa Tan, Wenzhe Feng, Hua Jiang

**Affiliations:** 1 Rudong People’s Hospital / Affiliated Rudong Hospital of Xinglin College, Nantong University, Nantong, Jiangsu 226400 China; 2 Ankang Hospital of Traditional Chinese Medicine, Ankang, Shaanxi 725000 China; 3 Shanghai University of Traditional Chinese Medicine, Shanghai, 201203 China; 4 Shaanxi University of Chinese Medicine, Xianyang, Shaanxi 712000 China; 5 Affiliated Hospital of Shaanxi University of Chinese Medicine, Xianyang, Shaanxi 712000 China

**Keywords:** Metastatic colorectal cancer, First-line, Systemic therapy, Safety, Adverse events, Toxicity, Network meta-analysis

## Abstract

**Objective:**

To evaluate the safety of first-line systemic therapy for metastatic colorectal cancer through network meta-analysis.

**Methods:**

The literature from PubMed, Embase, Web of Science, and Cochrane Library databases was searched from the inception of the databases to August 15, 2023, and strict inclusion and exclusion criteria were applied to screen studies. The Cochrane Bias Risk Assessment Tool (RoB 2.0) was used to evaluate the quality of the included literature. Network meta-analysis was conducted using Stata 15.0 and R4.3.1 software to compare the incidence of adverse events (AEs) among different treatment regimens.

**Results:**

A total of 53 randomized controlled trials, involving 17,351 patients with metastatic colorectal cancer (mCRC), were ultimately included, encompassing 29 different therapeutic approaches. According to SUCRA rankings, the CAPOX regimen is most likely to rank first in terms of safety, while the FOLFOXIRI + panitumumab regimen is most likely to rank last. In terms of specific AEs, the CAPOX regimen, whether used alone or in combination with targeted drugs (bevacizumab and cetuximab), is associated with a reduced risk of neutropenia and febrile neutropenia, as well as an increased risk of thrombocytopenia and diarrhea. The FOLFOX regimen, with or without bevacizumab, is linked to an increased risk of neutropenia and peripheral sensory neuropathy. The FOLFIRI/CAPIRI + bevacizumab regimen is associated with a reduced risk of peripheral sensory neuropathy. S-1 and S-1 + oxaliplatin are well-tolerated in terms of gastrointestinal reactions. The FOLFOXIRI regimen, whether used alone or in combination with targeted drugs, is associated with various AEs.

**Conclusion:**

In summary, the CAPOX regimen may be the safest option among the first-line systemic treatment regimens for mCRC patients, while the FOLFOXIRI + panitumumab regimen may be associated with a higher incidence of grade 3 or higher AEs.

**Supplementary Information:**

The online version contains supplementary material available at 10.1186/s12885-024-12662-3.

## Introduction

Colorectal cancer (CRC) ranks as the fourth most common cause of cancer-related deaths worldwide, resulting in nearly 900,000 fatalities annually [1]. Metastasis is observed in approximately 20–30% of patients upon colorectal cancer diagnosis [2], and about 10–25% of patients develop metachronous metastasis following treatment-oriented surgery [3]. Metastatic colorectal cancer (mCRC) carries a grim prognosis, with a 5-year survival rate of less than 20% [4]. While surgery and local ablation can address some isolated metastases, there is no cure for the majority of CRC patients with multiple systemic metastases, necessitating palliative systemic treatment to extend overall survival. Presently, the National Comprehensive Cancer Network (NCCN) recommends various first-line systemic treatment regimens for mCRC patients [5]. These regimens primarily consist of dual and triple chemotherapy protocols based on 5-fluorouracil (5-FU) or capecitabine in combination with other cytotoxic agents (oxaliplatin or irinotecan), often with the addition of targeted drugs. However, many of these treatments lack direct comparative clinical trials, and some remain controversial in terms of safety. One meta-analysis indicates that FOLFOXIRI (5-fluorouracil plus leucovorin plus oxaliplatin plus irinotecan) + bevacizumab may increase the incidence of neutropenia and diarrhea compared to dual chemotherapy regimens with or without targeted drugs [6], while two other meta-analyses suggest that this regimen does not elevate treatment toxicity [7, 8]. The presence of toxic side effects restricts the clinical application of chemotherapy regimens and targeted drugs, such as hypertension and proteinuria induced by bevacizumab [9, 10], skin toxicity induced by Panitumumab and Cetuximab [11, 12], and hypomagnesemia related to panitumumab [13]. The safety of treatment protocols significantly impacts patients’ prognosis and quality of life. Reducing drug toxicity, mitigating the risk of adverse reactions, and enhancing patients’ quality of life necessitate ongoing exploration and practice by medical professionals.

Network meta-analysis (NMA) is frequently employed to compare differences among multiple treatment regimens, combining direct and indirect comparisons to offer crucial references for clinical drug usage guidance [14]. In prior NMA studies, more emphasis has been placed on evaluating the effectiveness of systemic therapy for colorectal cancer, with less attention directed toward assessing safety. One meta-analysis assessed the safety of 16 first-line systemic treatment regimens for mCRC patients but was limited to an overall analysis of grade 3 or higher adverse events (AEs) and did not specifically analyze the characteristics of individual AEs [8]. If clinicians have early knowledge of potential AEs and corresponding treatment measures associated with each treatment plan, they can choose the most suitable treatment method based on the specific conditions of patients. This approach is beneficial in enhancing the quality of life for patients. Therefore, by utilizing a network meta-analysis method to evaluate the safety of first-line systemic treatment for metastatic colorectal cancer, this study compares and ranks various adverse reactions to provide guidance for clinical decision-making.

## Materials and methods

### Design and registration

This systematic review study process followed the Preferred Reporting Items for Systematic Reviews and Meta-Analyses (PRISMA 2020) statement [15], and the protocol was registered with the International Prospective Registry of Systematic Reviews (**PROSPERO: CRD42023462360**).

### Literature search

Two researchers (YR.Z, PP.M) independently searched for studies published in PubMed, Embase, Web of Science, and the Cochrane Library from the establishment of the database to August 15, 2023. The search terms were designed by combining subject words and free words. The search keywords encompassed colorectal neoplasm, colorectal cancer, metastasis, first-line chemotherapy, FOLFOX (5-fluorouracil plus leucovorin plus oxaliplatin), FOLFIRI (5-fluorouracil plus leucovorin plus irinotecan), CAPOX (capecitabine plus oxaliplatin), FOLFOXIRI, 5-fluorouracil, capecitabine, bevacizumab, cetuximab, panitumumab, and randomization. Specific search strategies are presented in supplementary materials (Appendix 1).

### Inclusion and exclusion criteria

The inclusion criteria were as follows:

- Population: Patients with pathologically confirmed colorectal cancer and distant metastases who have not received any treatment after diagnosis;
- Interventions: Systemic therapy as first-line treatment for patients with mCRC;
- Outcomes: Grade ≥ 3 AEs or toxic reactions (according to the Common Toxicity Criteria of the American National Cancer Institute) ;
- Study type: Randomized controlled trial (RCT) with sample size ≥ 30;
- Language: English.

Exclusion criteria:

- Participants: Colorectal cancer patients without metastasis;
- Interventions: Non-first line systemic treatment regimens (maintenance therapy, radiation therapy, second- or third-line therapy, etc.) ;
- Outcomes: No relevant outcome indicators or data could not be extracted;
- Non-RCT trials;
- Duplicate studies or secondary analyses, in vitro experiments, animal experiments, reviews, letters, guidelines, case reports, pathological mechanisms, conference abstracts, reviews, or systematic reviews.

### Literature screening and data extraction

Two researchers (YR.Z, PP.M) independently screened all articles identified by the database search. First, EndNote X 9.0 software was used to remove duplicate literature and exclude case reports, conference abstracts, letters, and review articles. Then, the titles and abstracts of the remaining literature were screened to exclude studies that were not relevant to the topic, and the full texts and supplementary information of the remaining studies were reviewed to identify eligible studies. Finally, data were extracted according to a unified extraction table, which included information such as the author, publication year, study design, study country, study period, number of study sites, follow-up time, total number of samples, and the number of AEs. The two researchers (YR.Z, PP.M) independently cross-checked all included papers and extracted data. Any disputed articles were referred to a third researcher (HMF) for consensus resolution.

### Quality assessment

Two independent reviewers (YR.Z and PP.M) assessed the risk of bias using Version 2 of the Cochrane Risk-of-Bias Tool for randomized trials (RoB 2.0) (10.1136/bmj.l4898). Each study was categorized as “low risk of bias,” “some concerns,” or “high risk of bias” across the following domains: bias arising from the randomization process; bias due to deviations from the intended intervention; bias from missing outcome data; bias in the measurement of outcomes; and bias in the selection of the reported results, including deviations from the registered protocol. We rated trials as having an overall high risk of bias if one or more domains were rated “high risk of bias” and as having an overall low risk of bias if all domains were rated “low risk of bias.”

### Statistical analysis

Bayesian random-effects (BRE) models were employed to analyze the effects of interventions and compare their safety. Risk ratio (RR) and its 95% confidence interval (CrI) were used to combine the incidence of AEs, with a 95% CrI excluding 1 representing a statistically significant difference. The Markov chain Monte Carlo method was adopted for modeling, with four Markov chains running simultaneously. The annealing times were set to 20,000, and the modeling was completed after 50,000 simulation iterations [16]. The deviance information criterion (DIC) was used to compare model fit and global consistency. With there was a closed loop in the network, local consistency was analyzed using the node-splitting method [17]. Additionally, interventions were ranked based on the surface under the cumulative rank curve (SUCRA), and league tables were generated to compare differences in effects among interventions [18]. A funnel plot was used to intuitively reflect the heterogeneity among the studies. Analyses were conducted using Stata 15.0 (Stata Corporation, College Station, TX) and R 4.3.1 (R Development Core Team, Vienna, http://www.R-project.org). A difference with a p-value of less than 0.05 was considered statistically significant.

## Results

### Literature search results

**Fig. 1:**
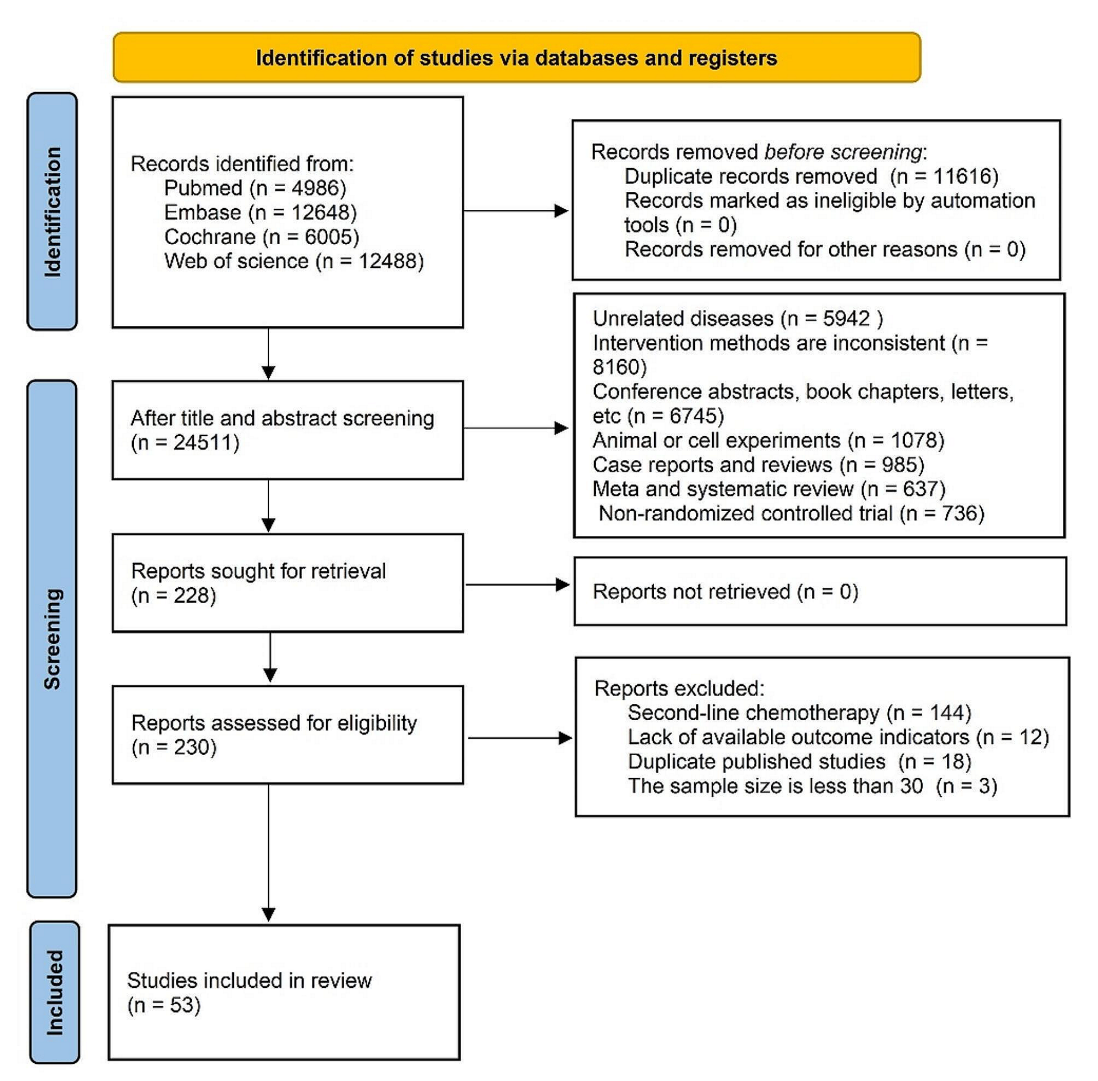
PRISMA flowchart

In the initial literature search, 36,127 articles were retrieved, and 11,616 duplicate studies were excluded. Following the inclusion and exclusion criteria, the article titles and abstracts were carefully examined, leading to the exclusion of 24,283 studies, while 228 potential related studies were initially included. After analyzing the full texts and supplementary materials, 53 RCT studies [19–71] were ultimately included for data extraction and statistical analysis. The flowchart of the literature retrieval and screening is presented in Fig. 1.

### Basic characteristics of the included literature

In 53 Phase II/III randomized controlled trials, which included 17,351 mCRC patients, 29 different treatment schemes were used. Among these schemes, more patients received FOLFOX/FOLFIRI + Bevacizumab and CAPOX (Table 1 and Supplementary Table S1). These studies were published between 2000 and 2023. Only two were single-center studies [23, 24], while the rest were multicenter studies.

**Table 1:** Basic characteristics of included studies

Study	Treatmentarms		Sample size		Gender(M/F)		Age(years)	
	T1	T2	T1	T2	T1	T2	T1	T2
Watanabe 2023 [19]	FOLFOX + Panitumumab	FOLFOX + Bevacizumab	400	402	148/252	134/258	66(32–79)	66(28–79)
Stintzing 2023 [20]	FOLFOXIRI + Cetuximab	FOLFOXIRI + Bevacizumab	72	35	40/32	14/21	62(31–78)	64(31–78)
Sastre 2021 [21]	FOLFIRI + Bevacizumab	FOLFIRI + Cetuximab	126	113	NA	NA	60(32–70)	60(32–70)
Heinemann 2021 [22]	FOLFIRI + Cetuximab	FOLFIRI + Bevacizumab	199	201	147/52	133/68	64(41–76)	64(31–76)
Wentao 2020 [23]	FOLFOX + Bevacizumab	FOLFOX	121	120	79/42	80/40	58(29–75)	59(24–72)
Sadahiro 2020 [24]	SIRI + Bevacizumab	FOLFIRI + Bevacizumab	51	47	33/18	28/19	65(23–79)	64(38–83)
Maiello 2020 [25]	FOLFOX + Bevacizumab	CAPOX + Bevacizumab	45	87	22/23	46/41	62(42–73)	66(32–77)
Aranda 2020 [26]	FOLFOX + Bevacizumab	FOLFOXIRI + Bevacizumab	177	172	119/58	118/54	59(53–65)	61(54–66)
Parikh 2019 [27]	FOLFOX + Bevacizumab	FOLFIRI + Bevacizumab	188	188	122/66	117/71	61(31–87)	61(34–81)
Oki 2019 [28]	FOLFOX + Bevacizumab	FOLFOX + Cetuximab	57	59	34/23	34/25	64(32–80)	65(42–79)
Modest 2019 [29]	FOLFOXIRI + Panitumumab	FOLFOXIRI	63	33	41/22	24/9	58(31–76)	60(32–77)
Hurwitz 2019 [30]	FOLFOX + Bevacizumab	FOLFOXIRI + Bevacizumab	95	185	59/36	103/82	58(34–73)	56(23–75)
Shukui 2018 [31]	FOLFOX + Cetuximab	FOLFOX	193	200	127/66	139/61	56(21–83)	56(21–78)
Nakayama 2018 [32]	CAPOX + Bevacizumab	CAPIRI + Bevacizumab	54	53	36/18	32/21	67(40–79)	69(43–82)
Rivera 2017 [33]	FOLFOX + Panitumumab	FOLFOX + Bevacizumab	88	82	58/30	56/26	62(23–82)	60(39–82)
Kwakman 2017 [34]	Capecitabine	S-1	81	80	56/25	45/35	73(66–78)	74(68–79)
Carrato 2017 [35]	FOLFOX + Panitumumab	FOLFIRI + Panitumumab	38	39	31/7	28/11	65(32–79)	63(37–83)
Yamazaki 2016 [36]	FOLFIRI + Bevacizumab	FOLFOX + Bevacizumab	197	198	104/93	122/76	63(33–75)	62(26–75)
Aparicio 2016 [37]	FULV	FOLFIRI	142	140	75/67	76/64	80(75–90)	80(75–92)
Yamazaki 2015 [38]	SOL	FOLFOX	56	49	33/23	23/26	61(27–77)	61(27–76)
Kim 2015 [39]	OS	CAPOX	42	44	28/14	27/17	67(46–83)	66(29–76)
Loupakis 2014 [40]	FOLFIRI + Bevacizumab	FOLFOXIRI + Bevacizumab	256	252	156/100	150/102	60(29–75)	61(29–75)
Douillard 2014 [41]	FOLFOX + Panitumumab	FOLFOX	546	550	362/184	332/218	62(27–85)	61(24–82)
Schmiegel 2013 [42]	CAPOX + Bevacizumab	CAPIRI + Bevacizumab	127	120	84/43	80/40	64(27–84)	65(30–82)
Hong 2013 [43]	Capecitabine	CAPOX	40	40	23/17	22/18	71(66–81)	72(65–79)
Ducreux 2013 [44]	CAPIRI + Bevacizumab	FOLFIRI + Bevacizumab	72	73	64/36	48/52	61(38–74)	61(24–75)
Cunningham 2013 [45]	Capecitabine + Bevacizumab	Capecitabine	140	140	84/56	84/56	76(70–87)	77(70–87)
Ychou 2013 [46]	FOLFOXIRI	FOLFIRI	30	32	18/12	12/20	64(43–74)	63(46–73)
		FOLFOX		30		24/6		59(39–80)
Souglakos 2012 [47]	FOLFIRI + Bevacizumab	CAPIRI + Bevacizumab	167	166	104/63	109/57	66(33–80)	67(26–80)
Pectasides 2012 [48]	CAPIRI + Bevacizumab	FOLFIRI + Bevacizumab	143	142	79/64	92/50	66(28–84)	66(32–80)
Hong 2012 [49]	OS	CAPOX	168	172	109/59	108/64	61(53–66)	60(52–66)
Moosmann 2011 [50]	CAPIRI + Cetuximab	CAPOX + Cetuximab	89	88	63/26	63/25	63(32–75)	62(38–77)
Guan 2011 [51]	FOLFIRI	FOLFIRI_Bevacizumab	64	139	36/28	70/69	50(22–72)	53(23–77)
Schalhorn 2011 [52]	FOLFIRI	IROX	238	241	158/80	177/64	63(32–79)	63(21–79)
Ducreux 2011 [53]	CAPOX	FOLFOX	156	150	100/56	90/60	66(32–83)	62(42–84)
Cassidy 2011 [54]	FOLFOX	FOLFOX + Bevacizumab	668	349	390/278	205/144	61(24–83)	60(19–82)
	CAPOX	CAPOX + Bevacizumab	667	350	399/268	213/137	61(18–83)	61(18–86)
Tebbutt 2010 [55]	Capecitabine	Capecitabine + Bevacizumab	156	157	98/58	102/55	69(37–86)	67(32–85)
Rosati 2010 [56]	CAPOX	CAPIRI	47	47	25/22	27/20	75(70–85)	74(70–90)
Cunningham 2009 [57]	FOLFOX	FULV	362	363	235/127	225/138	61(29–81)	62(29–81)
Bokemeyer 2009 [58]	FOLFOX	FOLFOX + Cetuximab	168	169	92/76	89/80	60(30–82)	62(24–82)
Aranda 2009 [59]	FOLFIRI	FUIRI	173	173	110/63	110/63	63(29–75)	63(28–75)
Hochster 2008 [60]	FOLFOX	CAPOX	49	48	57/43	65/35	62(35–79)	63(32–84)
	FOLFOX + Bevacizumab	CAPOX + Bevacizumab	71	72	61/39	58/42	64(31–83)	62(32–82)
Borner 2008 [61]	CAPOX	CAPOX + Cetuximab	37	37	21/16	23/14	63(47–80)	60(37–81)
Fuchs 2007 [62]	FOLFIRI	CAPIRI	144	145	92/52	79/66	61(31–87)	62(20–85)
		FOLFIRI + Bevacizumab		57		30/27		59(32–81)
Falcone 2007 [63]	FOLFIRI	FOLFOXIRI	122	122	69/53	75/47	64(21–75)	62(27–75)
Díaz-Rubio 2007 [64]	CAPOX	FUOX	171	171	107/64	100/71	64(32–80)	65(35–81)
Souglakos 2006 [65]	FOLFIRI	FOLFOXIRI	146	137	82/61	76/61	66(39–84)	66(25–82)
Hospers 2006 [66]	FULV	FOLFOX	151	151	88/63	100/51	62(28–84)	62(41–80)
Kabbinavar 2005 [67]	FULV	FULV + Bevacizumab	105	104	51/49	56/44	71	71
Hurwitz 2004 [68]	FOLFIRI	FOLFIRI + Bevacizumab	411	402	60/40	59/41	59	60
Goldberg 2004 [69]	FOLFIRI	FOLFOX	264	267	172/92	157/110	61(28–88)	61(28–88)
		IROX		264	172/92	161/103	61(28–88)	61(29–84)
Kabbinavar 2003 [70]	FULV	FULV + Bevacizumab	36	35	27/9	17/18	NA	NA
Giacchetti 2000 [71]	FULV	FOLFOX	100	100	36/64	34/66	61(29–74)	61(31–75)

*Note* NA, not applicable; M,Male; F,Female; FULV,5-fluorouracil plus leucovorin; SIRI, S-1 plus irinotecan; SOL, S-1 plus oxaliplatin plus leucovorin; OS, S-1 plus oxaliplatin; FUOX,5-fluorouracil plus oxaliplatin; CAPOX, capecitabine plus oxaliplatin; CAPIRI, capecitabine plus irinotecan; FOLFOX, 5-fluorouracil plus leucovorin plus oxaliplatin; FOLFOXIRI, 5-fluorouracil plus leucovorin plus oxaliplatin plus irinotecan; FUIRI, 5-fluorouracil plus irinotecan; FOLFIRI, 5-fluorouracil plus leucovorin plus irinotecan; IROX, irinotecan plus oxaliplatin

### Quality evaluation

42 studies mentioned correct randomization methods, while 11 studies had unclear randomization methods. Among them, 20 studies reported well-established allocation concealment schemes and were rated as low risk; 31 studies had unclear allocation schemes and were categorized as an unknown risk, and 2 studies did not use allocation concealment schemes, resulting in a high risk. In addition, 25 studies did not mention blinding, and 18 studies did not employ a method to establish blinding for practitioners and patients. Lastly, 50 studies did not deviate from established interventions, and 3 studies had an unknown bias. All study outcomes were measured appropriately, and the outcome data were complete. Furthermore, all outcome measures mentioned in the study design were reported (Supplementary Figures S1 and S2).

### Network meta-analysis of all AEs

#### Grade ≥ 3 AEs

**Fig. 2:**
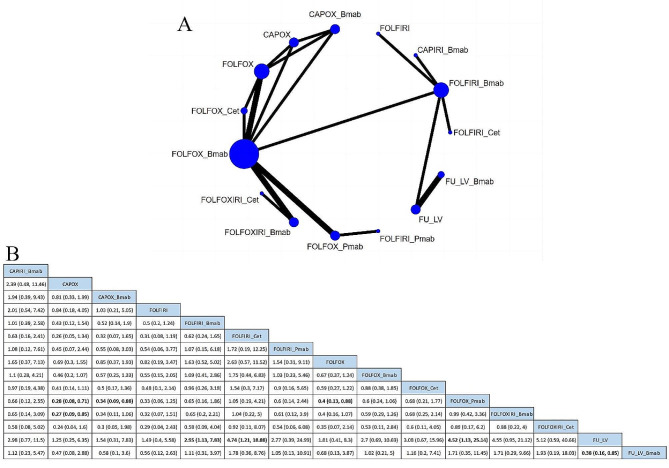
(**A**) Network plot of Grade ≥ 3 any adverse events. (**B**) risk ratios (95%CIs) of Grade ≥ 3 any adverse events

Seventeen studies compared the incidence of grade 3 or higher AEs across 15 treatment regimens (Fig. 2A). The RR was 0.38 (95% CrI, 0.16–0.85) for the FULV (5-fluorouracil plus leucovorin) vs. FULV + bevacizumab regimen and 0.27 (95% CrI, 0.01–0.85) for CAPOX vs. FOLFOXIRI + bevacizumab regimen, indicating that the FULV and CAPOX regimens had a lower risk of grade 3 or higher AEs, as shown in Fig. 2B. The top three safety regimens based on SUCRA values were FULV (89.76%), CAPOX (85.29%), and FOLFIRI (76.34%), while the least safe regimen was FOLFOX + panitumumab (19.91%), as illustrated in Supplementary Table S2.

#### Death related to AEs

**Fig. 3:**
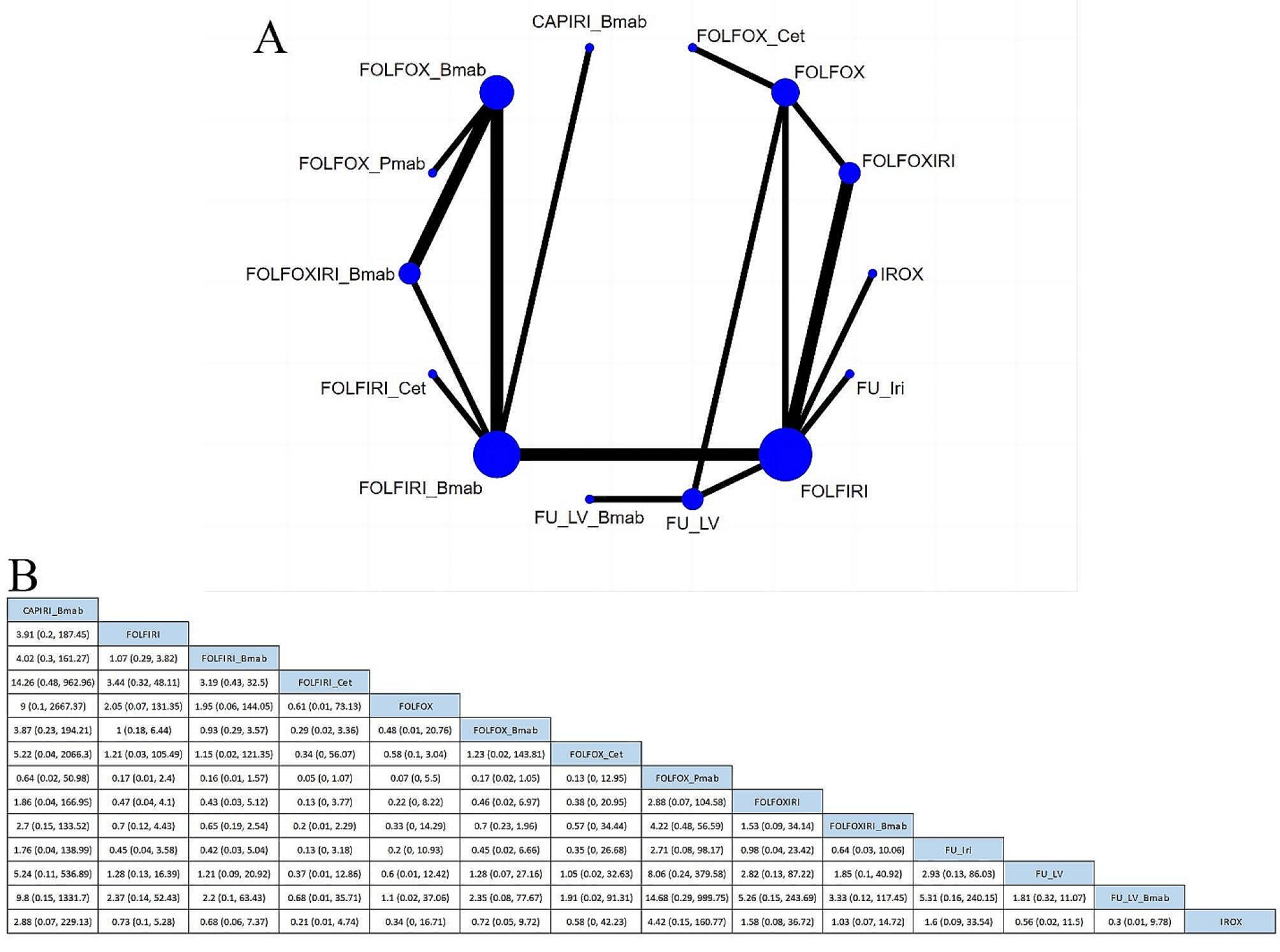
(**A**) Network plot of deaths related to adverse events. (**B**) risk ratios (95%CIs) of deaths related to adverse events

Eighteen studies compared the risk of death associated with AEs among 14 treatment regimens (Fig. 3A). Based on the safety ranking determined by the SUCRA value (where a higher SUCRA value indicates a lower incidence of AEs and a safer treatment regimen), the top three treatment plans were FOLFIRI + cetuximab (81.72%), FULV + bevacizumab (72.52%), and FOLFOX (68.53%). These results are presented in Supplementary Table S2. However, the league table results indicated that there was no statistically significant difference between these regimens (Fig. 3B).

### Network meta-analysis on specific AEs

#### Hematological AEs

##### Neutropenia

**Fig. 4:**
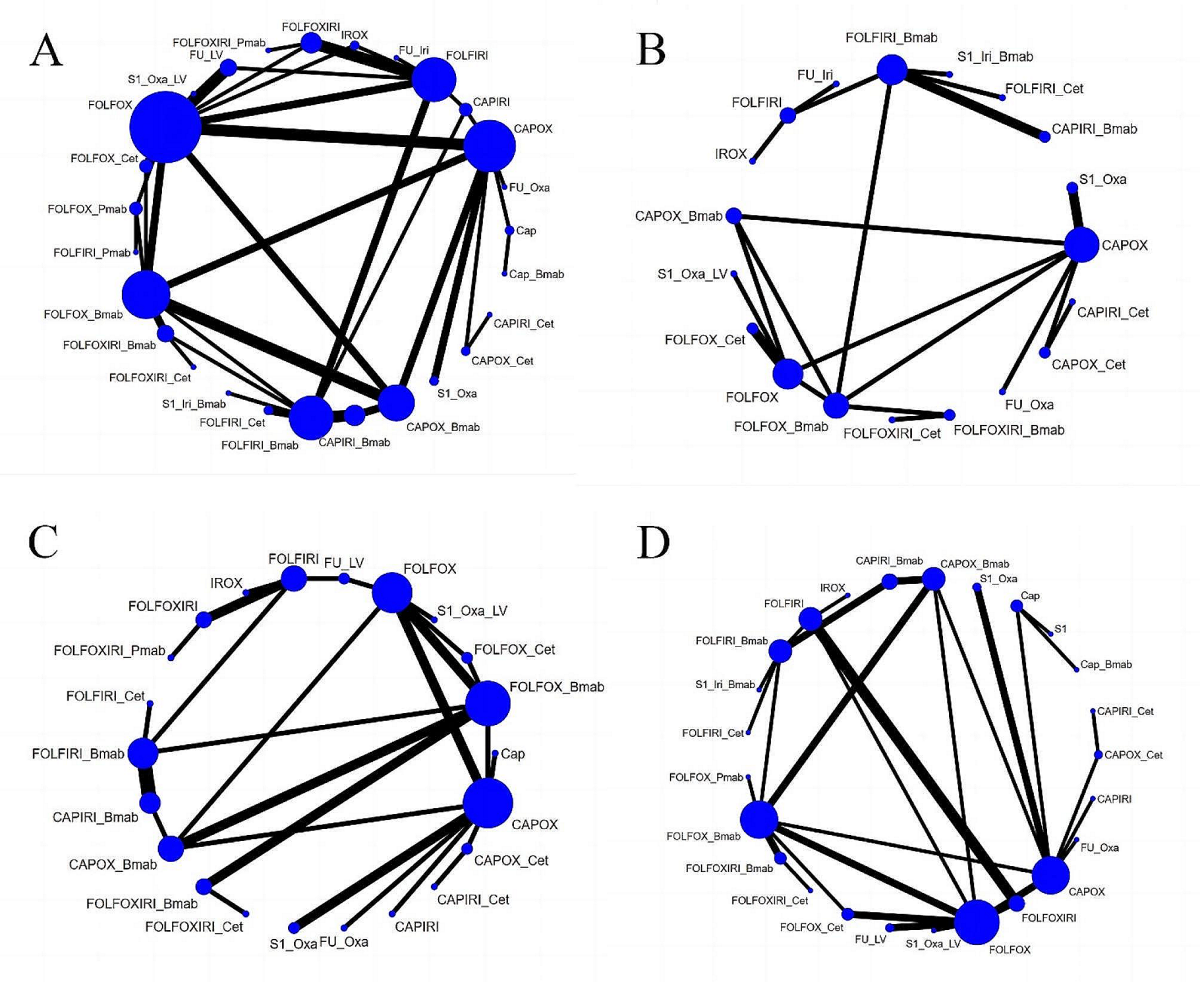
Network plot of hematological adverse events. (**A**) Neutropenia; (**B**) Leukopenia; (**C**) Anemia; (**D**)Thrombocytopenia. Bmab, bevacizumab; Cap, capecitabine; Cet, cetuximab; Oxa, oxaliplatin; Pmab, panitumumab; FU,5-fluorouracil; LV, leucovorin; Iri, irinotecan; CAPOX, capecitabine plus oxaliplatin; CAPIRI, capecitabine plus irinotecan; FOLFOX, 5-fluorouracil plus leucovorin plus oxaliplatin; FOLFOXIRI, 5-fluorouracil plus leucovorin plus oxaliplatin plus irinotecan; FOLFIRI, 5-fluorouracil plus leucovorin plus irinotecan; FUIRI, 5-fluorouracil plus irinotecan; IROX, irinotecan plus oxaliplatin. Each node represented a different treatment and its size depended on the number of patients that is directly examined. The nodes were joined by lines with different thickness which shows whether there was a direct relationship between treatments and the thickness was weighted according to the available direct evidence between them

Forty-four studies compared the incidence of neutropenia among 27 treatment regimens (Fig. 4A). The RR was 0.01 (95% CrI, 0–0.26) for CAPOX + cetuximab vs. FOLFOXIRI + cetuximab, 0.09 (95% CrI, 0.01–0.79) for capecitabine vs. CAPIRI (capecitabine plus irinotecan), and 0.1 (95% CrI, 0.05, 0.17) for CAPOX vs. FOLFOX. These results indicate that CAPOX + cetuximab, capecitabine, and CAPOX regimens had a lower risk of neutropenia, as depicted in Supplementary Figure S3. The top three regimens for safety based on SUCRA values were CAPOX + cetuximab (94.08%), capecitabine (89.93%), and CAPOX (83.63%). The least safe regimen was FOLFOXIRI + bevacizumab (6.55%), as shown in Table 2.

**Table 2:** SUCRA value of specific adverse events

Intervention	SUCRA (%)															Ranking
	Neutropenia	Febrile neutropenia	Leukopenia	Anemia	Thrombocytopenia	Diarrhea	Anorexia	Nausea	Vomiting	Mucositis/stomatitis	Fatigue	Peripheral sensory neuropathy	Hypertension	Thromboembolic events	Average	
CAPOX	83.63	91	75.44	60.55	24.51	39.79	NA	81.54	79.95	76.72	68.53	NA	74.07	NA	68.7	1
FULV	79.14	81.92	NA	55.71	44.23	82.28	NA	68.58	74.22	51.46	65.22	NA	NA	74.78	67.75	2
Cap	89.93	NA	NA	56.15	55.17	70.13	NA	73.79	NA	65.72	61.16	NA	NA	NA	67.44	3
FOLFIRI + Pmab	74.63	NA	NA	NA	NA	59.52	66.26	NA	NA	NA	NA	NA	NA	NA	66.8	4
Cap + Bmab	82.53	NA	NA	NA	53.95	63.6	NA	71.23	NA	73.45	50.37	NA	NA	NA	65.86	5
S-1	NA	NA	NA	NA	54.94	62.63	NA	78.27	NA	64.07	52.72	NA	NA	NA	62.53	6
OS	77.11	87.86	64.37	53.56	15.73	25.63	NA	90.57	78.5	70.94	49.71	NA	NA	NA	61.4	7
CAPOX + Cet	94.08	NA	90.41	84.12	60.5	28.67	NA	68.7	61.34	46.39	15.49	NA	NA	NA	61.08	8
CAPOX + Bmab	82.57	84.48	80.12	77.94	28.52	78	66.91	30.32	64.19	63.67	71.17	47.09	14.15	NA	60.7	9
CAPIRI + Cet	55.64	NA	81.34	56.77	78.26	36.13	NA	85.73	68.71	47.68	27.98	NA	NA	NA	59.8	10
FOLFOX	28.08	59.39	61.15	NA	47.93	62.77	NA	67.16	71.49	68.16	62.23	26.22	69.86	89.2	59.47	11
FUOX	73.82	NA	62.45	72.45	27.63	28.39	NA	73.45	61.86	59.37	NA	NA	NA	NA	57.43	12
FOLFOX + Bmab	33.89	69.13	47.87	68.09	50.09	83.63	73.69	50.16	61.8	53.61	63.66	35.85	26.21	61.62	55.66	13
FULV + Bmab	NA	NA	NA	NA	NA	82.64	NA	NA	NA	51.7	NA	NA	NA	22.23	52.19	14
FOLFIRI + Bmab	31.54	50.23	24.88	45.29	79.98	66.62	55.1	47.61	48.33	50.2	39.68	86.12	40.31	28.9	49.63	15
CAPIRI + Bmab	48.96	24.1	23.41	46.65	84.43	63.51	43.33	26.93	46.69	65.35	35.6	88.87	34	NA	48.6	16
FOLFOX + Pmab	32.62	56.42	NA	NA	32.95	53.85	41.94	NA	77.98	21.33	40.18	40.83	80.96	NA	47.91	17
FOLFIRI + Cet	29.69	13.54	18	29.38	72.39	20.93	NA	57.52	85.81	71.48	55.46	82.62	37.15	37.9	47.07	18
FOLFOX + Cet	15.49	63.43	53.52	50.86	36	48.42	NA	47.89	59.14	16.71	43.22	36.12	88.06	NA	46.57	19
FUIRI	64.3	37.71	64.86	NA	NA	33.04	NA	19.49	40.82	56.05	NA	NA	NA	NA	45.18	20
FOLFIRI	33.41	22.03	32.82	39.49	60.61	52.38	NA	37.03	47.22	49.25	NA	NA	80.54	35.36	44.56	21
CAPIRI	53.84	40.86	NA	47.21	46.23	16.85	NA	23.94	33.1	50.96	NA	NA	NA	NA	39.12	22
SOL	59.04	74.88	51.05	22.18	64.68	34.36	NA	NA	2.59	30.3	38.79	2.98	NA	NA	38.09	23
SIRI + Bmab	21.69	NA	20.76	NA	73.87	46.5	15.18	46.65	19.19	51.66	43.75	NA	41.06	NA	38.03	24
FOLFOXIRI + Bmab	6.55	36.15	30.48	71.24	23.98	55.18	37.6	32.97	41.52	31.07	43.07	53.31	13.64	NA	36.67	25
FOLFOXIRI + Cet	17.74	17.88	52.58	35.9	57.25	56.8	NA	8.98	17.38	16.86	72.03	NA	NA	NA	35.34	26
IROX	40.56	25.28	14.49	37.65	27.63	43.03	NA	24.62	35.62	40.08	NA	NA	NA	NA	32.11	27
FOLFOXIRI	12.4	13.71	NA	16.05	51.51	33.96	NA	27.64	15.01	38.58	NA	NA	NA	NA	26.11	28
FOLFOXIRI + Pmab	27.12	NA	NA	18.87	NA	20.78	NA	9.25	7.53	17.18	NA	NA	NA	NA	16.79	29

*Note* NA, not applicable; Cap, capecitabine; Cet, cetuximab; Bmab, bevacizumab; Oxa, oxaliplatin; Pmab, panitumumab; FULV,5-fluorouracil plus leucovorin; SIRI, S-1 plus irinotecan; SOL, S-1 plus oxaliplatin plus leucovorin; OS, S-1 plus oxaliplatin; FUOX,5-fluorouracil plus oxaliplatin; CAPOX, capecitabine plus oxaliplatin; CAPIRI, capecitabine plus irinotecan; FOLFOX, 5-fluorouracil plus leucovorin plus oxaliplatin; FOLFOXIRI, 5-fluorouracil plus leucovorin plus oxaliplatin plus irinotecan; FUIRI, 5-fluorouracil plus irinotecan; FOLFIRI, 5-fluorouracil plus leucovorin plus irinotecan; IROX, irinotecan plus oxaliplatin

##### Febrile neutropenia

Twenty-seven studies compared the incidence of febrile neutropenia among 19 treatment regimens (Supplementary Figure S4A). The RR was 0.15 (95% CrI, 0.05–0.36) for CAPOX vs. FOLFOXIRI, 28.45 (95% CrI, 1.85–437.2) for FOLFIRI vs. S-1 + oxaliplatin, and 0.11 (95% CrI, 0.02–0.41) for CAPOX + bevacizumab vs. FOLFOXIRI + bevacizumab, indicating a lower risk of febrile neutropenia for CAPOX, S-1 + oxaliplatin, and CAPOX + bevacizumab regimens, as shown in Supplementary Figure S5. The top three regimens for safety, based on SUCRA values, were CAPOX (91%), S-1 + oxaliplatin (87.86%), and CAPOX + bevacizumab (84.48%). The least safe regimen was FOLFIRI + cetuximab (13.54%), as indicated in Table 2.

##### Leukopenia

Eighteen studies compared the incidence of leukopenia among 19 treatment regimens (Fig. 4B). The RR was 0.01 (95% CrI, 0–0.68) for CAPOX + cetuximab vs. FOLFOXIRI + cetuximab, and 0.05 (95% CrI, 0–0.85) for CAPOX + bevacizumab vs. FOLFIRI + bevacizumab, indicating that CAPOX + cetuximab and CAPOX + bevacizumab had a lower risk of leukopenia, as depicted in Supplementary Figure S6. The top three regimens for safety, based on SUCRA values, were CAPOX + cetuximab (90.41%), CAPIRI + cetuximab (81.34%), and CAPOX + bevacizumab (80.12%). The least safe regimen was IROX (14.49%, irinotecan plus oxaliplatin), as shown in Table 2.

##### Anemia

Thirty studies compared the incidence of anemia among 22 treatment regimens (Fig. 4C). The top three regimens for safety, based on SUCRA values, were CAPOX + cetuximab (84.12%), CAPOX + bevacizumab (77.94%), and 5-FU + oxaliplatin (72.45%). The least safe regimen was FOLFOXIRI (16.05%), as indicated in Table 2. However, the league table results indicated that there was no statistical difference between the protocols (Supplementary Figure S7).

##### Thrombocytopenia

Thirty-four studies compared the incidence of thrombocytopenia among 25 treatment regimens (Fig. 4D). The RR was 0.04 (95% CrI, 0–0.39) for CAPIRI + bevacizumab vs. CAPOX + bevacizumab, and 0.05 (95% CrI, 0–0.84) for FOLFIRI + bevacizumab vs. FOLFOXIRI + bevacizumab, indicating that CAPIRI + bevacizumab and FOLFIRI + bevacizumab had a lower risk of thrombocytopenia, as shown in Supplementary Figure S8. The top three regimens for safety, based on SUCRA values, were CAPIRI + bevacizumab (84.43%), FOLFIRI + bevacizumab (79.98%), and CAPIRI + cetuximab (78.26%). The least safe regimen was S-1 + oxaliplatin (15.73%), as indicated in Table 2.

#### Gastrointestinal AEs

##### Diarrhea

**Fig. 5:**
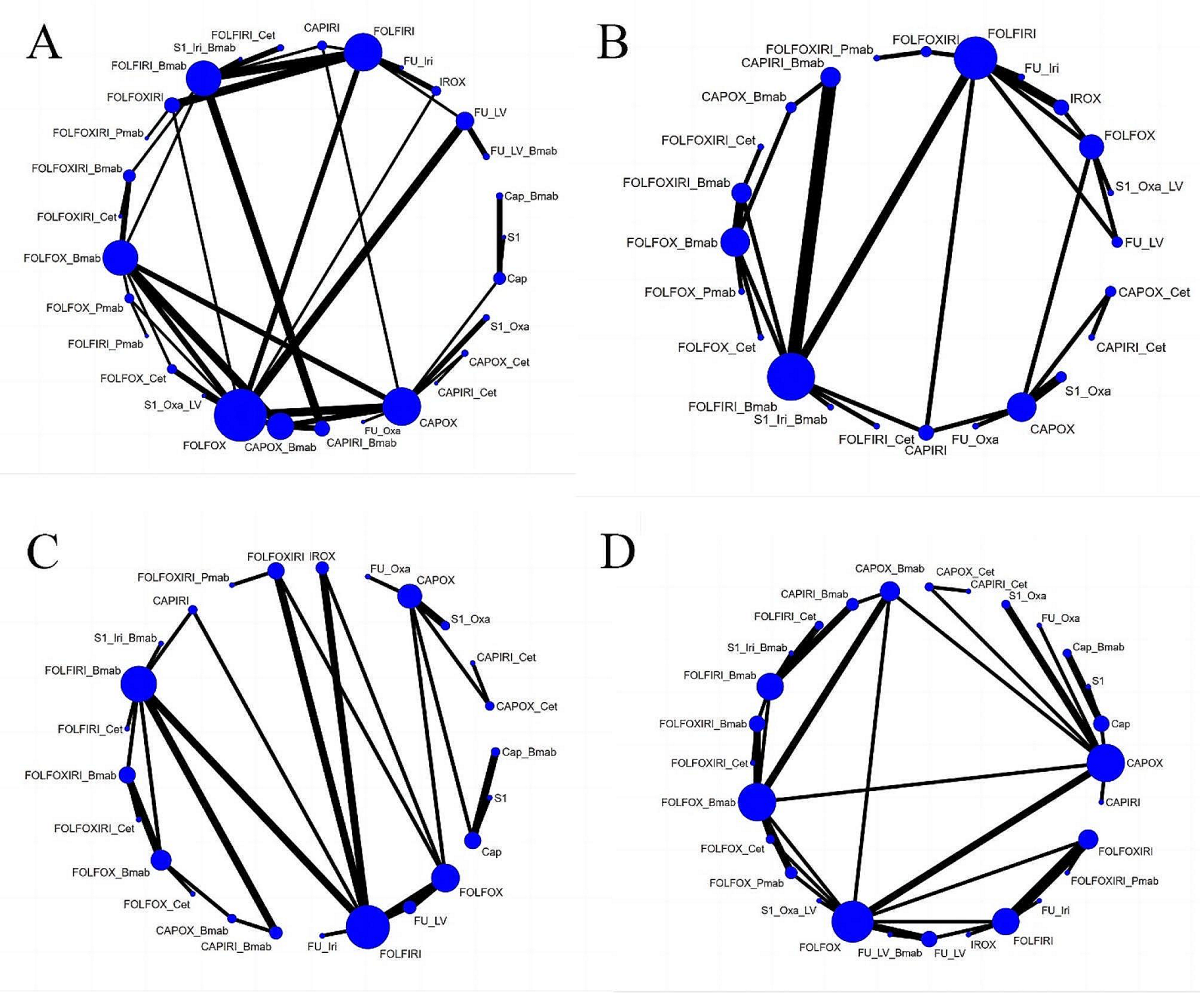
Network plot of Digestive adverse events. (**A**) Diarrhea; (**B**) Nausea; (**C**) Vomiting; (**D**) Mucositis/stomatitis. Bmab, bevacizumab. Cap, capecitabine; Cet, cetuximab; Oxa, oxaliplatin; Pmab, panitumumab; FU,5-fluorouracil; LV, leucovorin; Iri, irinotecan; CAPOX, capecitabine plus oxaliplatin; CAPIRI, capecitabine plus irinotecan; FOLFOX, 5-fluorouracil plus leucovorin plus oxaliplatin; FOLFOXIRI, 5-fluorouracil plus leucovorin plus oxaliplatin plus irinotecan; FOLFIRI, 5-fluorouracil plus leucovorin plus irinotecan; FUIRI, 5-fluorouracil plus irinotecan; IROX, irinotecan plus oxaliplatin. Each node represented a different treatment and its size depended on the number of patients that is directly examined. The nodes were joined by lines with different thickness which shows whether there was a direct relationship between treatments and the thickness was weighted according to the available direct evidence between them

Fifty-one studies compared the incidence of diarrhea among 29 treatment regimens (Fig. 5A). The RR was 0.15 (95% CrI, 0.02–0.95) for FOLFOX + bevacizumab vs. FOLFOXIRI regimen, 20.1 (95% CrI, 1.42–279.88) for CAPIRI vs. FULV + bevacizumab regimen, and 5.19 (95% CrI, 1.01–27.99) for CAPOX vs. FULV regimen. These results indicate that FOLFOX + bevacizumab, FULV + bevacizumab, and FULV regimens had a lower risk of diarrhea, as shown in Supplementary Figure S9. The top three safety regimens, based on SUCRA values, were FOLFOX + bevacizumab (83.63%), FULV + bevacizumab (82.64%), and FULV (82.28%). The least safe regimen was CAPIRI (16.85%), as illustrated in Table 2.

##### Nausea

Thirty-one studies compared the incidence of nausea among 24 treatment regimens (Fig. 5B). The RR was 0.02 (95% CrI, 0–0.47) for FOLFIRI + cetuximab vs. FOLFOXIRI + cetuximab regimen, 0.03 (95% CrI, 0–0.48) for CAPOX vs. FOLFOXIRI regimen, and 39.22 (95% CrI, 1.54–2529.6) for FOLFOXIRI vs. S-1 + oxaliplatin. These results indicated that FOLFIRI + cetuximab, CAPOX, and S-1 + oxaliplatin regimens had a lower risk of nausea, as shown in Supplementary Figure S10. The top three safety regimens, based on SUCRA values, were FOLFIRI + cetuximab (85.81%), CAPOX (79.95%), and S-1 + oxaliplatin (78.5%). The least safe regimen was S-1 + oxaliplatin + LV (2.59%), as illustrated in Table 2.

##### Vomiting

Thirty-one studies compared the incidence of vomiting among 25 treatment regimens (Fig. 5C). The RR was 42.05 (95% CrI, 3.95–566.39) for IROX vs. S-1 + oxaliplatin, 0 (95% CrI, 0–0.51) for CAPIRI + cetuximab vs. FOLFOXIRI + cetuximab, and 0.06 (95% CrI, 0–0.85) for CAPOX vs. CAPOX + bevacizumab regimens. These results indicate that S-1 + oxaliplatin, CAPIRI + cetuximab, and CAPOX regimens had a lower risk of vomiting, as shown in Supplementary Figure S11. The top three regimens for safety, based on SUCRA values, were S-1 + oxaliplatin (90.57%), CAPIRI + cetuximab (85.73%), and CAPOX (81.54%). The least safe regimen was FOLFOXIRI + cetuximab (8.98%), as illustrated in Table 2.

##### Mucositis/stomatitis

Forty studies compared the incidence of mucositis or stomatitis among 28 treatment regimens (Fig. 5D). The RR was 0.06 (95% CrI, 0–0.62) for CAPOX vs. FOLFOX + cetuximab regimen and 0.14 (95% CrI, 0.03–0.55) for FOLFOX vs. FOLFOX + panitumumab. These results indicate a lower risk of mucositis or stomatitis for CAPOX and FOLFOX regimens, as shown in Supplementary Figure S12. The top three treatment regimens for safety, based on SUCRA values, were CAPOX (76.72%), Cap + bevacizumab (73.45%), and FOLFIRI + cetuximab (71.48%). The least safe regimen was FOLFOX + Cetuximab (16.71%), as illustrated in Table 2.

##### Anorexia

Ten studies compared the incidence of anorexia among 8 treatment regimens (Supplementary Figure S4B). The top three treatment regimens for safety, based on SUCRA values, were FOLFOX + bevacizumab (73.69%), CAPOX + bevacizumab (66.91%), and FOLFIRI + panitumumab (66.26%). The least safe regimen was S-1 + Iri + bevacizumab (15.18%), as illustrated in Table 2. However, the league table results showed that there was no statistical difference between the protocols (Supplementary Figure S13).

#### Neurological AEs

##### Peripheral sensory neuropathy

**Fig. 6:**
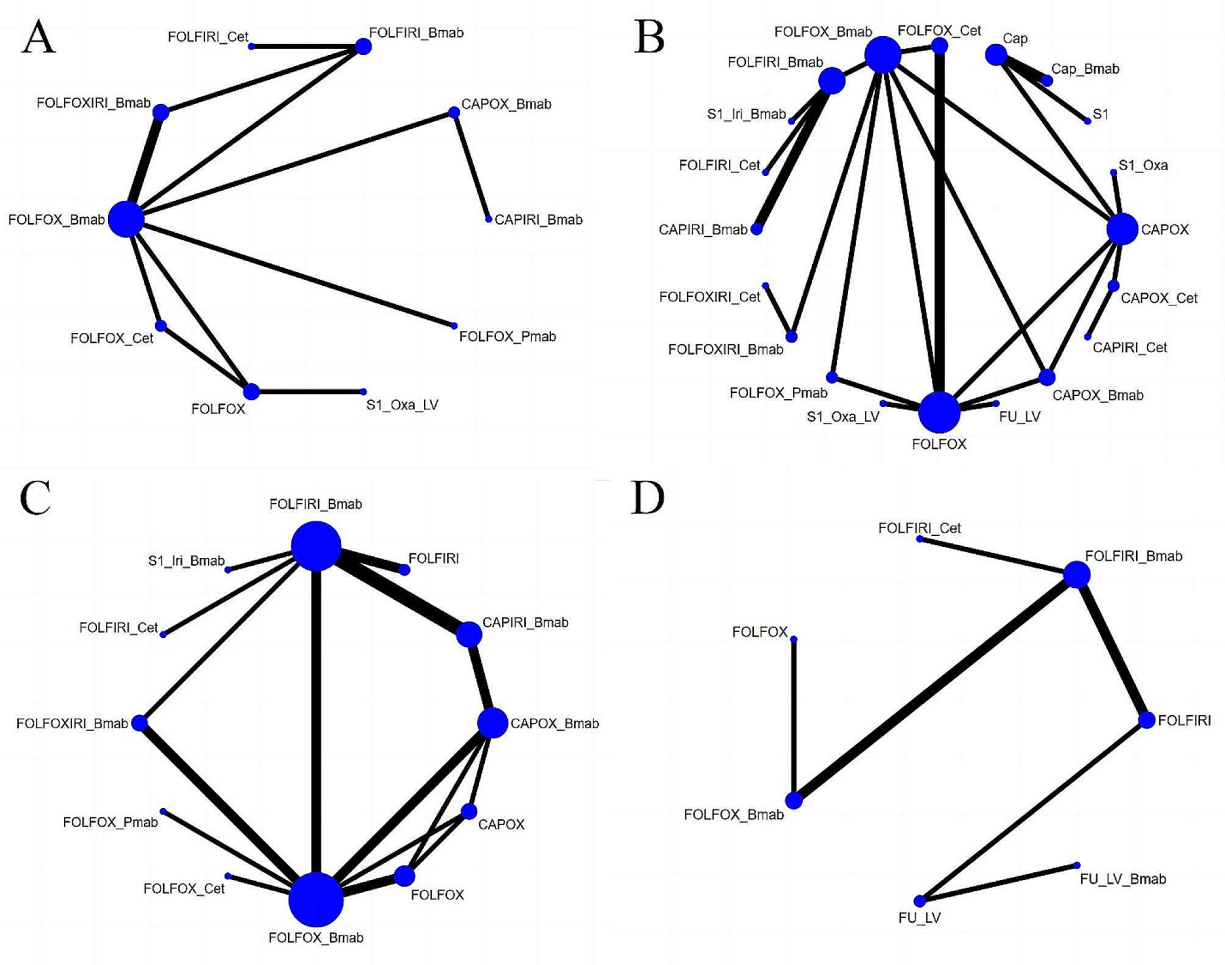
Network plot of Neurological adverse events. (**A**) Peripheral sensory neuropathy; (**B**) Fatigue; (**C**) Hypertension; (**D**) Thromboembolic events. Bmab, bevacizumab. Cap, capecitabine; Cet, cetuximab; Oxa, oxaliplatin; Pmab, panitumumab; FU,5-fluorouracil; LV, leucovorin; Iri, irinotecan; CAPOX, capecitabine plus oxaliplatin; CAPIRI, capecitabine plus irinotecan; FOLFOX, 5-fluorouracil plus leucovorin plus oxaliplatin; FOLFOXIRI, 5-fluorouracil plus leucovorin plus oxaliplatin plus irinotecan; FOLFIRI, 5-fluorouracil plus leucovorin plus irinotecan; FUIRI, 5-fluorouracil plus irinotecan; IROX, irinotecan plus oxaliplatin. Each node represented a different treatment and its size depended on the number of patients that is directly examined. The nodes were joined by lines with different thickness which shows whether there was a direct relationship between treatments and the thickness was weighted according to the available direct evidence between them

Twelve studies compared the risk of peripheral sensory neuropathy among 10 treatment regimens (Fig. 6A). The RR was 0.02 (95% CrI, 0–0.32) for CAPIRI + bevacizumab vs. CAPOX + bevacizumab, 0.02 (95% CrI, 0–0.16) for FOLFIRI + bevacizumab vs. FOLFOX + bevacizumab, and 0.04 (95% CrI, 0–0.38) for FOLFOXIRI + bevacizumab. These results indicate a lower risk of peripheral sensory neuropathy for CAPIRI + bevacizumab and FOLFIRI + bevacizumab regimens, as shown in Supplementary Figure S14. The top three treatment regimens for safety, based on SUCRA values, were CAPIRI + bevacizumab (88.87%), FOLFIRI + bevacizumab (86.12%), and FOLFIRI + cetuximab (82.62%). The lowest-ranked regimen was S-1 + oxaliplatin + LV (2.98%), as illustrated in Table 2.

##### Fatigue

Twenty-two studies compared the incidence of fatigue among 20 treatment regimens (Fig. 6B). CAPOX was associated with a lower risk of fatigue compared to CAPOX + Cetuximab (RR = 0.13, 95% CI: 0.02–0.83), as shown in Supplementary Figure S15. The top three regimens for safety, based on SUCRA values, were FOLFOXIRI + cetuximab (72.03%), CAPOX + bevacizumab (71.17%), and CAPOX (68.53%). The last-ranked regimen was CAPOX + Cetuximab (15.49%), as illustrated in Table 2.

#### Circulatory AEs

##### Hypertension

Nineteen studies compared the incidence of hypertension among 13 treatment regimens (Fig. 6C). Compared with FOLFOX + cetuximab (RR = 15.8, 95% CI: 2.05-436.84) and FOLFOX + panitumumab (RR = 9.65, 95% CI: 1.14, 323.19), FOLFOX + bevacizumab had a higher risk of hypertension. Compared with FOLFIRI + Bevacizumab (RR = 0.17, 95% CI: 0.06, 0.41) and FOLFIRI + cetuximab (RR = 0.16, 95% CI: 0.04, 0.66), FOLFIRI had a lower risk of hypertension. The results are shown in Supplementary Figure S16. The top three treatment regimens for safety, based on SUCRA values, were FOLFOX + cetuximab (88.06%), FOLFOX + panitumumab (80.96%), and FOLFIRI (80.54%). The last-ranked regimen was FOLFOXIRI + Bevacizumab (13.64%), as illustrated in Table 2.

##### Thromboembolic events

Eight studies compared the incidence of thromboembolic events among 7 treatment regimens (Fig. 6D). The top three treatment regimens for safety, based on SUCRA values, were FOLFOX (89.2%), FULV (74.78%), and FOLFOX + bevacizumab (61.62%), with the last regimen being FULV + bevacizumab (22.23%), as illustrated in Table 2. However, the league table results indicated no statistical difference between the protocols (Supplementary Figure S17).

### Consistency and publication bias assessment

DIC was employed to compare the consistency model with the inconsistency model. All closed-loop models exhibited variation values of less than 5, indicating good consistency, as indicated by DIC. The local inconsistency test between direct evidence and indirect evidence revealed local inconsistency between CAPIRI + Bevacizumab and CAPOX + Bevacizumab (*P* = 0.01) and FOLFIRI + Bevacizumab (*P* = 0.01) in terms of thrombocytopenia, as shown in Supplementary Table S3. Regarding the assessment of publication bias, no evidence of publication bias was observed in the comparison-adjusted funnel plots (Supplementary Figures S18 and S19).

## Discussion

In this study, we utilized the NMA method to compare and rank first-line systemic treatment regimens for mCRC patients, analyzing the specific AEs associated with each regimen. This approach aims to identify treatment regimens with high safety profiles and clarify the toxicity characteristics of each treatment regimen. Such findings are essential for offering guidance in clinical decision-making and hold significant practical values in enhancing the treatment outcomes and quality of life for mCRC patients.

Based on the NMA results, the CAPOX regimen ranked first in the SUCRA comprehensive rankings of AEs. It may currently be the safest regimen for first-line systemic treatment of mCRC patients. However, the analysis of specific AEs revealed that the CAPOX regimen was associated with thrombocytopenia and diarrhea. Degirmencioglu et al. [72] also showed that the CAPOX regimen was inferior to the FOLFOX regimen in terms of disease progression, metastasis, and mortality in patients with colorectal cancer. There was no significant difference between the two regimens in overall survival. An observational study from India indicated that the CAPOX regimen was more effective but was prone to grade 3/4 blood and gastrointestinal toxicities such as thrombocytopenia (11.7%) and diarrhea (5.5%), consistent with our findings [73]. Additionally, CAPOX regimens were well-tolerated in febrile neutropenia, neutropenia, and leukopenia. A meta-analysis concluded that oxaliplatin-based regimens were better tolerated regarding leukopenia and febrile neutropenia compared to irinotecan-based regimens [74]. Two RCTs [50, 58] also demonstrated that neutropenia and leukopenia were less likely to occur with CAPOX than FOLFOX. S-1 is an oral fluoropyrimidine chemotherapy agent designed to enhance the antitumor activity of 5-fluorouracil while reducing its severe gastrointestinal toxicity, including nausea, vomiting, stomatitis, and diarrhea [75]. Our study also found that S-1 or S-1 + Oxaliplatin regimens were well-tolerated in terms of gastrointestinal response.

FULV secured the second position in the SUCRA comprehensive rankings for AEs, indicating good safety. When comparing single-drug chemotherapy with combined chemotherapy or chemotherapy combined with targeted drugs, it becomes apparent that while they significantly enhance the anti-tumor effect, they also increase the occurrence of side effects. In a detailed analysis of specific AEs, both the FOLFOX and FOLFOX + bevacizumab regimens were associated with neutropenia and peripheral sensory neuropathy. Furthermore, the FOLFOX + bevacizumab regimens were linked to hypertension. FOLFOX exhibited a lower risk of hypertension compared to FOLFOX + bevacizumab (RR = 0.22 (95% CrI: 0.05–0.73). This finding was consistent with results from two RCTs [23, 60], which demonstrated that FOLFOX + bevacizumab was more likely to result in hypertension when directly compared with FOLFOX. This aligns with previous studies, which identified hypertension as the most common side effect of bevacizumab [76–78]. The FOLFOX + panitumumab and FOLFOX + cetuximab regimens were associated with multiple AEs. A meta-analysis assessing the safety of panitumumab in CRC patients revealed a higher frequency of grade 3/4 AEs in the panitumumab group when compared to the control group (RR = 1.17, 95% CI: 1.08–1.27, *P* = 0.0001) [79]. Concerning the toxic side effects of cetuximab, a separate meta-analysis suggested that cetuximab was linked to an increased risk of leukopenia, neutropenia, and anemia events in patients with colorectal cancer [80]. Another meta-analysis reported that cetuximab carried a greater risk of skin diseases compared to bevacizumab [81]. One study demonstrated a significantly higher incidence of hand and foot skin reactions (*P* = 0.02) in patients treated with CAPIRI + bevacizumab compared with patients treated with FOLFIRI + bevacizumab [47] .In addition, two RCTs also demonstrated that FOLFOX + cetuximab was associated with a higher incidence of grade 3 or higher AEs when compared to FOLFOX with or without bevacizumab [28, 58].

Irinotecan-based regimens, specifically FOLFIRI + bevacizumab and CAPIRI + bevacizumab, were well tolerated with regards to thrombocytopenia and peripheral sensory neuropathy. A meta-analysis indicated that irinotecan-based regimens were associated with a lower risk of peripheral sensory neuropathy and thrombocytopenia compared to oxaliplatin-based regimens [74]. Additionally, two RCTs provided evidence that CAPIRI + bevacizumab was less likely to cause peripheral sensory neuropathy and thrombocytopenia compared to CAPOX + bevacizumab [32, 42]. An RCT study demonstrated that FOLFIRI + bevacizumab was directly linked to a reduction in peripheral sensory neuropathy when compared with FOLFOX + bevacizumab [36]. According to the SUCRA ranking, the FOLFOXIRI scheme was more likely to result in grade 3 or higher AEs compared to FOLFIRI. Consistently, a meta-analysis showed that the incidence of neutropenia, anemia, diarrhea, stomatitis, and neuropathy in the FOLFOXIRI group was significantly higher than that in the FOLFIRI group [81], which aligns with the results of this study. Furthermore, three RCTs [26, 30, 40] also indicated that FOLFOXIRI + bevacizumab might increase the risk of grade 3 or higher AEs when directly compared with FOLFOX/FOLFIRI + bevacizumab.

As far as we know, this study possesses several notable advantages. Firstly, it employed network meta-analysis to directly and indirectly compare multiple interventions, addressing the limitation of traditional meta-analysis that only allows the analysis of two directly compared interventions. Secondly, this study evaluated the risk of AEs across different treatment regimens, providing a clearer understanding of the toxicity profile associated with each regimen. Thirdly, in comparison to other NMAs assessing the safety of first-line systemic treatment for mCRC [8], this study included and analyzed a more extensive range of treatment regimens and outcome indicators. It also incorporated and assessed the latest relevant studies, offering a robust reference for clinical treatment regimen selection. However, this study has acknowledged limitations. Firstly, due to the absence of head-to-head trials, some treatment options were excluded from the network for different outcomes. Secondly, our study did not analyze results according to the RAS and BRAF status or left/right status. This was primarily because among the 53 studies included, only two RCT studies [19, 22] compared treatment outcomes for left and right colon cancer, and 13 RCT studies mentioned the RAS and BRAF status. Consequently, we cannot fully evaluate all treatment options for mCRC based on RAS and BRAF status or left/right status. Thirdly, the different dosage forms of treatment regimens may affect the final assessment. Fourthly, for four outcome measures (i.e., death related to AEs, anemia, anorexia, and thromboembolic event), ranking guidance may be limited due to the absence of statistical differences between treatment regimens. Fifthly, there was local inconsistency in the indicator of thrombocytopenia. After a thorough examination and analysis of the original text, it was discovered that the number of responders in the CAPIRI + Bevacizumab regimen was frequently recorded as 0 for this indicator, potentially contributing to outcome inconsistency. Sixth, during literature screening, we only included studies where the language was English, which may have increased the bias of the results.

## Conclusion

Based on SUCRA rankings, the CAPOX regimen is most likely to secure the top position in terms of safety, while the FOLFOXIRI + panitumumab regimen is most likely to rank last. In the analysis of specific AEs, The CAPOX regimen, whether combined with or without targeted drugs (bevacizumab and cetuximab), is associated with a reduced risk of neutropenia and febrile neutropenia, as well as an increased risk of thrombocytopenia and diarrhea. The FOLFOX regimen, with or without bevacizumab, is linked to an increased incidence of neutropenia and peripheral sensory neuropathy. The FOLFIRI/CAPIRI + bevacizumab regimen is connected to a decrease in the risk of peripheral sensory neuropathy. S-1 and S-1 + oxaliplatin are well tolerated with respect to gastrointestinal reactions. The FOLFOXIRI regimen, whether combined with or without targeted drugs, is associated with various AEs.

In summary, the CAPOX regimen appears to be the safest option among the first-line systemic treatment regimens for mCRC patients, while the FOLFOXIRI + panitumumab regimen may be associated with a higher incidence of grade 3 or higher AEs. More pharmacological and clinical trials are required to optimize first-line systemic regimens, enhancing efficacy and minimizing toxicity. Additionally, further well-designed, high-quality RCTs are anticipated to validate our findings.

### Electronic supplementary material

Below is the link to the electronic supplementary material.

Supplementary Material 1

Supplementary Material 2

Supplementary Material 3

## Data Availability

The original contributions presented in the study are included in the article/supplementary material, further inquiries can be directed to the corresponding author.
