# Supplementary material for: Safety of first-line systemic therapy in patients with metastatic colorectal cancer: a network meta-analysis of randomized controlled trials
Source: BMC Cancer. 2024 Jul 24;24:893. doi: 10.1186/s12885-024-12662-3 (PMC11270896; doi:10.1186/s12885-024-12662-3)
Supplement: Supplementary file 1 — Supplementary Material 1 [file 12885_2024_12662_MOESM1_ESM.docx]

**Supplementary Tables**

**Supplementary Table S1 Other characteristics of included studies**

| Study | Specificname | Studyperiod | Country (Phase) | Follow-uptime(months) |
| --- | --- | --- | --- | --- |
| Watanabe 2023[19] | NCT02394795 | 05/2015-06/2017 | Japan(phaseIII) | 61 |
| Stintzing 2023[20] | FIRE-4.5(AIOKRK0116) | 11/2016-12/2020 | Germany(PhaseII) | NA |
| Sastre 2021[21] | NCT01640444 | 10/2012-11/2016 | Spain(PhaseII) | 44 |
| Heinemann 2021[22] | FIRE-3(NCT00433927) | 01/2007-09/2012 | Germany,Austria(phaseIII) | 71 |
| Wentao 2020[23] | NCT01972490 | 10/2013-12/2017 | China(phaseII) | 37 |
| Sadahiro 2020[24] | UMIN-CRT(00001464) | 12/2013-01/2018 | Japan(PhaseII) | 23 |
| Maiello 2020[25] | GOIM2802(EudraCT:2010-022091-31) | 06/2011-10/2015 | Italy(PhaseII) | 48 |
| Aranda 2020[26] | VISNÚ-1(NCT01640405) | 10/2012-11/2016 | Spain(phaseIII) | 52 |
| Parikh 2019[27] | MAVERICC | NA | America(PhaseII) | 18 |
| Oki 2019[28] | ATOM(UMIN-CTR:UMIN000010209) | 05/2013-04/2016 | Japan(PhaseII) | 24 |
| Modest 2019[29] | AIOKRK0109(NCT01328171) | 06/2011-01/2016 | Germany(PhaseII) | 54 |
| Hurwitz 2019[30] | STEAM(NCT01765582) | 01/2013-12/2014 | America(PhaseII) | 21 |
| Shukui 2018[31] | TAILOR(NCT01228734) | 09/2010-01/2016 | China(phaseIII) | 47 |
| Nakayama 2018[32] | CCOG-1201(UMIN000006478) | 06/2012-04/2016 | Japan(PhaseII) | 36 |
| Rivera 2017[33] | PEAK(NCT00819780) | 04/2009-12/2011 | 6 countries(PhaseII) | 31 |
| Kwakman 2017[34] | SALTO(NCT01918852) | 01/2014-07/2015 | Netherlands(phaseIII) | 20 |
| Carrato 2017[35] | PLANET-TTD(NCT00885885) | 05/2009-11/2012 | Spain(PhaseII) | 33 |
| Yamazaki 2016[36] | WJOG4407G(UMIN000001396) | 09/2008-01/2012 | Japan(phaseIII) | 51 |
| Aparicio 2016[37] | FFCD2001-02(NCT00303771) | 06/2003-05/2010 | 19 countries(phaseIII) | 70 |
| Yamazaki 2015[38] | NCT00721916 | 07/2008-07/2009 | Japan(PhaseII) | 35 |
| Kim 2015[39] | NCT00677144 | 04/2008-08/2011 | SouthKorea(PhaseII) | 18 |
| Loupakis 2014[40] | NCT00719797 | 07/2008-05/2011 | Italy(phaseII) | 32 |
| Douillard 2014 [41] | PRIME(NCT00364013) | 08/2006-08/2010 | France(phaseIII) | 33 |
| Schmiegel 2013[42] | NA | 07/2005-10/2006 | Germany(phaseII) | 27 |
| Hong 2013[43] | CO06-01 | 05/2006-04/2008 | SouthKorea(phaseII) | 49 |
| Ducreux 2013[44] | FNCLCCACCORD13/0503(NCT00423696) | 03/2006-01/2008 | France(phaseII) | 36 |
| Cunningham 2013[45] | AVEX(NCT00484939) | 07/2007-12/2010 | 10 countries(phaseIII) | 12 |
| Ychou 2013[46] | METHEP(NCT00208260) | 10/2004-08/2007 | Greece(phaseII) | 50 |
| Souglakos 2012[47] | NCT00469443 | 06/2005-06/2008 | France(phaseII) | 32 |
| Pectasides 2012[48] | ACTRN12610000270011 | 01/2006-01/2008 | Greece(phaseIII) | 42 |
| Hong 2012[49] | NCT00677443 | 05/2008-09/2009 | SouthKorea(phaseIII) | 21 |
| Moosmann 2011[50] | AIOKRK-0104 | 09/2004-12/2006 | Germany(phaseII) | NA |
| Guan 2011[51] | ARTIST(BO20696;NCT00642577) | 07/2007-08/2008 | China(phaseIII) | NA |
| Schalhorn 2011[52] | NA | 07/2000-10/2004 | Germany(phaseIII) | 21 |
| Ducreux 2011[53] | ML16987 | 05/2003-08/2004 | France(phaseIII) | 19 |
| Cassidy 2011[54] | NO16966 | 07/2003-05/2004 | 41 countries(phaseIII) | 49 |
| Tebbutt 2010[55] | MAX | 07/2005-06/2007 | 3 countries(phaseIII) | 31 |
| Rosati 2010[56] | NA | 12/2005-01/2008 | Italy(phaseII) | 18 |
| Cunningham 2009[57] | NA | NA | Britain(PhaseIIIB) | 24 |
| Bokemeyer 2009[58] | NA | 07/2005-03/2006 | Germany(phaseII) | NA |
| Aranda 2009[59] | NA | 10/2001-10/2005 | Spain | 17 |
| Hochster 2008[60] | TREE-1 | 12/2002-11/2003 | America | 16 |
|  | TREE-2 | 1/2003-04/2004 |  | 18 |
| Borner 2008[61] | SAKK | 06/2004-10/2005 | Switzerland(phaseII) | 17 |
| Fuchs 2007[62] | BICC-C | 02/2003-03/2004 | 4 countries(phaseII) | 28 |
| Falcone 2007[63] | NA | 11/2001-04/2005 | Italy(phaseIII) | 18 |
| Díaz-Rubio 2007[64] | NA | 04/2002-08/2004 | Spain(PhaseIII) | 19 |
| Souglakos 2006[65] | HORG | 10/2000-12/2004 | Greece(phaseIII) | 36 |
| Hospers 2006[66] | NA | 07/1999-08/2002 | Netherlands(phaseIII) | 32 |
| Kabbinavar 2005[67] | NA | 08/2000-07/2002 | 3 countries(PhaseII) | NA |
| Hurwitz 2004[68] | NA | 09/2000-05/2002 | 3 countries(phaseIII) | NA |
| Goldberg 2004[69] | NA | 05/1999-04/2001 | 5 countries(phaseIII) | 20 |
| Kabbinavar 2003[70] | NA | 06/1998-11/1998 | America(PhaseII) | 22 |
| Giacchetti 2000[71] | NA | 06/1994-03/1996 | 4 countries(PhaseIII) | 47 |

**Supplementary Table S2 SUCRA value of all adverse events**

| Intervention | SUCRA(%) | | | Ranking |
| --- | --- | --- | --- | --- |
|  | Grade ≥3 any AEs | Death related to AEs | Average |  |
| CAPOX | 85.29 | NA | 85.29 | 1 |
| FOLFIRI | 76.34 | NA | 76.34 | 2 |
| CAPOX+Bevacizumab | 75.94 | 72.52 | 74.23 | 3 |
| FULV | 89.76 | 58.68 | 74.22 | 4 |
| FOLFOX | 68.68 | 55.1 | 61.89 | 5 |
| FULV+Bevacizumab | 47.98 | 68.53 | 58.255 | 6 |
| FOLFOX+Bevacizumab | 47.94 | 55.79 | 51.865 | 7 |
| FOLFIRI+Cetuximab | 21.08 | 81.72 | 51.4 | 8 |
| CAPIRI+Bevacizumab | 42.47 | 57.68 | 50.075 | 9 |
| FOLFOX+Cetuximab | 39.82 | 54.95 | 47.385 | 10 |
| FOLFIRI+Panitumumab | 46.93 | NA | 46.93 | 11 |
| IROX | NA | 45.24 | 45.24 | 12 |
| FOLFOXIRI | NA | 34.34 | 34.34 | 13 |
| FUIRI | NA | 33.63 | 33.63 | 14 |
| FOLFIRI+Bevacizumab | 42.72 | 24.48 | 33.6 | 15 |
| FOLFOXIRI+Bevacizumab | 21.25 | 42.71 | 31.98 | 16 |
| FOLFOXIRI+Cetuximab | 23.89 | NA | 23.89 | 17 |
| FOLFOX+Panitumumab | 19.91 | 14.63 | 17.27 | 18 |

Note: AEs,adverse events; FULV,5-fluorouracil plus leucovorin; CAPOX, capecitabine plus oxaliplatin; CAPIRI, capecitabine plus irinotecan; FOLFOX, 5-fluorouracil plus leucovorin plus oxaliplatin; FOLFOXIRI, 5-fluorouracil plus leucovorin plus oxaliplatin plus irinotecan; FUIRI, 5-fluorouracil plus irinotecan; FOLFIRI, 5-fluorouracil plus leucovorin plus irinotecan; IROX, irinotecan plus oxaliplatin.

**Supplementary Table S3 Local inconsistency**

| Outcome indicator | Intervention comparison | Direct comparison OR(95%Crl) | Indirect comparison OR(95%Crl) | p value |
| --- | --- | --- | --- | --- |
| Death related to AEs | FOLFOX vs FOLFIRI | 2.53(1.88,1.66) | 0.99(0.01,101.76) | 0.51 |
|  | FULV vs FOLFIRI | 1.07(0.08,14.95) | 2.45(3.27,8.19) | 0.13 |
| Grade ≥3 any AEs | FOLFOX+Bevacizumab vs FOLFOX | 1.63(0.81,3.36) | 0.85(0.18,3.80) | 0.34 |
|  | FOLFOX+Cetuximab vs FOLFOX | 1.39(0.48,3.98) | 2.67(0.68,10.95) | 0.35 |
| Neutropenia | CAPOX vs CAPIRI | 0.20(0.03,0.95) | 0.20(0.07,0.62) | 0.98 |
|  | FOLFIRI vs CAPIRI | 1.62(0.61,4.32) | 2.09(0.30,12.22) | 0.81 |
| Febrile neutropenia | CAPOX+Bevacizumab vs CAPIRI+Bevacizumab | 0.23(0.01,2.52) | 0.04(0.01,0.26) | 0.32 |
|  | FOLFIRI+Bevacizumab vs CAPIRI+Bevacizumab | 0.33(0.10,1.0) | 2.02(0.08,32.74) | 0.30 |
| Anemia | CAPOX+Bevacizumab vs CAPIRI+Bevacizumab | 0.92(0.02,38.50) | 0.01(0,0.20) | 0.06 |
|  | FOLFIRI+Bevacizumab vs CAPIRI+Bevacizumab | 0.77(0.12,4.93) | 88.62(1.11,5517.7) | 0.05 |
| Thrombocytopenia | CAPOX+Bevacizumab vs CAPIRI+Bevacizumab | 4.07(98.96,2.26) | 1.25(0.01,83.75) | 0.01 |
|  | FOLFIRI+Bevacizumab vs CAPIRI+Bevacizumab | 0.36(0.01,6.72) | 5.33(16.87,1.02) | 0.01 |
| Diarrhea | CAPOX vs CAPIRI | 0.36(0.03,4.26) | 0.28(0.02,3.98) | 0.89 |
|  | FOLFIRI vs CAPIRI | 0.17(0.02,1.91) | 0.25(0.01,5.37) | 0.86 |
| Nausea | CAPOX vs CAPIRI | 0.58(0.06,5.47) | 0.05(0,0.54) | 0.13 |
|  | FOLFIRI vs CAPIRI | 0.42(0.09,1.94) | 5.29(0.19,136.18) | 0.15 |
| Vomiting | CAPOX+Bevacizumab vs CAPIRI+Bevacizumab | 0.91(0.22,4.02) | 0.91(0.04,66.78) | 1.00 |
|  | FOLFIRI+Bevacizumab vs CAPIRI+Bevacizumab | 0.46(0.13,1.41) | 0.56(0.01,11.17) | 0.90 |
| Mucositis/stomatitis | CAPOX vs CAPIRI | 0.39(0.01,9.33) | 0.29(0.01,13.67) | 0.92 |
|  | FOLFIRI vs CAPIRI | 0.99(0.06,16.73) | 1.35(0.01,84.10) | 0.91 |
| Peripheral sensory neuropathy | FOLFOX+Bevacizumab vs FOLFIRI+Bevacizumab | 76.91(2.35,7350.9) | 37.91(0.72,5876.1) | 0.75 |
|  | FOLFOXIRI+Bevacizumab vs FOLFIRI+Bevacizumab | 20.26(0.64,2285.4) | 46.11(0.55,8388.8) | 0.74 |
| Fatigue | FOLFOX+Bevacizumab vs FOLFOX | 0.73(0.11,4.76) | 1.17(0.14,5.31) | 0.66 |
|  | FOLFOX+Cetuximab vs FOLFOX | 1.41(0.44,4.65) | 6.53(0.35,261.08) | 0.31 |
| Hypertension | CAPOX+Bevacizumab vs CAPIRI+Bevacizumab | 1.20(0.43,3.46) | 3.38(0.65,18.68) | 0.28 |
|  | FOLFIRI+Bevacizumab vs CAPIRI+Bevacizumab | 1.09(0.47,2.69) | 0.41(0.07,2.29) | 0.30 |

Note: AEs,adverse events; CAPOX, capecitabine plus oxaliplatin; CAPIRI, capecitabine plus irinotecan; FOLFOX, 5-fluorouracil plus leucovorin plus oxaliplatin; FOLFIRI, 5-fluorouracil plus leucovorin plus irinotecan; FOLFOXIRI, 5-fluorouracil plus leucovorin plus oxaliplatin plus irinotecan.
