# Supplementary material for: Safety of first-line systemic therapy in patients with metastatic colorectal cancer: a network meta-analysis of randomized controlled trials
Source: BMC Cancer. 2024 Jul 24;24:893. doi: 10.1186/s12885-024-12662-3 (PMC11270896; doi:10.1186/s12885-024-12662-3)
Supplement: Supplementary file 2 — Supplementary Material 2 [file 12885_2024_12662_MOESM2_ESM.docx]

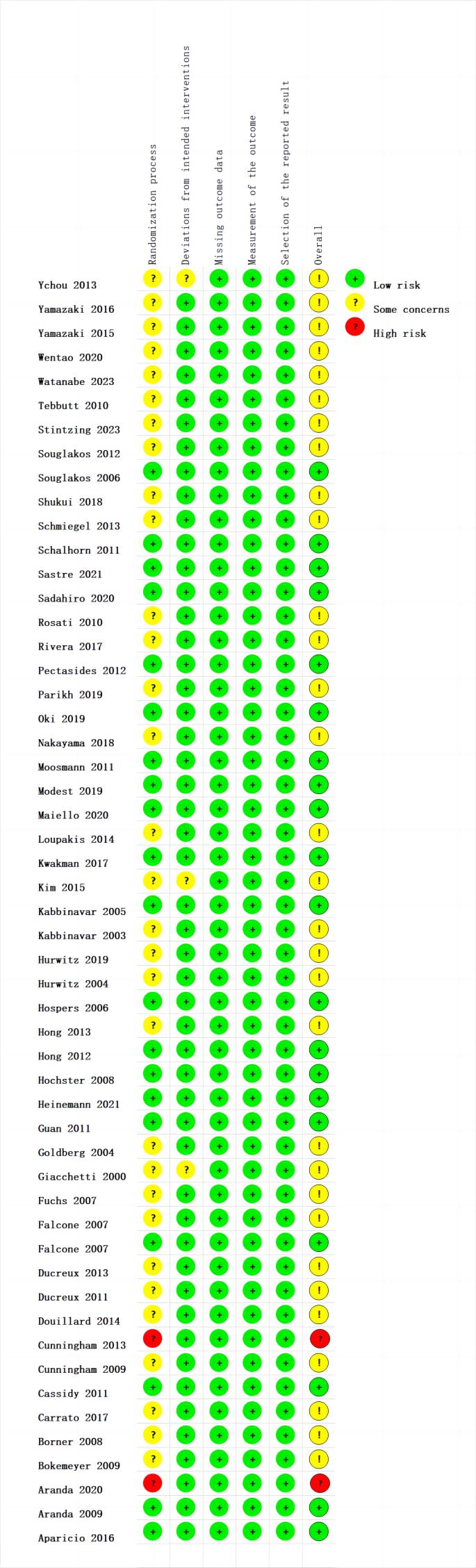


**Supplementary Figure S1 Bias risk for each included study.**


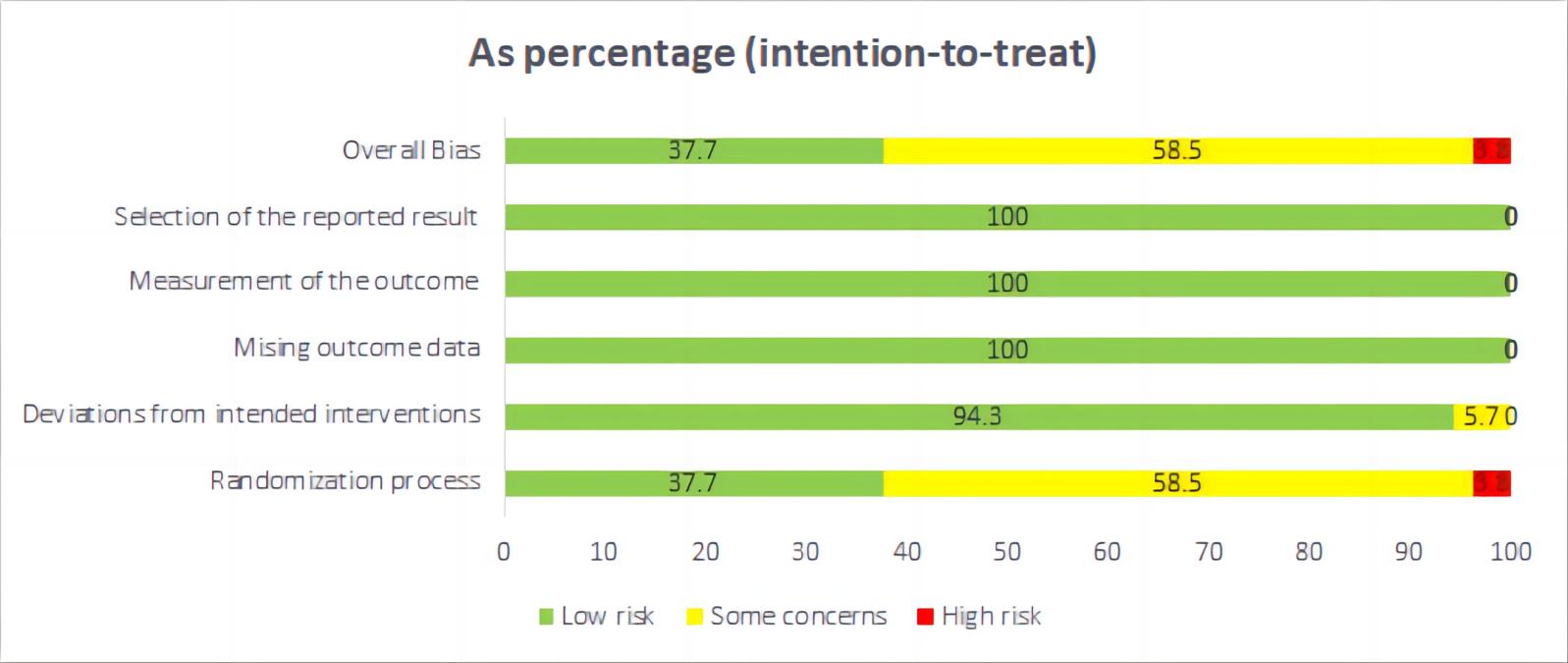


**Supplementary Figure S2 Bias risk map for all included studies.**


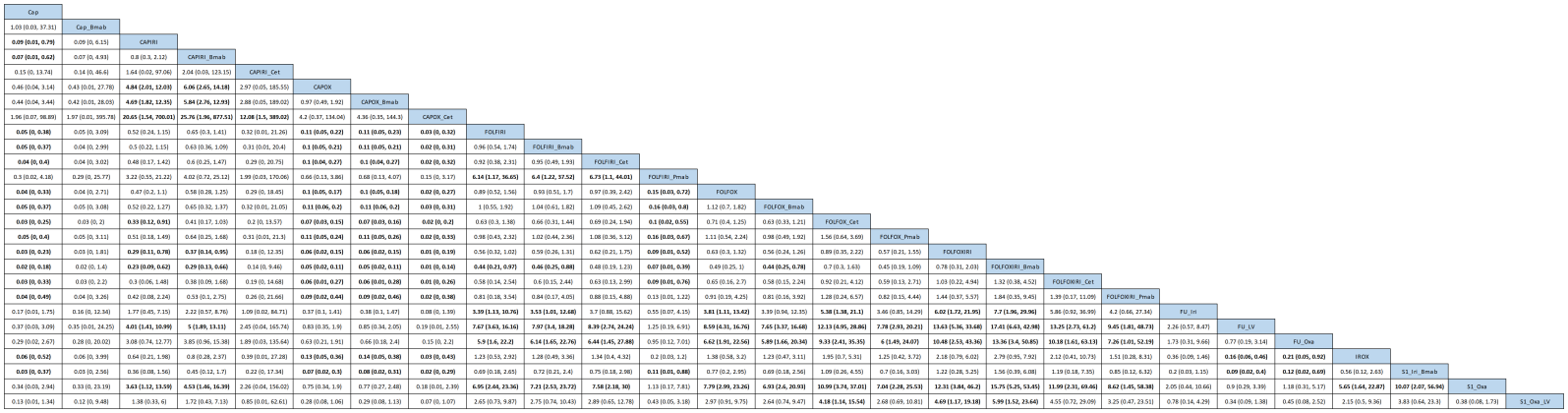


**Supplementary Figure S3 Risk ratios (95%CIs) of Neutropenia.**

Bmab, bevacizumab; Cet, cetuximab; Cap, capecitabine; Oxa, oxaliplatin; Pmab, panitumumab;FU,5-fluorouracil; LV,leucovorin; Iri,irinotecan; CAPOX, capecitabine plus oxaliplatin; CAPIRI, capecitabine plus irinotecan; FOLFOX, 5-fluorouracil plus leucovorin plus oxaliplatin; FOLFOXIRI, 5-fluorouracil plus leucovorin plus oxaliplatin plus irinotecan; FOLFIRI, 5-fluorouracil plus leucovorin plus irinotecan; FUIRI, 5-fluorouracil plus irinotecan; IROX, irinotecan plus oxaliplatin. Significant results are presented in bold.


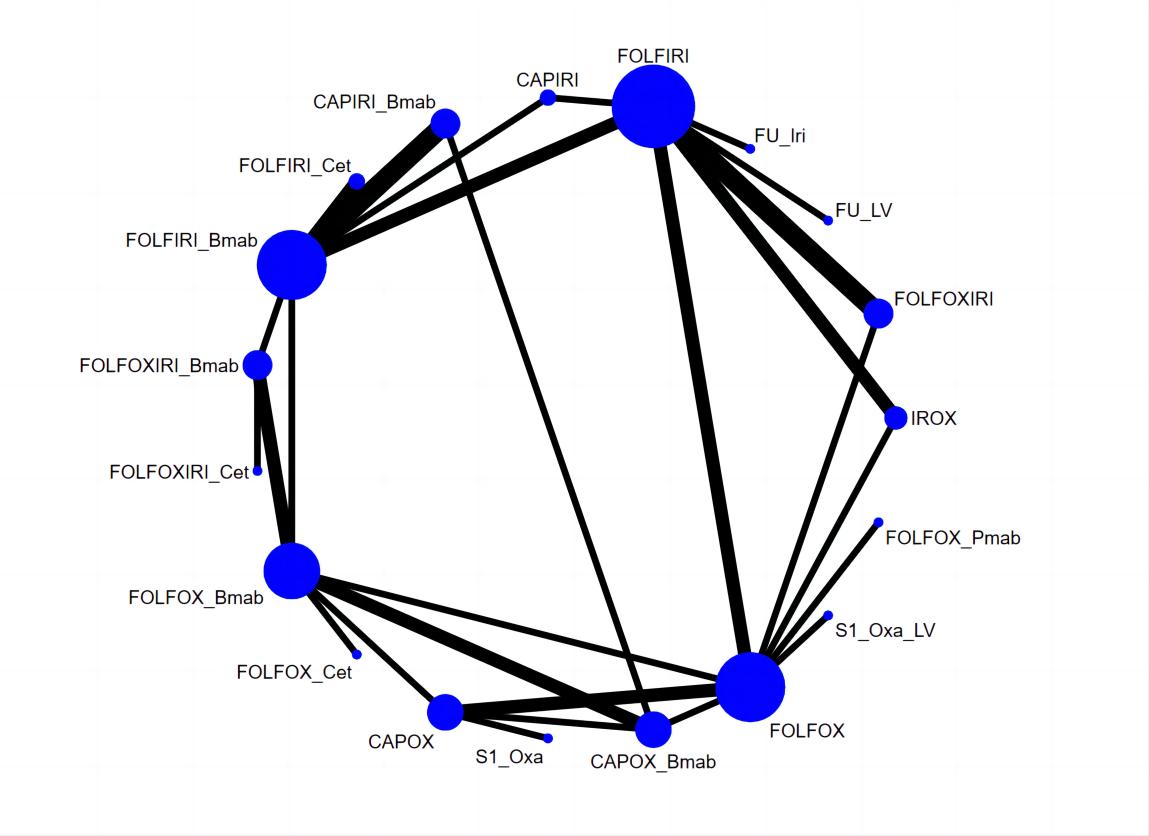


**Supplementary Figure S4A Network plot of Febrile neutropenia.**

**
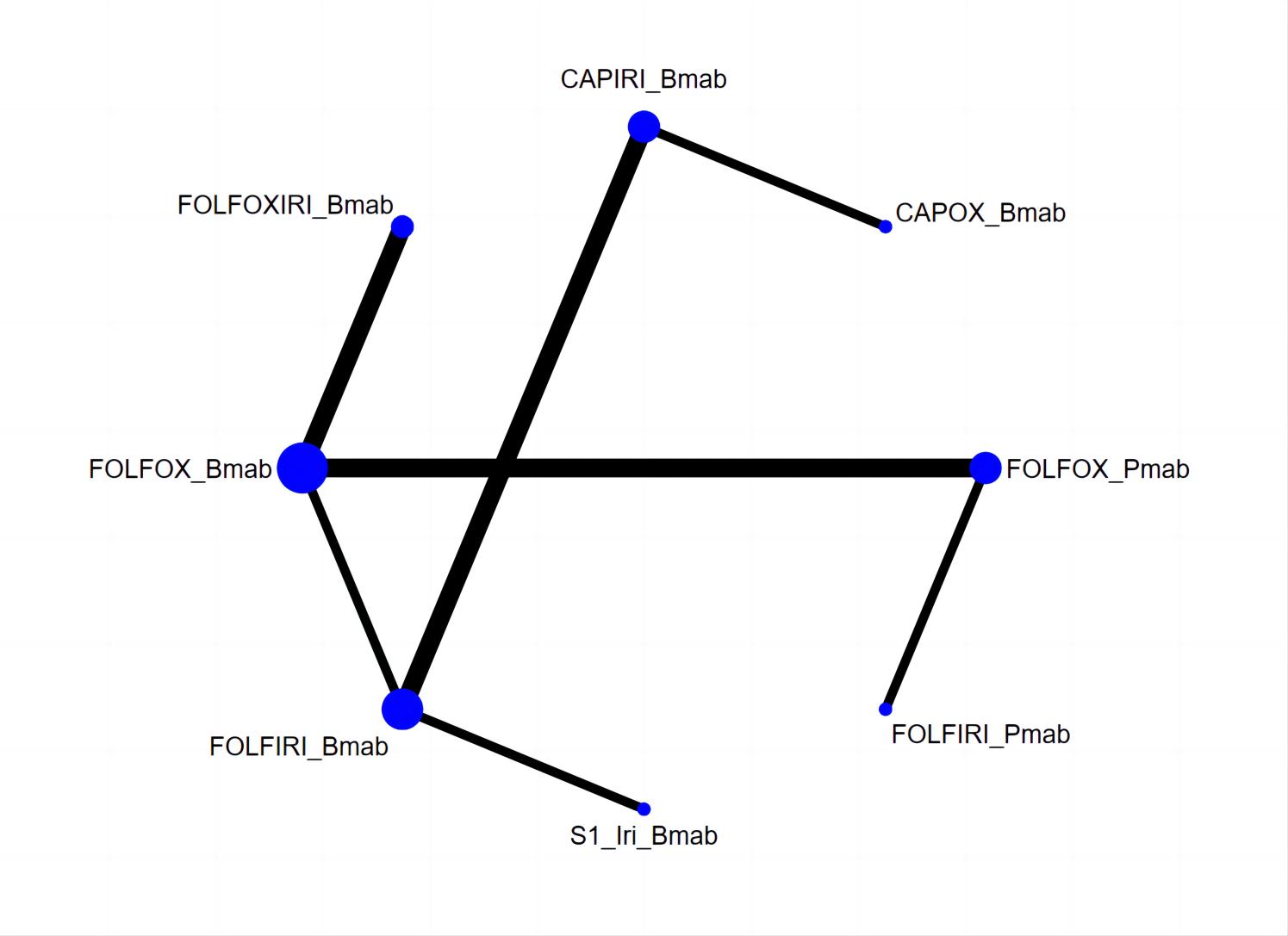
**

**Supplementary Figure S4B Network plot of Anorexia.**

**Supplementary Figure S4 Network plot of Adverse Events. (A) Febrile neutropenia; (B) Anorexia.**

Bmab, bevacizumab; Cap, capecitabine; Cet, cetuximab; Oxa, oxaliplatin; Pmab, panitumumab;FU,5-fluorouracil; LV,leucovorin; Iri,irinotecan; CAPOX, capecitabine plus oxaliplatin; CAPIRI, capecitabine plus irinotecan; FOLFOX, 5-fluorouracil plus leucovorin plus oxaliplatin; FOLFOXIRI, 5-fluorouracil plus leucovorin plus oxaliplatin plus irinotecan; FOLFIRI, 5-fluorouracil plus leucovorin plus irinotecan; FUIRI, 5-fluorouracil plus irinotecan; IROX, irinotecan plus oxaliplatin. Each node represented a different treatment and its size depended on the number of patients that is directly examined. The nodes were joined by lines with different thickness which shows whether there was a direct relationship between treatments and the thickness was weighted according to the available direct evidence between them.


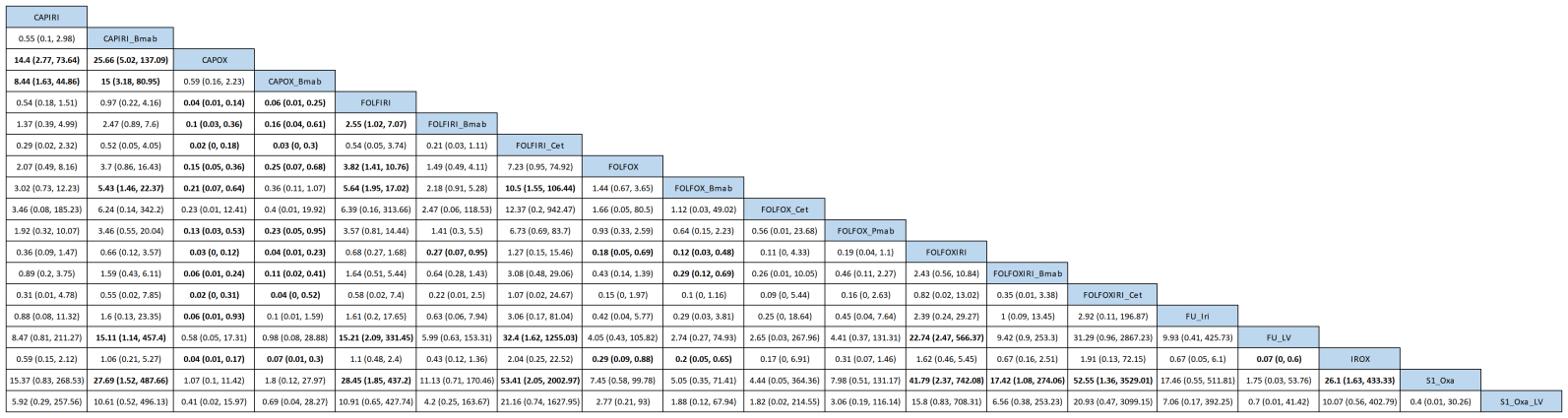


**Supplementary Figure S5 Risk ratios (95%CIs) of Febrile neutropenia.**

Bmab, bevacizumab; Cap, capecitabine; Cet, cetuximab; Oxa, oxaliplatin; Pmab, panitumumab;FU,5-fluorouracil; LV,leucovorin; Iri,irinotecan; CAPOX, capecitabine plus oxaliplatin; CAPIRI, capecitabine plus irinotecan; FOLFOX, 5-fluorouracil plus leucovorin plus oxaliplatin; FOLFOXIRI, 5-fluorouracil plus leucovorin plus oxaliplatin plus irinotecan; FOLFIRI, 5-fluorouracil plus leucovorin plus irinotecan; FUIRI, 5-fluorouracil plus irinotecan; IROX, irinotecan plus oxaliplatin. Significant results are presented in bold.


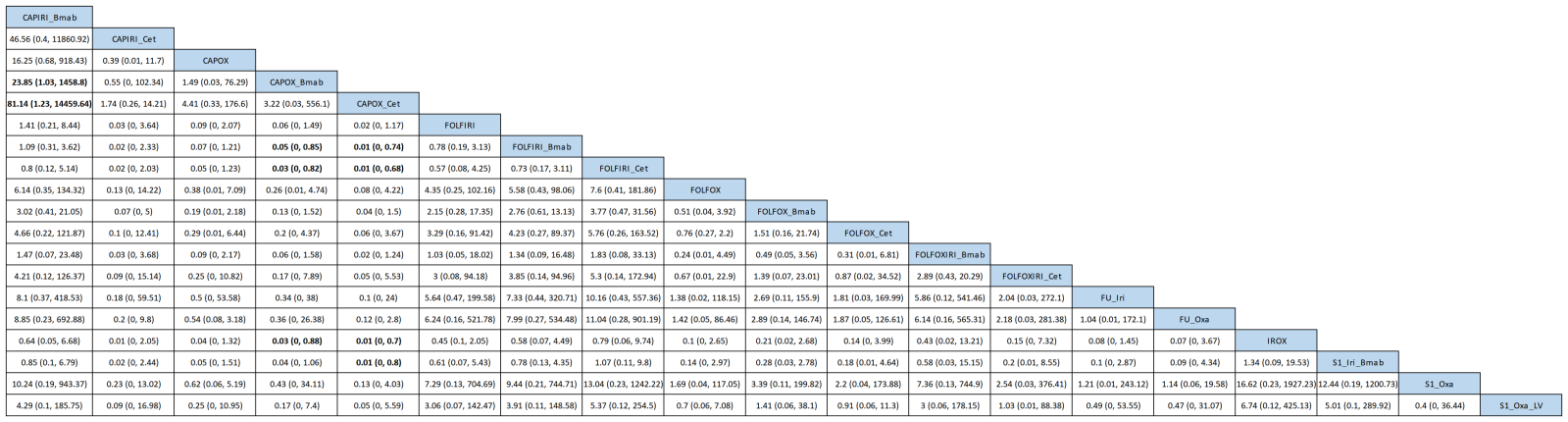


**Supplementary Figure S6 Risk ratios (95%CIs) of Leukopenia.**

Bmab, bevacizumab; Cap, capecitabine; Cet, cetuximab; Oxa, oxaliplatin; Pmab, panitumumab;FU,5-fluorouracil; LV,leucovorin; Iri,irinotecan; CAPOX, capecitabine plus oxaliplatin; CAPIRI, capecitabine plus irinotecan; FOLFOX, 5-fluorouracil plus leucovorin plus oxaliplatin; FOLFOXIRI, 5-fluorouracil plus leucovorin plus oxaliplatin plus irinotecan; FOLFIRI, 5-fluorouracil plus leucovorin plus irinotecan; FUIRI, 5-fluorouracil plus irinotecan; IROX, irinotecan plus oxaliplatin. Significant results are presented in bold.


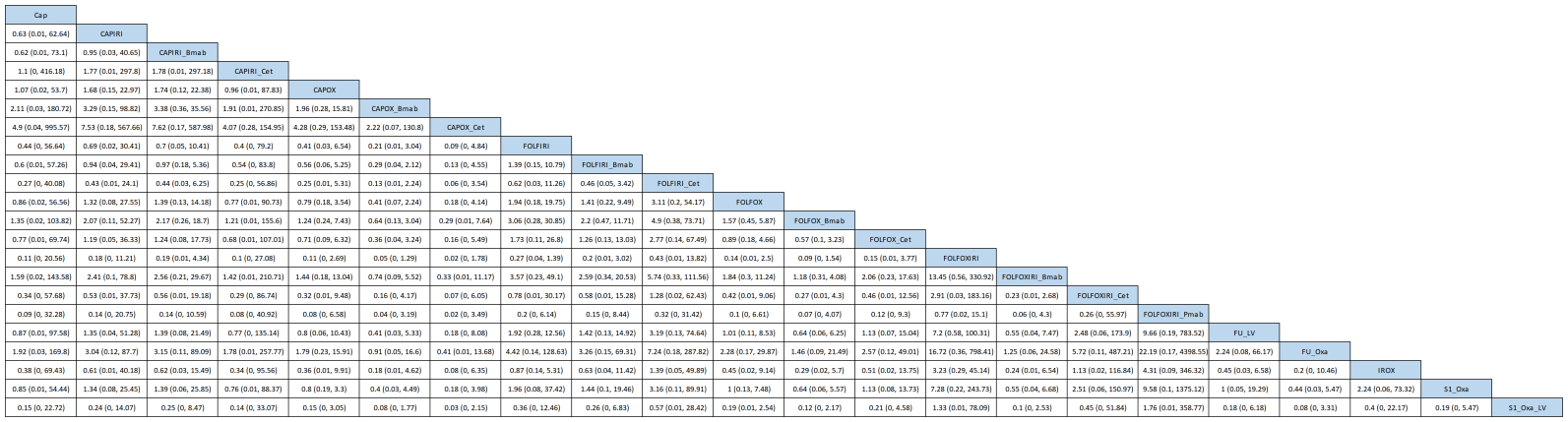


**Supplementary Figure S7 Risk ratios (95%CIs) of Anemia.**

Bmab, bevacizumab; Cap, capecitabine; Cet, cetuximab; Oxa, oxaliplatin; Pmab, panitumumab;FU,5-fluorouracil; LV,leucovorin; Iri,irinotecan; CAPOX, capecitabine plus oxaliplatin; CAPIRI, capecitabine plus irinotecan; FOLFOX, 5-fluorouracil plus leucovorin plus oxaliplatin; FOLFOXIRI, 5-fluorouracil plus leucovorin plus oxaliplatin plus irinotecan; FOLFIRI, 5-fluorouracil plus leucovorin plus irinotecan; FUIRI, 5-fluorouracil plus irinotecan; IROX, irinotecan plus oxaliplatin. Significant results are presented in bold.


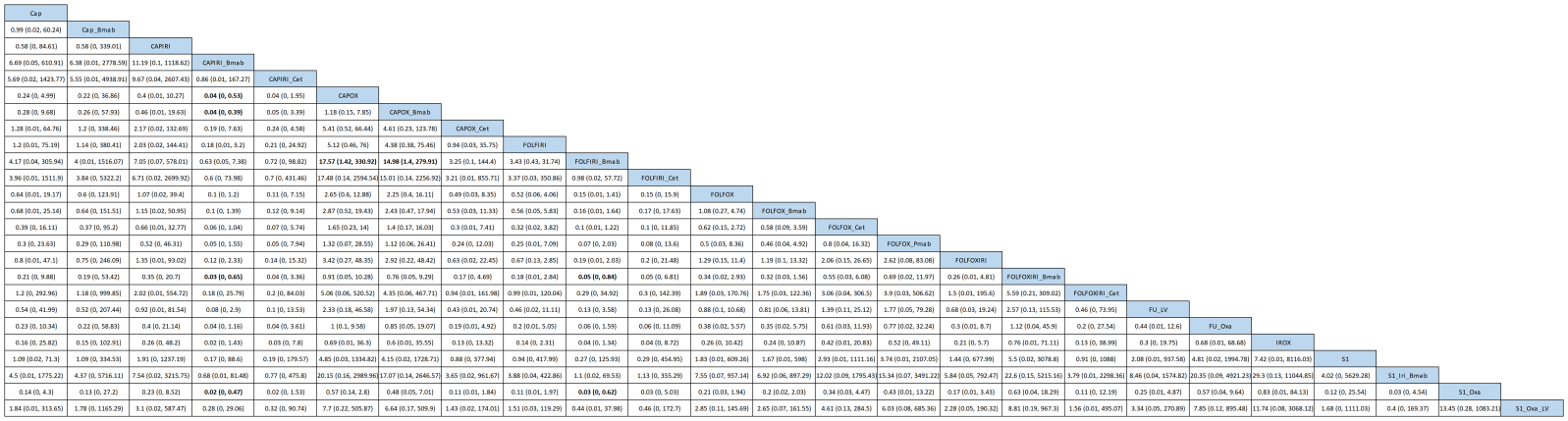


**Supplementary Figure S8 Risk ratios (95%CIs) of Thrombocytopenia.**

Bmab, bevacizumab; Cap, capecitabine; Cet, cetuximab; Oxa, oxaliplatin; Pmab, panitumumab;FU,5-fluorouracil; LV,leucovorin; Iri,irinotecan; CAPOX, capecitabine plus oxaliplatin; CAPIRI, capecitabine plus irinotecan; FOLFOX, 5-fluorouracil plus leucovorin plus oxaliplatin; FOLFOXIRI, 5-fluorouracil plus leucovorin plus oxaliplatin plus irinotecan; FOLFIRI, 5-fluorouracil plus leucovorin plus irinotecan; FUIRI, 5-fluorouracil plus irinotecan; IROX, irinotecan plus oxaliplatin. Significant results are presented in bold.


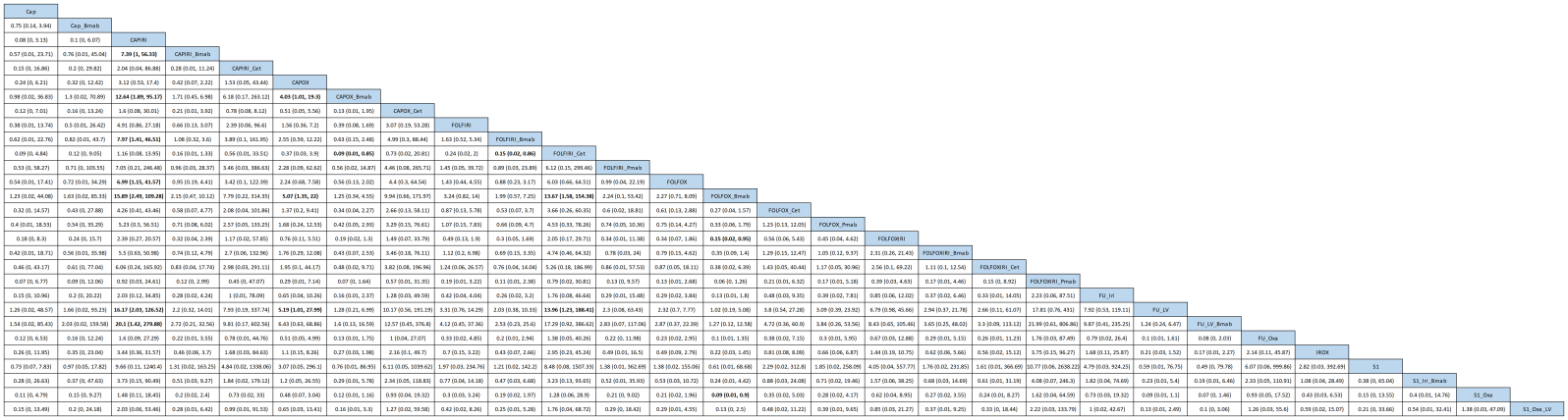


**Supplementary Figure S9 Risk ratios (95%CIs) of Diarrhea.**

Bmab, bevacizumab; Cap, capecitabine; Cet, cetuximab; Oxa, oxaliplatin; Pmab, panitumumab;FU,5-fluorouracil; LV,leucovorin; Iri,irinotecan; CAPOX, capecitabine plus oxaliplatin; CAPIRI, capecitabine plus irinotecan; FOLFOX, 5-fluorouracil plus leucovorin plus oxaliplatin; FOLFOXIRI, 5-fluorouracil plus leucovorin plus oxaliplatin plus irinotecan; FOLFIRI, 5-fluorouracil plus leucovorin plus irinotecan; FUIRI, 5-fluorouracil plus irinotecan; IROX, irinotecan plus oxaliplatin. Significant results are presented in bold.


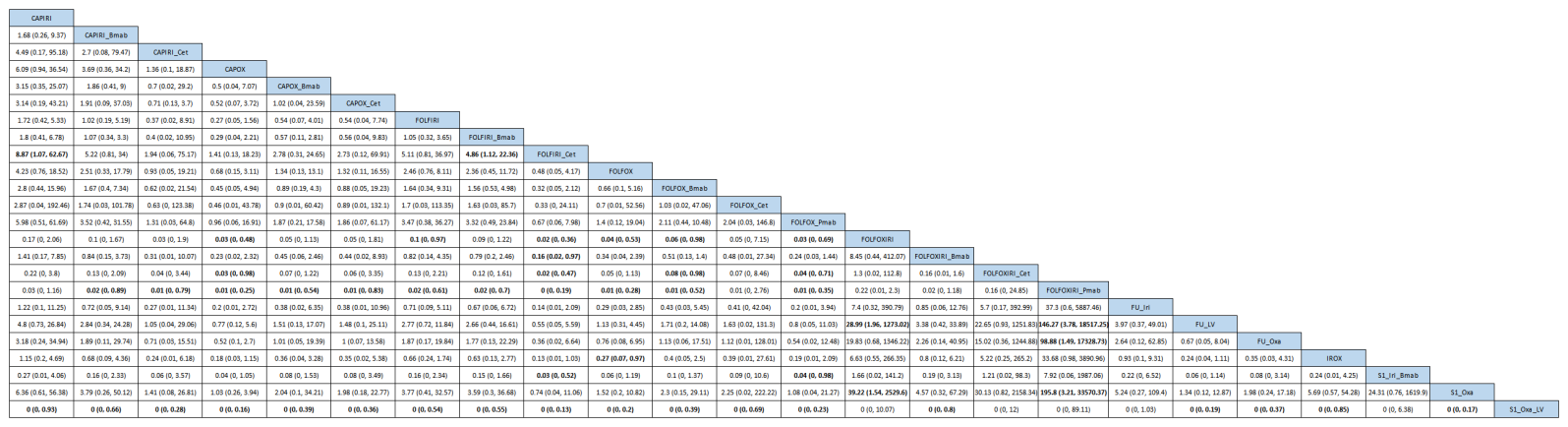


**Supplementary Figure S10 Risk ratios (95%CIs) of Nausea.**

Bmab, bevacizumab; Cap, capecitabine; Cet, cetuximab; Oxa, oxaliplatin; Pmab, panitumumab;FU,5-fluorouracil; LV,leucovorin; Iri,irinotecan; CAPOX, capecitabine plus oxaliplatin; CAPIRI, capecitabine plus irinotecan; FOLFOX, 5-fluorouracil plus leucovorin plus oxaliplatin; FOLFOXIRI, 5-fluorouracil plus leucovorin plus oxaliplatin plus irinotecan; FOLFIRI, 5-fluorouracil plus leucovorin plus irinotecan; FUIRI, 5-fluorouracil plus irinotecan; IROX, irinotecan plus oxaliplatin. Significant results are presented in bold.


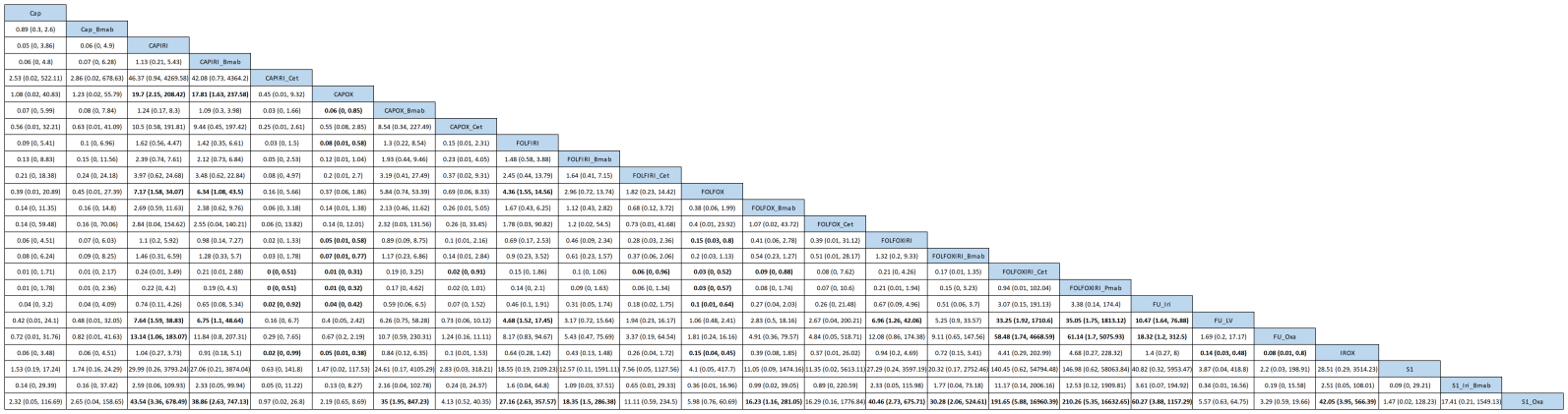


**Supplementary Figure S11 Risk ratios (95%CIs) of Vomiting.**

Bmab, bevacizumab; Cap, capecitabine; Cet, cetuximab; Oxa, oxaliplatin; Pmab, panitumumab;FU,5-fluorouracil; LV,leucovorin; Iri,irinotecan; CAPOX, capecitabine plus oxaliplatin; CAPIRI, capecitabine plus irinotecan; FOLFOX, 5-fluorouracil plus leucovorin plus oxaliplatin; FOLFOXIRI, 5-fluorouracil plus leucovorin plus oxaliplatin plus irinotecan; FOLFIRI, 5-fluorouracil plus leucovorin plus irinotecan; FUIRI, 5-fluorouracil plus irinotecan; IROX, irinotecan plus oxaliplatin. Significant results are presented in bold.


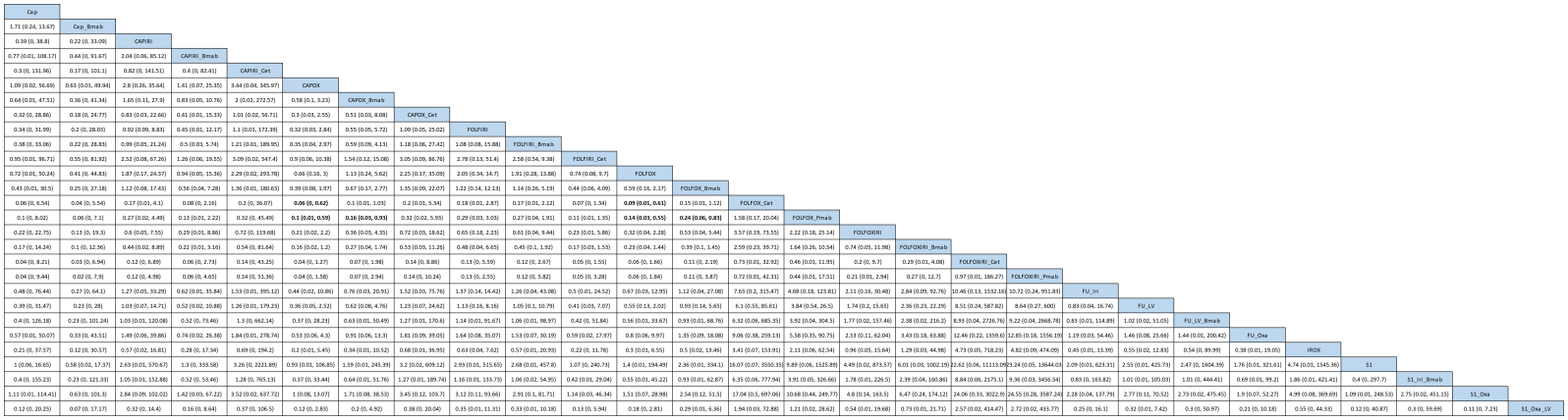


**Supplementary Figure S12 Risk ratios (95%CIs) of Mucositis/stomatitis**. Bmab, bevacizumab; Cap, capecitabine; Cet, cetuximab; Oxa, oxaliplatin; Pmab, panitumumab;FU,5-fluorouracil; LV,leucovorin; Iri,irinotecan; CAPOX, capecitabine plus oxaliplatin; CAPIRI, capecitabine plus irinotecan; FOLFOX, 5-fluorouracil plus leucovorin plus oxaliplatin; FOLFOXIRI, 5-fluorouracil plus leucovorin plus oxaliplatin plus irinotecan; FOLFIRI, 5-fluorouracil plus leucovorin plus irinotecan; FUIRI, 5-fluorouracil plus irinotecan; IROX, irinotecan plus oxaliplatin. Significant results are presented in bold.


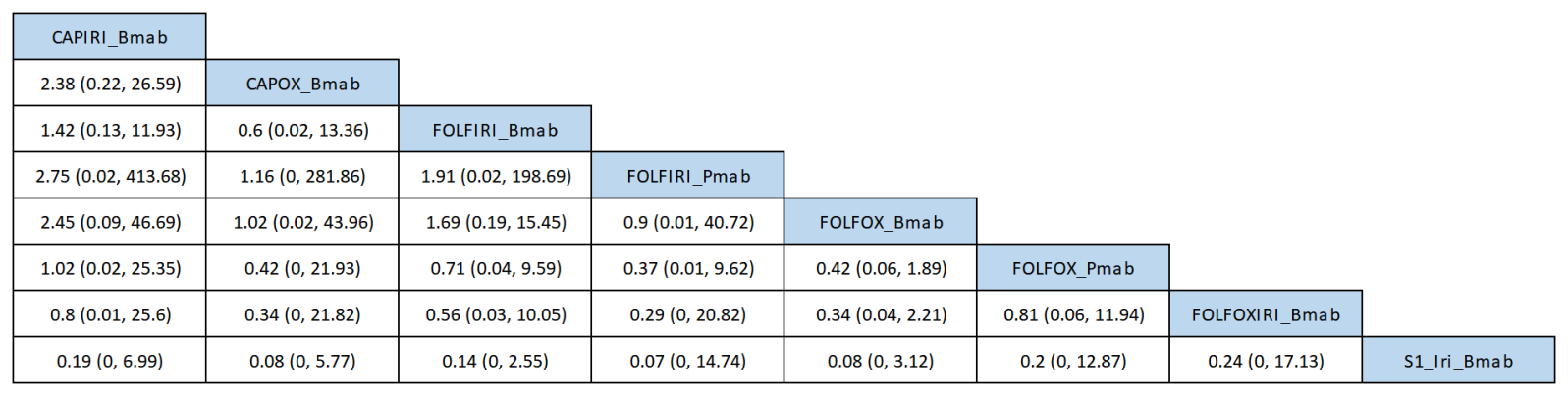


**Supplementary Figure S13 Risk ratios (95%CIs) of Anorexia.**

Bmab, bevacizumab; Cap, capecitabine; Cet, cetuximab; Oxa, oxaliplatin; Pmab, panitumumab;FU,5-fluorouracil; LV,leucovorin; Iri,irinotecan; CAPOX, capecitabine plus oxaliplatin; CAPIRI, capecitabine plus irinotecan; FOLFOX, 5-fluorouracil plus leucovorin plus oxaliplatin; FOLFOXIRI, 5-fluorouracil plus leucovorin plus oxaliplatin plus irinotecan; FOLFIRI, 5-fluorouracil plus leucovorin plus irinotecan; FUIRI, 5-fluorouracil plus irinotecan; IROX, irinotecan plus oxaliplatin. Significant results are presented in bold.


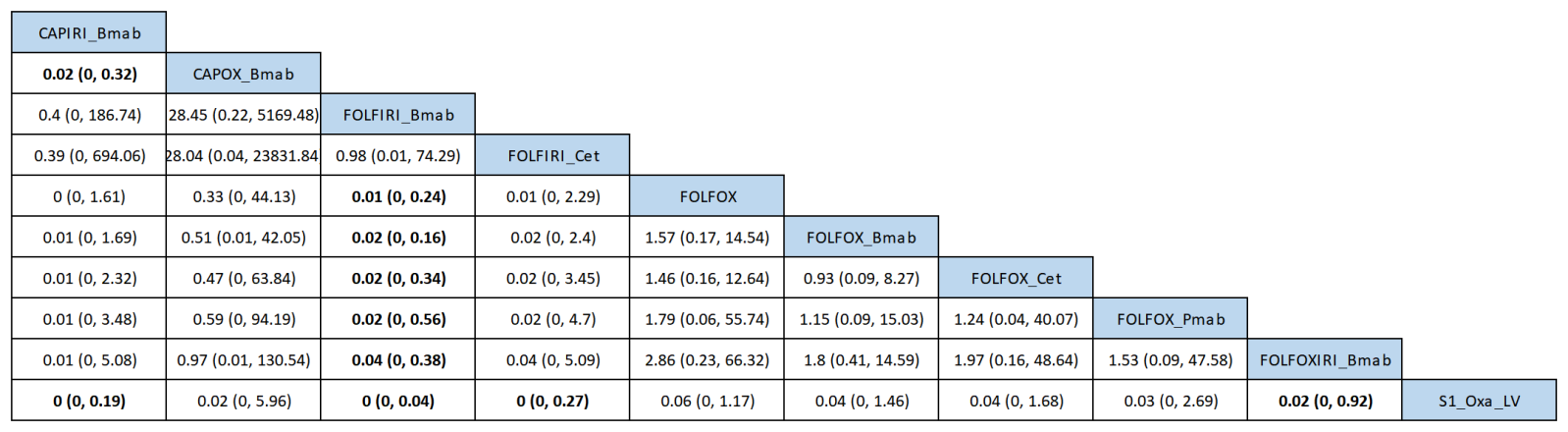


**Supplementary Figure S14 Risk ratios (95%CIs) of Peripheral sensory neuropathy.**

Bmab, bevacizumab; Cap, capecitabine; Cet, cetuximab; Oxa, oxaliplatin; Pmab, panitumumab;FU,5-fluorouracil; LV,leucovorin; Iri,irinotecan; CAPOX, capecitabine plus oxaliplatin; CAPIRI, capecitabine plus irinotecan; FOLFOX, 5-fluorouracil plus leucovorin plus oxaliplatin; FOLFOXIRI, 5-fluorouracil plus leucovorin plus oxaliplatin plus irinotecan; FOLFIRI, 5-fluorouracil plus leucovorin plus irinotecan; FUIRI, 5-fluorouracil plus irinotecan; IROX, irinotecan plus oxaliplatin. Significant results are presented in bold.


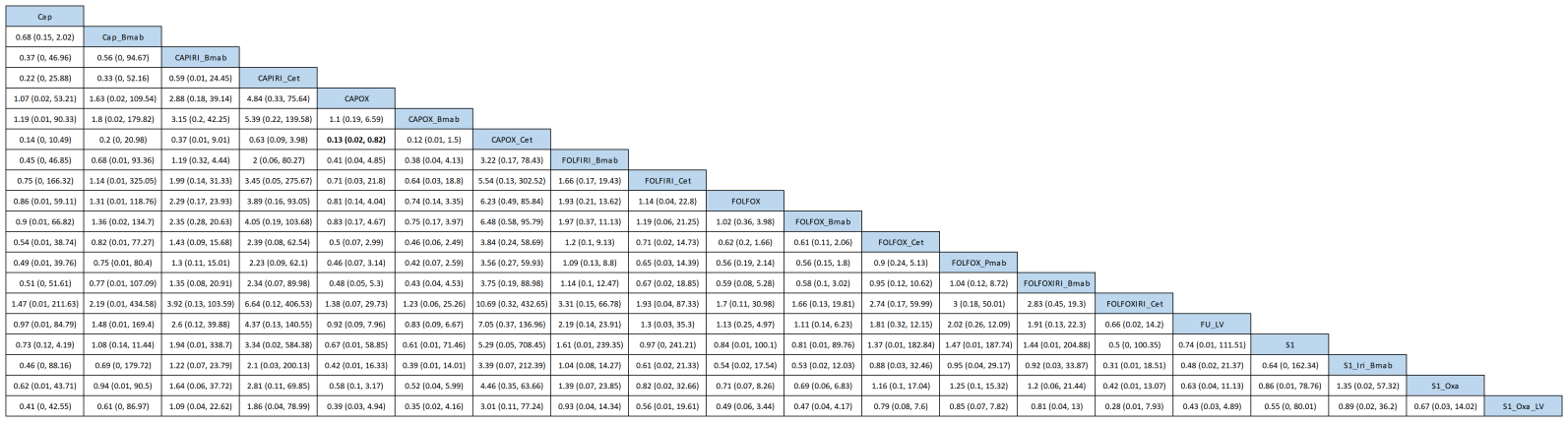


**Supplementary Figure S15 Risk ratios (95%CIs) of Fatigue.**

Bmab, bevacizumab; Cap, capecitabine; Cet, cetuximab; Oxa, oxaliplatin; Pmab, panitumumab;FU,5-fluorouracil; LV,leucovorin; Iri,irinotecan; CAPOX, capecitabine plus oxaliplatin; CAPIRI, capecitabine plus irinotecan; FOLFOX, 5-fluorouracil plus leucovorin plus oxaliplatin; FOLFOXIRI, 5-fluorouracil plus leucovorin plus oxaliplatin plus irinotecan; FOLFIRI, 5-fluorouracil plus leucovorin plus irinotecan; FUIRI, 5-fluorouracil plus irinotecan; IROX, irinotecan plus oxaliplatin. Significant results are presented in bold.


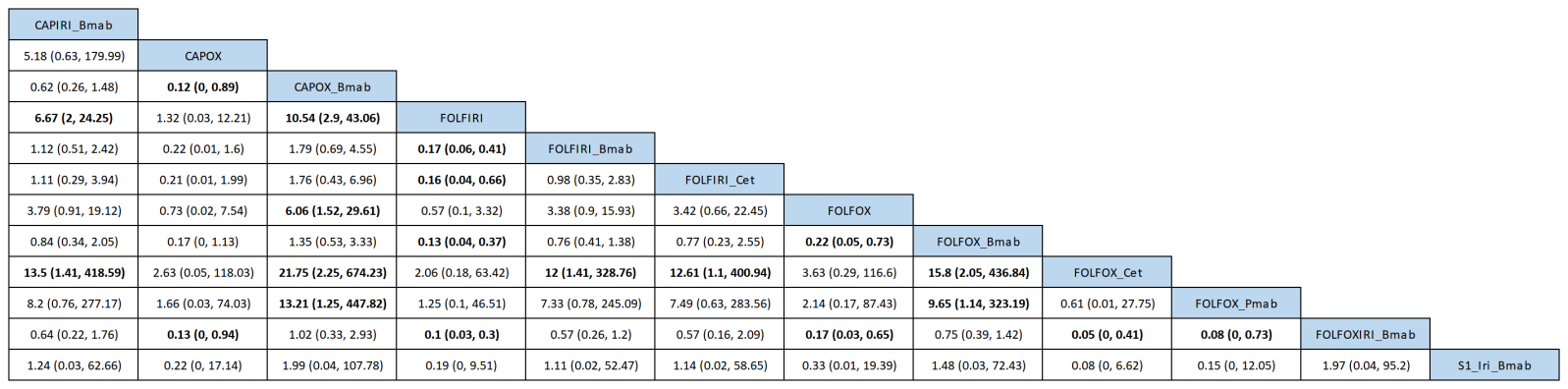


**Supplementary Figure S16 Risk ratios (95%CIs) of Hypertension.**

Bmab, bevacizumab; Cap, capecitabine; Cet, cetuximab; Oxa, oxaliplatin; Pmab, panitumumab;FU,5-fluorouracil; LV,leucovorin; Iri,irinotecan; CAPOX, capecitabine plus oxaliplatin; CAPIRI, capecitabine plus irinotecan; FOLFOX, 5-fluorouracil plus leucovorin plus oxaliplatin; FOLFOXIRI, 5-fluorouracil plus leucovorin plus oxaliplatin plus irinotecan; FOLFIRI, 5-fluorouracil plus leucovorin plus irinotecan; FUIRI, 5-fluorouracil plus irinotecan; IROX, irinotecan plus oxaliplatin. Significant results are presented in bold.


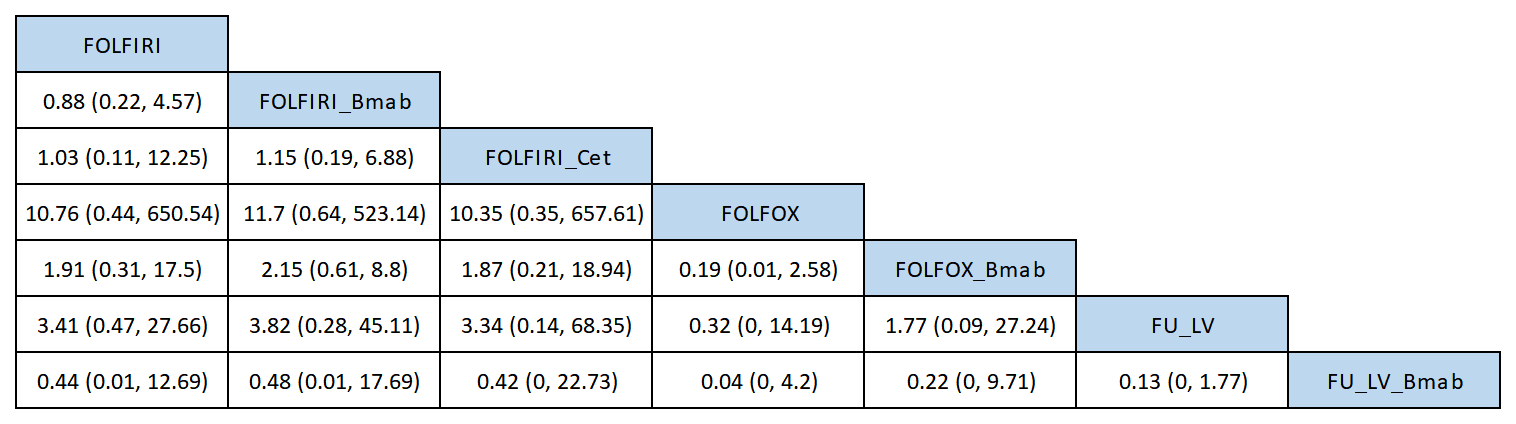


**Supplementary Figure S17 Risk ratios (95%CIs) of Thromboembolic events.**

Bmab, bevacizumab; Cap, capecitabine; Cet, cetuximab; Oxa, oxaliplatin; Pmab, panitumumab;FU,5-fluorouracil; LV,leucovorin; Iri,irinotecan; CAPOX, capecitabine plus oxaliplatin; CAPIRI, capecitabine plus irinotecan; FOLFOX, 5-fluorouracil plus leucovorin plus oxaliplatin; FOLFOXIRI, 5-fluorouracil plus leucovorin plus oxaliplatin plus irinotecan; FOLFIRI, 5-fluorouracil plus leucovorin plus irinotecan; FUIRI, 5-fluorouracil plus irinotecan; IROX, irinotecan plus oxaliplatin. Significant results are presented in bold.


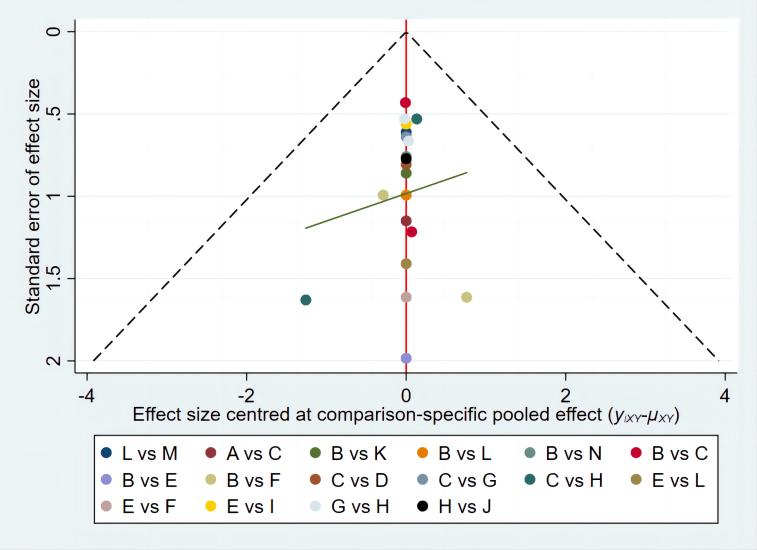


**Supplementary Figure S18A** Funnel plot for the network of Death related to adverse events;

**
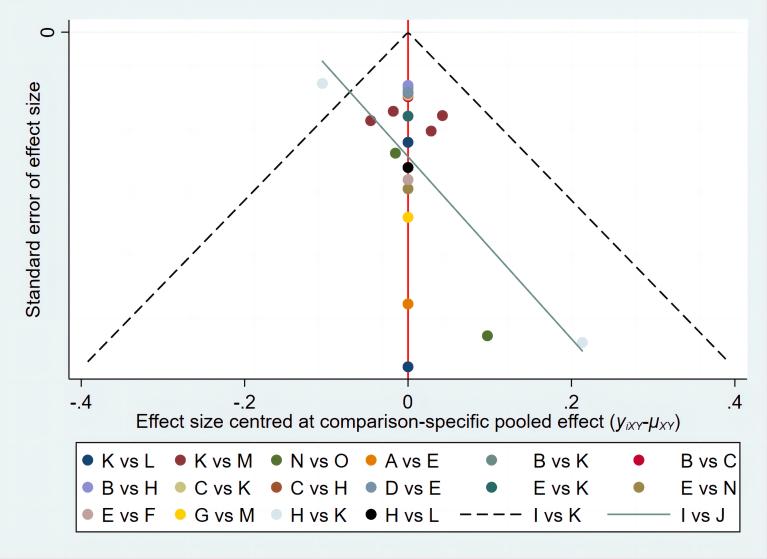
**

**Supplementary Figure S18B** Funnel plot for the network of Grade ≥3 any Adverse Events;


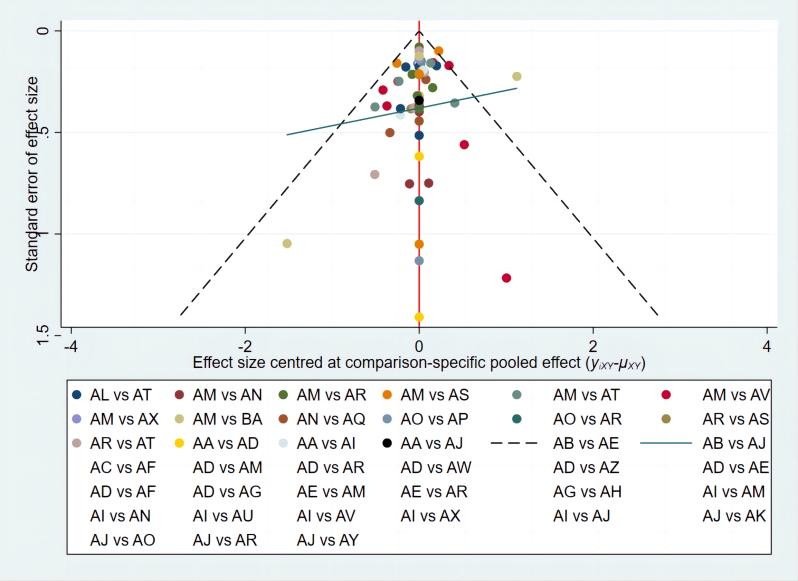


**Supplementary Figure S18C** Funnel plot for the network of Neutropenia;


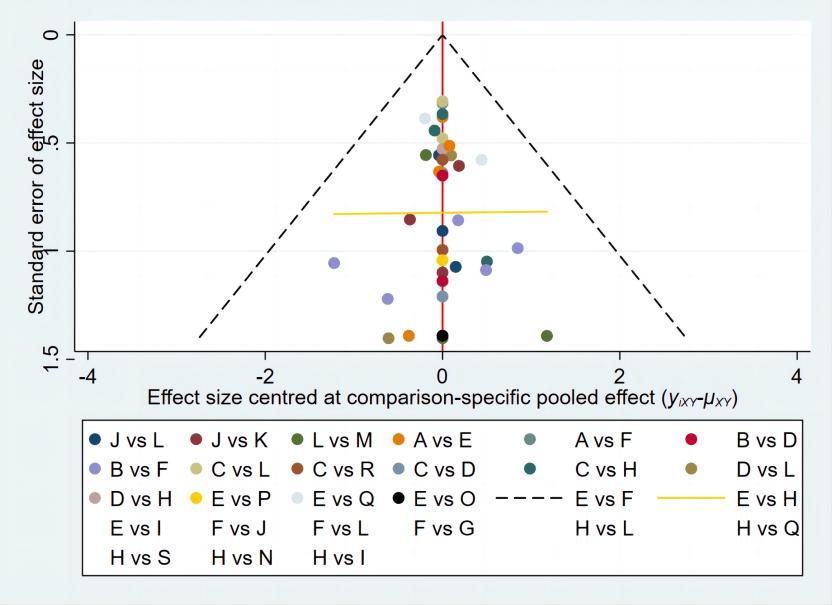


**Supplementary Figure S18D** Funnel plot for the network of Febrile neutropenia;


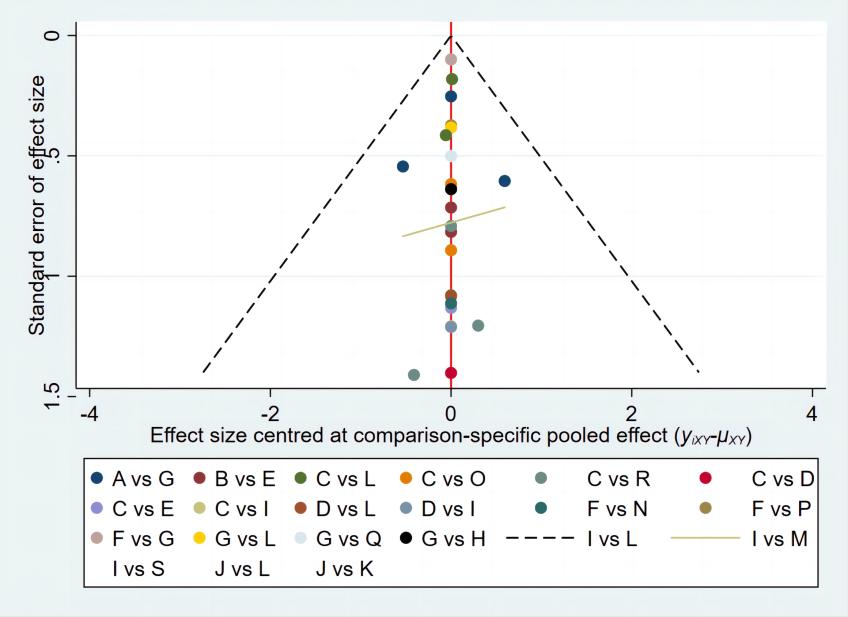


**Supplementary Figure S18E** Funnel plot for the network of Leukopenia;


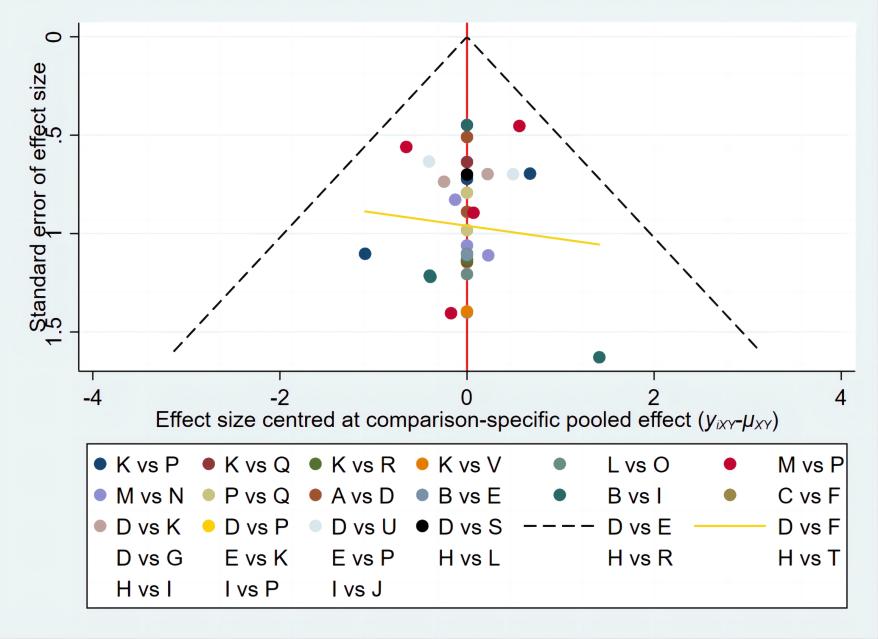


**Supplementary Figure S18F** Funnel plot for the network of Anemia;


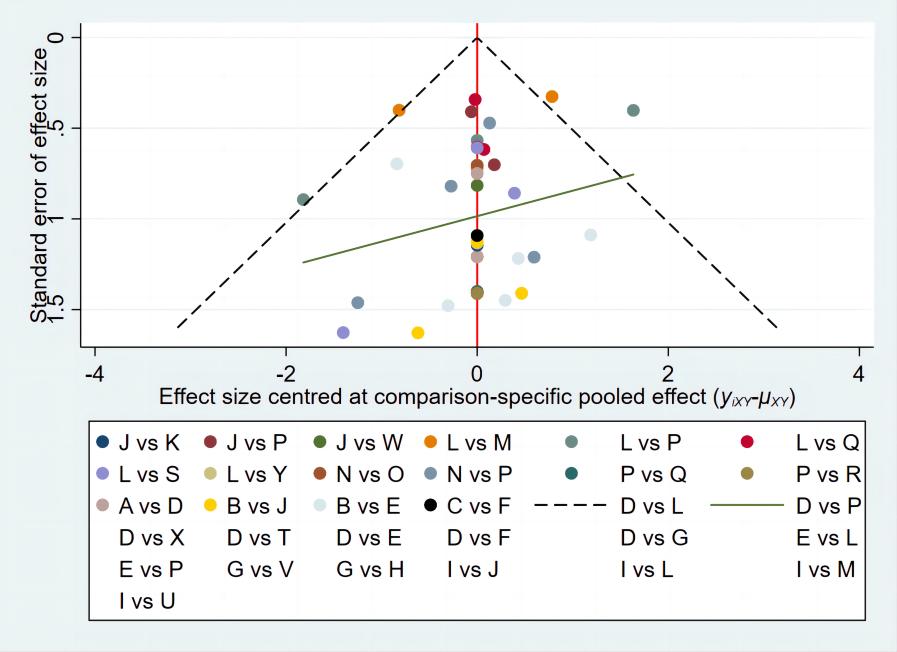


**Supplementary Figure S18G** Funnel plot for the network of Thrombocytopenia;


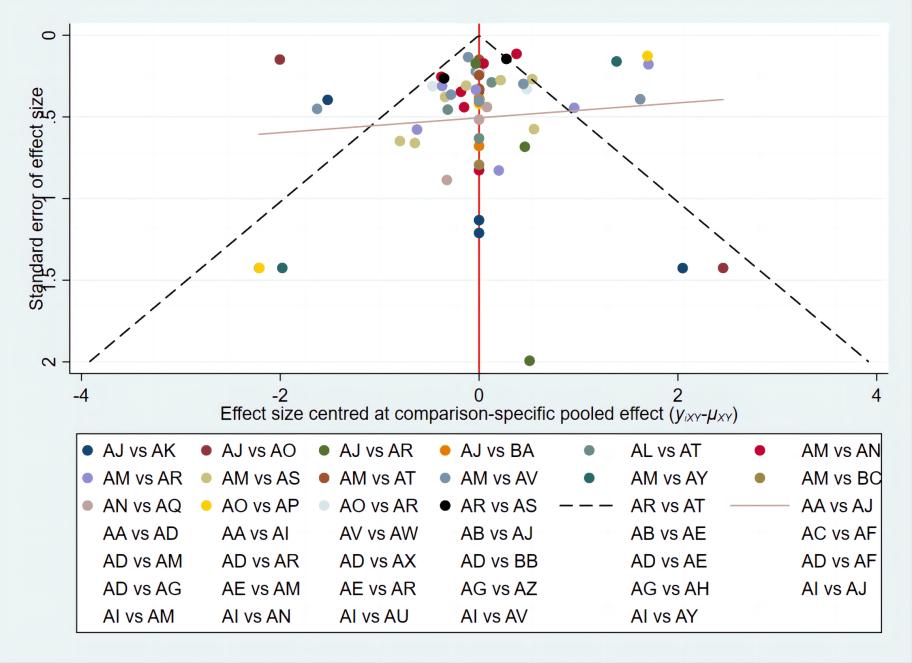


**Supplementary Figure S18H** Funnel plot for the network of Diarrhea;

**Supplementary Figure S18 Funnel plot.** Funnel plot for the network of (A) Death related to adverse events; (B) Grade ≥3 any Adverse Events; (C) Neutropenia; (D) Febrile neutropenia; (E) Leukopenia; (F) Anemia; (G) Thrombocytopenia; (H) Diarrhea.


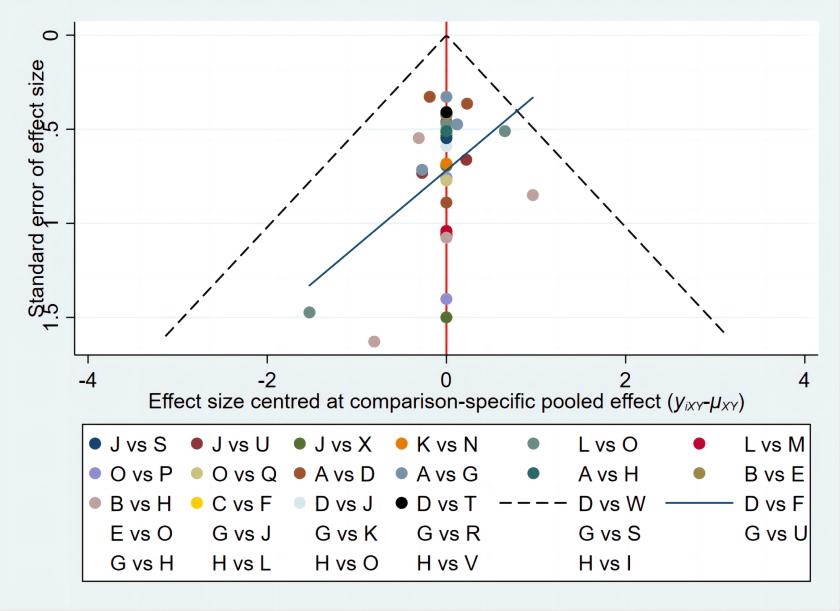


**Supplementary Figure S19A** Funnel plot for the network of Nausea;


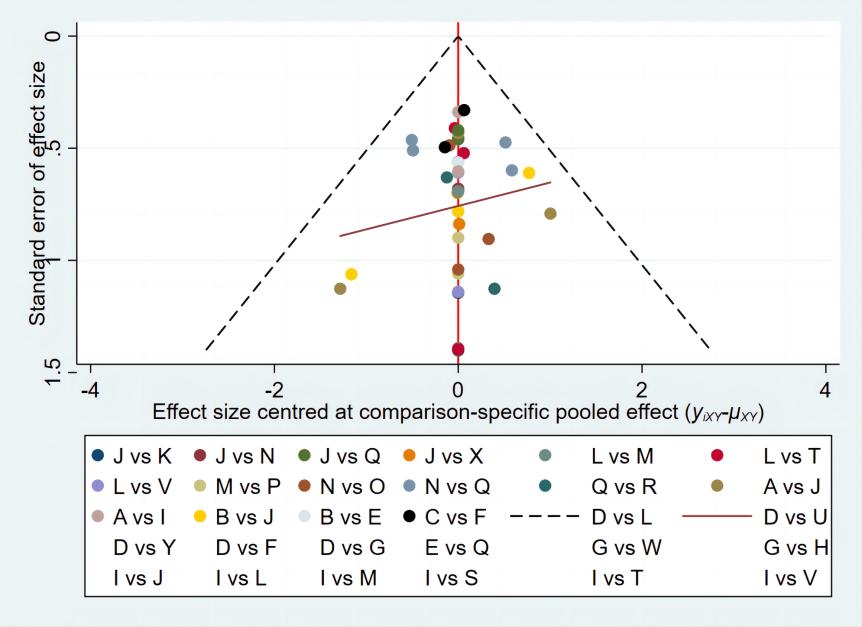


**Supplementary Figure S19B** Funnel plot for the network of Vomiting;


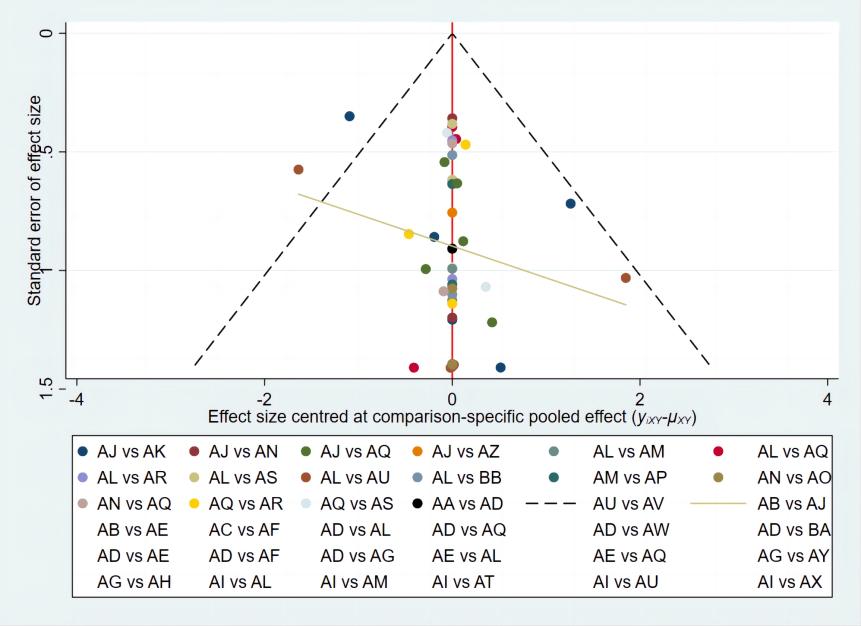


**Supplementary Figure S19C** Funnel plot for the network of Mucositis/stomatitis;


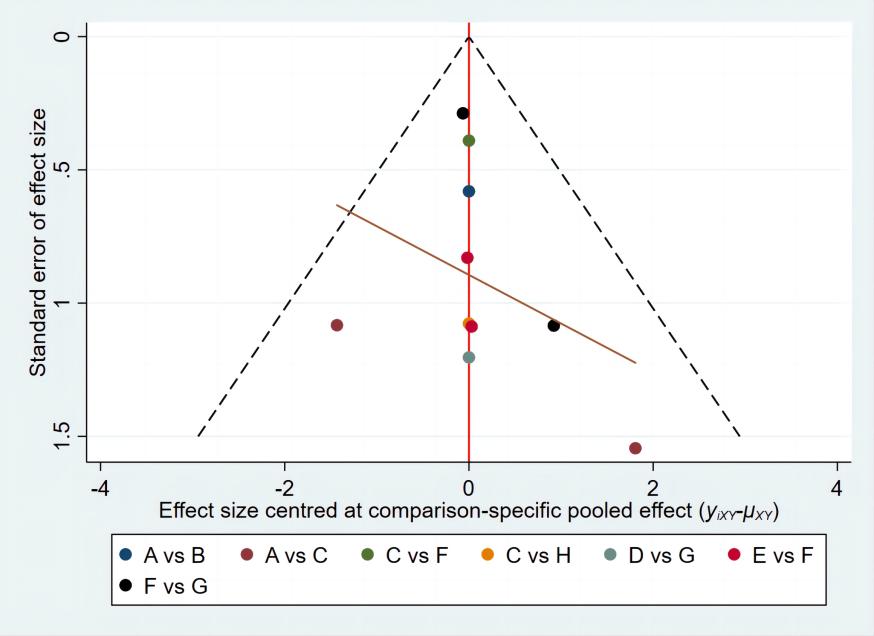


**Supplementary Figure S19D** Funnel plot for the network of Anorexia;

**
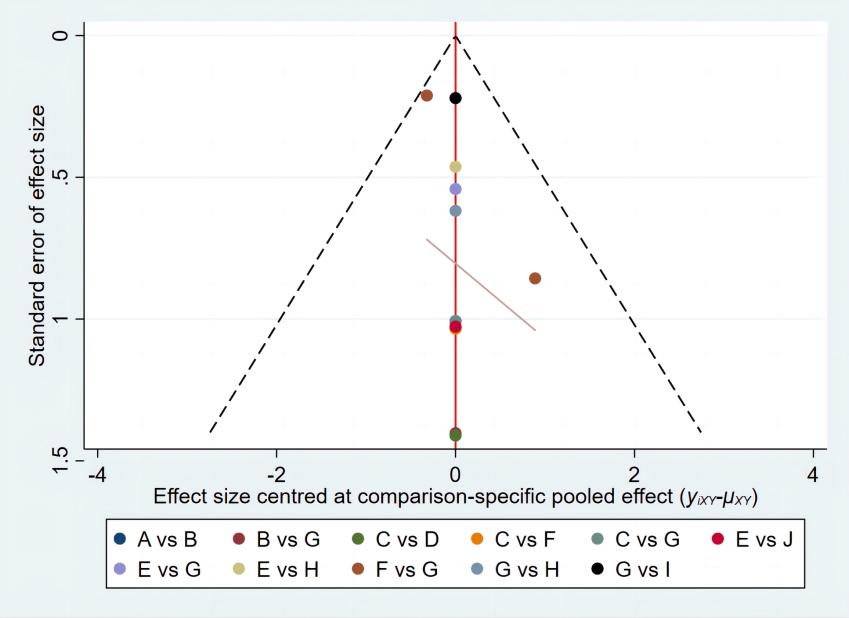
**

**Supplementary Figure S19E** Funnel plot for the network of Peripheral sensory neuropathy;


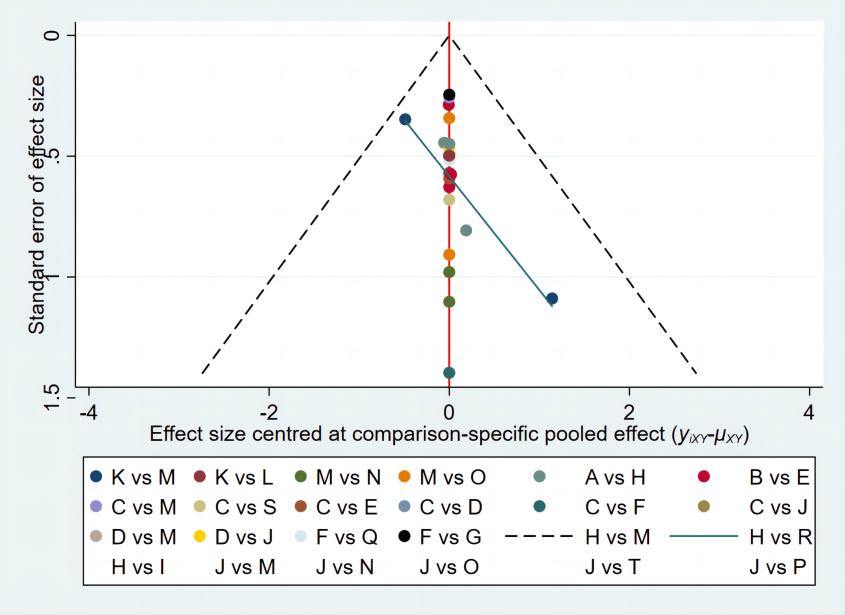


**Supplementary Figure S19F** Funnel plot for the network of Fatigue;


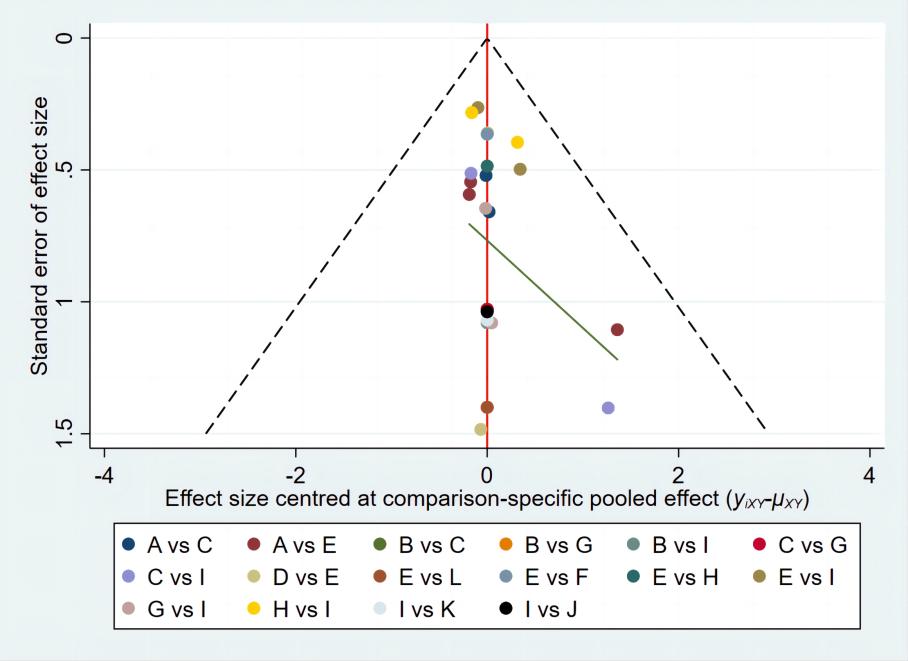


**Supplementary Figure S19G** Funnel plot for the network of Hypertension;


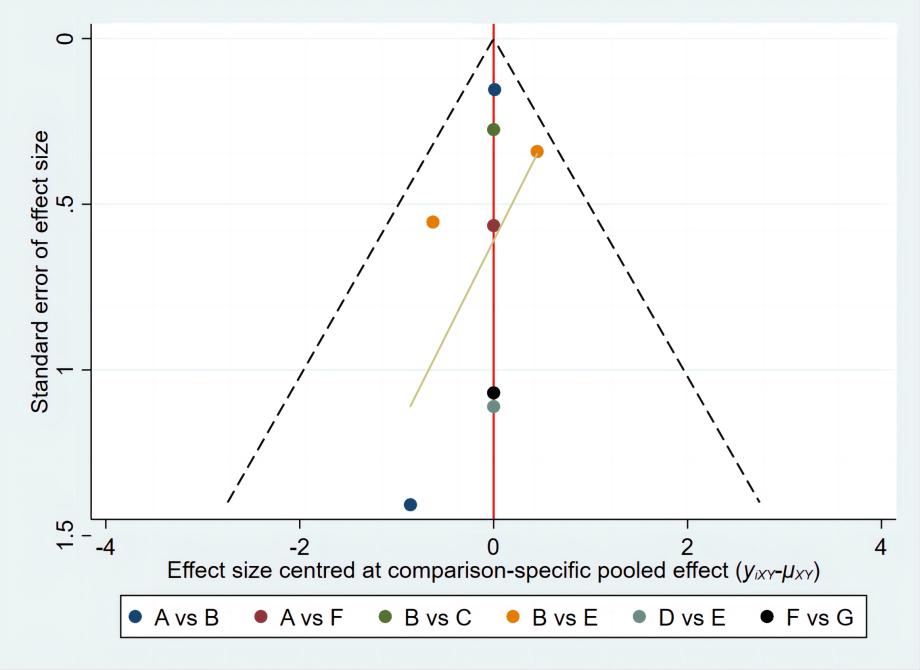


**Supplementary Figure S19H** Funnel plot for the network of Thromboembolic events.

**Supplementary Figure S19 Funnel plot.** Funnel plot for the network of (A) Nausea; (B) Vomiting; (C) Mucositis/stomatitis; (D) Anorexia; (E) Peripheral sensory neuropathy; (F) Fatigue; (G) Hypertension; (H) Thromboembolic events.
