# Supplementary material for: Safety of first-line systemic therapy in patients with metastatic colorectal cancer: a network meta-analysis of randomized controlled trials
Source: BMC Cancer. 2024 Jul 24;24:893. doi: 10.1186/s12885-024-12662-3 (PMC11270896; doi:10.1186/s12885-024-12662-3)
Supplement: Supplementary file 3 — Supplementary Material 3 [file 12885_2024_12662_MOESM3_ESM.docx]

**Appendix 1 Search Strategies**

Pubmed Search Strategy

| Search number | Query | Results |
| --- | --- | --- |
| #1 | "Metastases"[Title/Abstract] OR "Metastasis"[Title/Abstract] OR "metastas*"[Title/Abstract] OR "Metastatic"[Title/Abstract] | 613887 |
| #2 | ((((Colorectal Neoplasms[MeSH Terms]) OR (Intestinal Neoplasms[MeSH Terms])) OR (Cecal Neoplasms[MeSH Terms])) OR (Colonic Neoplasms[MeSH Terms])) OR (Rectal Neoplasms[MeSH Terms]) | 266053 |
| #3 | "Adenocarcinoma"[Title/Abstract] OR "bowel tumor"[Title/Abstract] OR "bowel tumour"[Title/Abstract] OR "caecal neoplasm"[Title/Abstract] OR "caecal tumor"[Title/Abstract] OR "caecal tumour"[Title/Abstract] OR "caecum tumor"[Title/Abstract] OR "caecum tumour"[Title/Abstract] OR "Cecal Cancer"[Title/Abstract] OR "Cecal Neoplasm*"[Title/Abstract] OR "cecal tumor"[Title/Abstract] OR "cecal tumour"[Title/Abstract] OR "cecum tumor"[Title/Abstract] OR "cecum tumour"[Title/Abstract] OR "coecum tumor"[Title/Abstract] OR "coecum tumour"[Title/Abstract] OR "Colon Adenocarcinoma*"[Title/Abstract] OR "Colon Cancer*"[Title/Abstract] OR "colon mass (tumor)"[Title/Abstract] OR "colon neoplasia"[Title/Abstract] OR "Colon Neoplasm*"[Title/Abstract] OR "colon tumor"[Title/Abstract] OR "colon tumorigenesis"[Title/Abstract] OR "colon tumour"[Title/Abstract] OR "colon villous tumor"[Title/Abstract] OR "colon villous tumour"[Title/Abstract] OR "Colonic Cancer*"[Title/Abstract] OR "colonic mass (tumor)"[Title/Abstract] OR "colonic masses (tumor)"[Title/Abstract] OR "colonic neoplasia"[Title/Abstract] OR "Colonic Neoplasm*"[Title/Abstract] OR "colonic tumor"[Title/Abstract] OR "colonic tumorigenesis"[Title/Abstract] OR "colonic tumour"[Title/Abstract] OR "Colorectal Cancer*"[Title/Abstract] OR "Colorectal Carcinoma*"[Title/Abstract] OR "colorectal neoplasia"[Title/Abstract] OR "colorectal neoplasm*"[Title/Abstract] OR "colorectal tumor*"[Title/Abstract] OR "colorectal tumour"[Title/Abstract] OR "gut neoplasia"[Title/Abstract] OR "gut tumor"[Title/Abstract] OR "gut tumorigenesis"[Title/Abstract] OR "gut tumour"[Title/Abstract] OR "intestinal canal tumor"[Title/Abstract] OR "intestinal canal tumour"[Title/Abstract] OR "Intestinal Cancer*"[Title/Abstract] OR "intestinal neoplasia"[Title/Abstract] OR "Intestinal Neoplasm*"[Title/Abstract] OR "intestinal tract tumor"[Title/Abstract] OR "intestinal tract tumour"[Title/Abstract] OR "intestinal tumor"[Title/Abstract] OR "intestinal tumorigenesis"[Title/Abstract] OR "intestinal tumour"[Title/Abstract] OR "intestine neoplasia"[Title/Abstract] OR "Intestine Neoplasm*"[Title/Abstract] OR "intestine tumor"[Title/Abstract] OR "intestine tumour*"[Title/Abstract] OR "Intestines Cancer*"[Title/Abstract] OR "mesocolon tumor"[Title/Abstract] OR "mesocolon tumour"[Title/Abstract] OR "neoplasm of the cecum"[Title/Abstract] OR "neoplasma recti"[Title/Abstract] OR "neoplasms of the gut"[Title/Abstract] OR "neoplastic colon"[Title/Abstract] OR "neoplastic colonic"[Title/Abstract] OR "neoplastic colorectal"[Title/Abstract] OR "neoplastic intestinal"[Title/Abstract] OR "pararectal tumor"[Title/Abstract] OR "pararectal tumour"[Title/Abstract] OR "Rectal Cancer*"[Title/Abstract] OR "rectal mass (tumor)"[Title/Abstract] OR "rectal neoplasia"[Title/Abstract] OR "Rectal Neoplasm*"[Title/Abstract] OR "Rectal Tumor*"[Title/Abstract] OR "rectal tumour"[Title/Abstract] OR "Rectum Cancer*"[Title/Abstract] OR "rectum mass (tumor)"[Title/Abstract] OR "rectum neoplasia"[Title/Abstract] OR "Rectum Neoplasm*"[Title/Abstract] OR "rectum tumor"[Title/Abstract] OR "rectum tumour"[Title/Abstract] OR "retrorectal tumor"[Title/Abstract] OR "retrorectal tumour"[Title/Abstract] OR "tumor recti"[Title/Abstract] OR "tumour recti"[Title/Abstract] | 382136 |
| #4 | "colorectal"[Title/Abstract] OR "colon"[Title/Abstract] OR "colonic"[Title/Abstract] OR "rectum"[Title/Abstract] OR "rectal"[Title/Abstract] OR "bowel*"[Title/Abstract] OR "intestine*"[Title/Abstract] OR "sigmoid"[Title/Abstract] | 754894 |
| #5 | "neoplas*"[Title/Abstract] OR "tumor*"[Title/Abstract] OR "tumour*"[Title/Abstract] OR "cancer*"[Title/Abstract] OR "carcinoma*"[Title/Abstract] OR "malignan*"[Title/Abstract] OR "adenocarcinoma*"[Title/Abstract] OR "adenom*"[Title/Abstract] | 4080386 |
| #6 | #4 AND #5 | 360513 |
| #7 | #2 OR #3 OR #6 | 556887 |
| #8 | ((((((((((((((((((((((((((((((Adavosertib[MeSH Terms]) OR (Aflibercept[MeSH Terms])) OR (anlotinib[MeSH Terms])) OR (Antibodies, Monoclonal[MeSH Terms])) OR (atezolizumab[MeSH Terms])) OR (Bevacizumab[MeSH Terms])) OR (Binimetinib[MeSH Terms])) OR (Capecitabine[MeSH Terms])) OR (Cetuximab[MeSH Terms])) OR (Dabrafenib[MeSH Terms])) OR (Encorafenib[MeSH Terms])) OR (Fluorouracil[MeSH Terms])) OR (Ipilimumab[MeSH Terms])) OR (Irinotecan[MeSH Terms])) OR (Lapatinib[MeSH Terms])) OR (lenvatinib[MeSH Terms])) OR (Nivolumab[MeSH Terms])) OR (Oxaliplatin[MeSH Terms])) OR (Panitumumab[MeSH Terms])) OR (pembrolizumab[MeSH Terms])) OR (pembrolizumab[MeSH Terms])) OR (Pertuzumab[MeSH Terms])) OR (ramucirumab[MeSH Terms])) OR (Regorafenib[MeSH Terms])) OR (Temozolomide[MeSH Terms])) OR (Tipiracil[MeSH Terms])) OR (trametinib[MeSH Terms])) OR (Trastuzumab[MeSH Terms])) OR (Trifluridine[MeSH Terms])) OR (Vascular Endothelial Growth Factors[MeSH Terms])) OR (Vemurafenib[MeSH Terms]) | 397811 |
| #9 | "1121B"[Title/Abstract] OR "2C4"[Title/Abstract] OR "5 HU Hexal"[Title/Abstract] OR "5FU"[Title/Abstract] OR "a168"[Title/Abstract] OR "abevmy"[Title/Abstract] OR "abp215"[Title/Abstract] OR "abp494"[Title/Abstract] OR "abp938"[Title/Abstract] OR "abp980"[Title/Abstract] OR "ABX EGF"[Title/Abstract] OR "accusite"[Title/Abstract] OR "ACT078"[Title/Abstract] OR "actino-hermal"[Title/Abstract] OR "Adavosertib "[Title/Abstract] OR "adrucil"[Title/Abstract] OR "advecit"[Title/Abstract] OR "aflibercept"[Title/Abstract] OR "aflomin"[Title/Abstract] OR "agicil"[Title/Abstract] OR "ainex"[Title/Abstract] OR "aiv007"[Title/Abstract] OR "AL3818"[Title/Abstract] OR "altl9"[Title/Abstract] OR "altuzan"[Title/Abstract] OR "alymsys"[Title/Abstract] OR "amg954"[Title/Abstract] OR "amt901"[Title/Abstract] OR "ankeda"[Title/Abstract] OR "anlotinib"[Title/Abstract] OR "anti-PDL1"[Title/Abstract] OR "apecitab"[Title/Abstract] OR "arry162"[Title/Abstract] OR "arry438162"[Title/Abstract] OR "aryotrust"[Title/Abstract] OR "askb1202"[Title/Abstract] OR "atezolizumab"[Title/Abstract] OR "atubri"[Title/Abstract] OR "avastin"[Title/Abstract] OR "ave0005"[Title/Abstract] OR "ave005"[Title/Abstract] OR "avegra"[Title/Abstract] OR "avt06"[Title/Abstract] OR "axiplatin"[Title/Abstract] OR "aybintio"[Title/Abstract] OR "azd1775"[Title/Abstract] OR "ba1101"[Title/Abstract] OR "ba1104"[Title/Abstract] OR "balimek"[Title/Abstract] OR "bambevi"[Title/Abstract] OR "bat1706"[Title/Abstract] OR "bay734506"[Title/Abstract] OR "bay865321"[Title/Abstract] OR "bcd021"[Title/Abstract] OR "bcd022"[Title/Abstract] OR "bcd201"[Title/Abstract] OR "bendaplatin"[Title/Abstract] OR "bephen"[Title/Abstract] OR "bevacizumab"[Title/Abstract] OR "bevagen"[Title/Abstract] OR "bevatas"[Title/Abstract] OR "bevax"[Title/Abstract] OR "bevz92"[Title/Abstract] OR "bi695502"[Title/Abstract] OR "Binimetinib "[Title/Abstract] OR "blastomat"[Title/Abstract] OR "bms734016"[Title/Abstract] OR "bms936558"[Title/Abstract] OR "bow030"[Title/Abstract] OR "boyounuo"[Title/Abstract] OR "bp01"[Title/Abstract] OR "bp102"[Title/Abstract] OR "braftovi"[Title/Abstract] OR "bryxta"[Title/Abstract] OR "bs503a"[Title/Abstract] OR "bx2318"[Title/Abstract] OR "bxt2316"[Title/Abstract] OR "byvasda"[Title/Abstract] OR "c225"[Title/Abstract] OR "calmtop"[Title/Abstract] OR "campto"[Title/Abstract] OR "camptosar"[Title/Abstract] OR "Camptothecin 11"[Title/Abstract] OR "capcel"[Title/Abstract] OR "capebina"[Title/Abstract] OR "capecel"[Title/Abstract] OR "capecitabine"[Title/Abstract] OR "capecitalox"[Title/Abstract] OR "capecite"[Title/Abstract] OR "capegard"[Title/Abstract] OR "capezam"[Title/Abstract] OR "capibine"[Title/Abstract] OR "capicet"[Title/Abstract] OR "capiibine"[Title/Abstract] OR "capnat"[Title/Abstract] OR "capoda"[Title/Abstract] OR "capostat"[Title/Abstract] OR "CAPOX"[Title/Abstract] OR "capsy"[Title/Abstract] OR "capxcel"[Title/Abstract] OR "carac"[Title/Abstract] OR "caxeta"[Title/Abstract] OR "cbt124"[Title/Abstract] OR "ccrg81045"[Title/Abstract] OR "CCRG-81045"[Title/Abstract] OR "cdp1"[Title/Abstract] OR "cerex"[Title/Abstract] OR "cetuximab"[Title/Abstract] OR "ch225"[Title/Abstract] OR "chs2020"[Title/Abstract] OR "chs305"[Title/Abstract] OR "chs5217"[Title/Abstract] OR "cinkef u"[Title/Abstract] OR "citabin"[Title/Abstract] OR "cizumab"[Title/Abstract] OR "clonal antibody"[Title/Abstract] OR "cmab009"[Title/Abstract] OR "cmab819"[Title/Abstract] OR "coloxet"[Title/Abstract] OR "cpt11"[Title/Abstract] OR "crisapla"[Title/Abstract] OR "croloxat"[Title/Abstract] OR "cs1002"[Title/Abstract] OR "ctp06"[Title/Abstract] OR "ctp15"[Title/Abstract] OR "ctp16"[Title/Abstract] OR "ctp6"[Title/Abstract] OR "cyramza"[Title/Abstract] OR "da3111"[Title/Abstract] OR "dabrafenib"[Title/Abstract] OR "dacotin"[Title/Abstract] OR "dacplat"[Title/Abstract] OR "debio0507"[Title/Abstract] OR "dmb3111"[Title/Abstract] OR "dq2805"[Title/Abstract] OR "drb436"[Title/Abstract] OR "e7080"[Title/Abstract] OR "eb1003"[Title/Abstract] OR "ebeoxal"[Title/Abstract] OR "ecansya"[Title/Abstract] OR "effluderm"[Title/Abstract] OR "efudex"[Title/Abstract] OR "efudix"[Title/Abstract] OR "efurix"[Title/Abstract] OR "eg12014"[Title/Abstract] OR "EGFR"[Title/Abstract] OR "elatofen"[Title/Abstract] OR "eloxatin"[Title/Abstract] OR "eloxatine"[Title/Abstract] OR "elplat"[Title/Abstract] OR "encorafenib"[Title/Abstract] OR "epidermal growthfactor receptor"[Title/Abstract] OR "equidacent"[Title/Abstract] OR "er203492-00"[Title/Abstract] OR "erbitux"[Title/Abstract] OR "eurofluor"[Title/Abstract] OR "eurofluor (drug)"[Title/Abstract] OR "euroxaliplatin"[Title/Abstract] OR "eylea"[Title/Abstract] OR "eylia"[Title/Abstract] OR "f6627"[Title/Abstract] OR "first-line treatment"[Title/Abstract] OR "fivoflu"[Title/Abstract] OR "fkb238"[Title/Abstract] OR "fluoro uracil"[Title/Abstract] OR "fluoroblastin"[Title/Abstract] OR "fluoroplex"[Title/Abstract] OR "fluoropyrimidine"[Title/Abstract] OR "fluorouracil"[Title/Abstract] OR "fluorouracile"[Title/Abstract] OR "Fluoro-Uracile ICN"[Title/Abstract] OR "Fluorouracil-GRY"[Title/Abstract] OR "fluorouracilo"[Title/Abstract] OR "fluoruracil"[Title/Abstract] OR "fluouracil"[Title/Abstract] OR "fluoxan"[Title/Abstract] OR "flurablastin"[Title/Abstract] OR "fluracedyl"[Title/Abstract] OR "fluracil"[Title/Abstract] OR "fluracilium"[Title/Abstract] OR "fluril"[Title/Abstract] OR "fluro uracil"[Title/Abstract] OR "fluroblastin"[Title/Abstract] OR "fluroblastine"[Title/Abstract] OR "Flurodex"[Title/Abstract] OR "FOLFIRI"[Title/Abstract] OR "FOLFOXIRI "[Title/Abstract] OR "fyb203"[Title/Abstract] OR "gb222"[Title/Abstract] OR "gbs004"[Title/Abstract] OR "gbs012"[Title/Abstract] OR "geneplatin"[Title/Abstract] OR "gessedil"[Title/Abstract] OR "gsk1120212"[Title/Abstract] OR "gsk2118436"[Title/Abstract] OR "gw2016"[Title/Abstract] OR "GW282974X"[Title/Abstract] OR "gw572016"[Title/Abstract] OR "Haemato FU"[Title/Abstract] OR "hanbeitai"[Title/Abstract] OR "hd201"[Title/Abstract] OR "hd204"[Title/Abstract] OR "heloxatin"[Title/Abstract] OR "Herceptin"[Title/Abstract] OR "herclon"[Title/Abstract] OR "hermyl1401o"[Title/Abstract] OR "herticad"[Title/Abstract] OR "hertraz"[Title/Abstract] OR "hervelous"[Title/Abstract] OR "herzuma"[Title/Abstract] OR "hlx02"[Title/Abstract] OR "hlx04"[Title/Abstract] OR "hlx12"[Title/Abstract] OR "hlx13"[Title/Abstract] OR "hot1010"[Title/Abstract] OR "hs627"[Title/Abstract] OR "Human Panitumumab Antibody"[Title/Abstract] OR "hybridoma antibody"[Title/Abstract] OR "ibi305"[Title/Abstract] OR "ibi310"[Title/Abstract] OR "idb0072"[Title/Abstract] OR "ifacil"[Title/Abstract] OR "ihl305"[Title/Abstract] OR "IMC1121B"[Title/Abstract] OR "imc225"[Title/Abstract] OR "imcc225"[Title/Abstract] OR "immunotherapy or targeted therapy"[Title/Abstract] OR "intp24"[Title/Abstract] OR "Ipilimumab"[Title/Abstract] OR "ipique"[Title/Abstract] OR "Irinotecan"[Title/Abstract] OR "Irinotecan Hydrochloride"[Title/Abstract] OR "irinotel"[Title/Abstract] OR "Irrinotecan"[Title/Abstract] OR "jhl1149"[Title/Abstract] OR "jm83"[Title/Abstract] OR "js501"[Title/Abstract] OR "jtp74057"[Title/Abstract] OR "jy028"[Title/Abstract] OR "kanjinti"[Title/Abstract] OR "kapetral"[Title/Abstract] OR "Keytruda"[Title/Abstract] OR "kimozo"[Title/Abstract] OR "kisplyx"[Title/Abstract] OR "kl140"[Title/Abstract] OR "krabeva"[Title/Abstract] OR "kyomarc"[Title/Abstract] OR "lambrolizumab"[Title/Abstract] OR "lapatinib"[Title/Abstract] OR "lapatinib tosylate"[Title/Abstract] OR "lenvatinib"[Title/Abstract] OR "lenvima"[Title/Abstract] OR "Leucovorin or 5-FU"[Title/Abstract] OR "lextemy"[Title/Abstract] OR "LGX818"[Title/Abstract] OR "liploxa"[Title/Abstract] OR "lipoxal"[Title/Abstract] OR "L-OHP Cpd"[Title/Abstract] OR "lumiere (drug)"[Title/Abstract] OR "ly01008"[Title/Abstract] OR "ly01012"[Title/Abstract] OR "ly01015"[Title/Abstract] OR "ly09004"[Title/Abstract] OR "ly2939777"[Title/Abstract] OR "LY3009806"[Title/Abstract] OR "M and B39831"[Title/Abstract] OR "MAb C225"[Title/Abstract] OR "mabionvegf"[Title/Abstract] OR "mb 39831"[Title/Abstract] OR "mb02"[Title/Abstract] OR "mb39831"[Title/Abstract] OR "mbp426"[Title/Abstract] OR "MDX CTLA 4"[Title/Abstract] OR "MDX010"[Title/Abstract] OR "mdx101"[Title/Abstract] OR "MDX1106"[Title/Abstract] OR "medoxa"[Title/Abstract] OR "MEK162"[Title/Abstract] OR "mekinist"[Title/Abstract] OR "Mektovi"[Title/Abstract] OR "Methazolastone"[Title/Abstract] OR "mil60"[Title/Abstract] OR "MK1775"[Title/Abstract] OR "mk3475"[Title/Abstract] OR "mk7365"[Title/Abstract] OR "mk7902"[Title/Abstract] OR "Monoclonal Antibod*"[Title/Abstract] OR "MPDL3280A"[Title/Abstract] OR "Mvasi"[Title/Abstract] OR "myl1401o"[Title/Abstract] OR "myl14020"[Title/Abstract] OR "myl1402o"[Title/Abstract] OR "myl1701p"[Title/Abstract] OR "naprocap"[Title/Abstract] OR "nc4016"[Title/Abstract] OR "Neofluor"[Title/Abstract] OR "Nivolumab"[Title/Abstract] OR "NK012 Compound"[Title/Abstract] OR "nogron"[Title/Abstract] OR "nsc18913"[Title/Abstract] OR "nsc19893"[Title/Abstract] OR "NSC362856"[Title/Abstract] OR "nsc704865"[Title/Abstract] OR "nvp lgx818"[Title/Abstract] OR "ocufridine"[Title/Abstract] OR "ogivri"[Title/Abstract] OR "oksaliplatin"[Title/Abstract] OR "oksaliplatina"[Title/Abstract] OR "Omnitarg"[Title/Abstract] OR "onbevzi"[Title/Abstract] OR "oncofu"[Title/Abstract] OR "Onkofluor"[Title/Abstract] OR "ONO4538"[Title/Abstract] OR "ono7702"[Title/Abstract] OR "ono7703"[Title/Abstract] OR "ons1045"[Title/Abstract] OR "ons1050"[Title/Abstract] OR "ons5010"[Title/Abstract] OR "ontruzant"[Title/Abstract] OR "Opdivo"[Title/Abstract] OR "oplat"[Title/Abstract] OR "orp005"[Title/Abstract] OR "ot702"[Title/Abstract] OR "oxalatoplatinum"[Title/Abstract] OR "oxalatplatin"[Title/Abstract] OR "oxali"[Title/Abstract] OR "oxalip"[Title/Abstract] OR "oxaliplan"[Title/Abstract] OR "Oxaliplatin"[Title/Abstract] OR "oxaliplatina"[Title/Abstract] OR "Oxaliplatine"[Title/Abstract] OR "oxaliplatino"[Title/Abstract] OR "oxaliplatinum"[Title/Abstract] OR "oxaliprol"[Title/Abstract] OR "oxaliquid"[Title/Abstract] OR "oxalisan"[Title/Abstract] OR "oxalisin"[Title/Abstract] OR "oxalizor"[Title/Abstract] OR "oxaltic"[Title/Abstract] OR "oxaltina"[Title/Abstract] OR "oxaplamyl"[Title/Abstract] OR "oxaviatin"[Title/Abstract] OR "oyavas"[Title/Abstract] OR "panitumab"[Title/Abstract] OR "panitunumab"[Title/Abstract] OR "pbp1602"[Title/Abstract] OR "pbp1701"[Title/Abstract] OR "pbp2001"[Title/Abstract] OR "pbp2101"[Title/Abstract] OR "pbp2102"[Title/Abstract] OR "pembrolizumab"[Title/Abstract] OR "Perjeta"[Title/Abstract] OR "Pertuzumab"[Title/Abstract] OR "pf05280014"[Title/Abstract] OR "pf06439535"[Title/Abstract] OR "pf06811462"[Title/Abstract] OR "pf07263896"[Title/Abstract] OR "pf5280014"[Title/Abstract] OR "pf6439535"[Title/Abstract] OR "pf6811462"[Title/Abstract] OR "pf7263896"[Title/Abstract] OR "platox"[Title/Abstract] OR "plaxitin"[Title/Abstract] OR "PLX4032"[Title/Abstract] OR "pmc901"[Title/Abstract] OR "pmc902"[Title/Abstract] OR "pobevcy"[Title/Abstract] OR "preveloda"[Title/Abstract] OR "pro169"[Title/Abstract] OR "pusintin"[Title/Abstract] OR "ql1101"[Title/Abstract] OR "ql1207"[Title/Abstract] OR "ql1209"[Title/Abstract] OR "r tpr 023"[Title/Abstract] OR "R05185426"[Title/Abstract] OR "r1273"[Title/Abstract] OR "r340"[Title/Abstract] OR "r435"[Title/Abstract] OR "r597"[Title/Abstract] OR "r7204"[Title/Abstract] OR "ramucirumab"[Title/Abstract] OR "rectoxal"[Title/Abstract] OR "regn3"[Title/Abstract] OR "regorafenib"[Title/Abstract] OR "resihance"[Title/Abstract] OR "rg1273"[Title/Abstract] OR "rg435"[Title/Abstract] OR "rg597"[Title/Abstract] OR "RG7204"[Title/Abstract] OR "RG7446"[Title/Abstract] OR "rhumab 2C4"[Title/Abstract] OR "rhuMAb-VEGF"[Title/Abstract] OR "Ribofluor"[Title/Abstract] OR "riboxatin"[Title/Abstract] OR "ridoca"[Title/Abstract] OR "ro0452317"[Title/Abstract] OR "ro091978"[Title/Abstract] OR "ro2 9757"[Title/Abstract] OR "ro4368451"[Title/Abstract] OR "ro4876646"[Title/Abstract] OR "ro5185426"[Title/Abstract] OR "ro5541267"[Title/Abstract] OR "ro7069680"[Title/Abstract] OR "ro7071618"[Title/Abstract] OR "ro7234952"[Title/Abstract] OR "rp46161"[Title/Abstract] OR "rp54780"[Title/Abstract] OR "rph001"[Title/Abstract] OR "rtpr023"[Title/Abstract] OR "samfenet"[Title/Abstract] OR "sb15"[Title/Abstract] OR "sb3"[Title/Abstract] OR "sb8"[Title/Abstract] OR "scb420"[Title/Abstract] OR "scd411"[Title/Abstract] OR "sch052365"[Title/Abstract] OR "sch52365"[Title/Abstract] OR "sch900475"[Title/Abstract] OR "sct501"[Title/Abstract] OR "sct510"[Title/Abstract] OR "si053"[Title/Abstract] OR "sibp01"[Title/Abstract] OR "sinoxal"[Title/Abstract] OR "SN 38"[Title/Abstract] OR "SN3811"[Title/Abstract] OR "snr1611"[Title/Abstract] OR "sok583a1"[Title/Abstract] OR "sr96669"[Title/Abstract] OR "stc103"[Title/Abstract] OR "sti001"[Title/Abstract] OR "stivant"[Title/Abstract] OR "Stivarga"[Title/Abstract] OR "strentarga"[Title/Abstract] OR "syd977"[Title/Abstract] OR "syn112"[Title/Abstract] OR "tab008"[Title/Abstract] OR "tab014"[Title/Abstract] OR "tafinlar"[Title/Abstract] OR "tas1-462"[Title/Abstract] OR "Tecentriq"[Title/Abstract] OR "tecntriq"[Title/Abstract] OR "temazol"[Title/Abstract] OR "temcad"[Title/Abstract] OR "temodal"[Title/Abstract] OR "Temodar"[Title/Abstract] OR "temodex"[Title/Abstract] OR "temodol"[Title/Abstract] OR "temomedac"[Title/Abstract] OR "temostad"[Title/Abstract] OR "temoxol"[Title/Abstract] OR "temozo-cell"[Title/Abstract] OR "Temozolomide"[Title/Abstract] OR "tft"[Title/Abstract] OR "thriherpine"[Title/Abstract] OR "tio217"[Title/Abstract] OR "Tipiracil"[Title/Abstract] OR "tmt212"[Title/Abstract] OR "TMZ Bioshuttle"[Title/Abstract] OR "TMZA-HE"[Title/Abstract] OR "tolak"[Title/Abstract] OR "topotecin"[Title/Abstract] OR "tot102"[Title/Abstract] OR "trametinib"[Title/Abstract] OR "transplastin"[Title/Abstract] OR "trasturel"[Title/Abstract] OR "Trastuzumab"[Title/Abstract] OR "Trazimera"[Title/Abstract] OR "Triflumann"[Title/Abstract] OR "trifluor thymidine"[Title/Abstract] OR "Trifluoridine"[Title/Abstract] OR "trifluoro thymidine"[Title/Abstract] OR "trifluorodeoxythymidine"[Title/Abstract] OR "Trifluorothymidine"[Title/Abstract] OR "Trifluridine"[Title/Abstract] OR "trifuridine"[Title/Abstract] OR "triherpin"[Title/Abstract] OR "triherpine"[Title/Abstract] OR "trs003"[Title/Abstract] OR "tuznue"[Title/Abstract] OR "tx05"[Title/Abstract] OR "tx16"[Title/Abstract] OR "Tykerb"[Title/Abstract] OR "tyverb"[Title/Abstract] OR "u101440e"[Title/Abstract] OR "ub921"[Title/Abstract] OR "uflahex"[Title/Abstract] OR "uraciflor"[Title/Abstract] OR "utoral"[Title/Abstract] OR "vascular endothelial growth factor*"[Title/Abstract] OR "vasculotropin trap"[Title/Abstract] OR "vectibex"[Title/Abstract] OR "Vectibix"[Title/Abstract] OR "VEGF*"[Title/Abstract] OR "vegzelma"[Title/Abstract] OR "velminox"[Title/Abstract] OR "Vemurafenib"[Title/Abstract] OR "versavo"[Title/Abstract] OR "Viromidin"[Title/Abstract] OR "virophta"[Title/Abstract] OR "viroptic"[Title/Abstract] OR "vivitra"[Title/Abstract] OR "w0090"[Title/Abstract] OR "wetlia"[Title/Abstract] OR "xabine"[Title/Abstract] OR "xaliplat"[Title/Abstract] OR "xalvobin"[Title/Abstract] OR "xdivane"[Title/Abstract] OR "xecap"[Title/Abstract] OR "xelazor"[Title/Abstract] OR "xelcip"[Title/Abstract] OR "xelocel"[Title/Abstract] OR "Xeloda"[Title/Abstract] OR "xoplan"[Title/Abstract] OR "xtrudane"[Title/Abstract] OR "Yervoy"[Title/Abstract] OR "Zaltrap"[Title/Abstract] OR "zedora"[Title/Abstract] OR "Zelboraf"[Title/Abstract] OR "zercepac"[Title/Abstract] OR "zerectum"[Title/Abstract] OR "zirabev"[Title/Abstract] OR "ziv aflibercept"[Title/Abstract] OR "zocitab"[Title/Abstract] OR "zrc113"[Title/Abstract] OR "zybev"[Title/Abstract] | 534698 |
| #10 | #8 OR #9 | 680181 |
| #11 | "Random"[Title/Abstract] OR "Randomized"[Title/Abstract] OR "trail*"[Title/Abstract] OR "Control"[Title/Abstract] OR "Controlled"[Title/Abstract] | 4391288 |
| #12 | #1 AND #7 AND #10 AND #11 | 4986 |

Embase Search Strategy

| Search number | Query | Results |
| --- | --- | --- |
| #1 | 'metastases':ab,ti,kw OR 'metastasis':ab,ti,kw OR 'metastas*':ab,ti,kw OR 'metastatic':ab,ti,kw | 910828 |
| #2 | 'colorectal tumor'/exp OR 'intestine tumor'/exp OR 'cecum tumor'/exp OR 'colon tumor'/exp OR 'rectum tumor'/exp | 532626 |
| #3 | 'adenocarcinoma':ab,ti,kw OR 'bowel tumor':ab,ti,kw OR 'bowel tumour':ab,ti,kw OR 'caecal neoplasm':ab,ti,kw OR 'caecal tumor':ab,ti,kw OR 'caecal tumour':ab,ti,kw OR 'caecum tumor':ab,ti,kw OR 'caecum tumour':ab,ti,kw OR 'cecal cancer':ab,ti,kw OR 'cecal neoplasm*':ab,ti,kw OR 'cecal tumor':ab,ti,kw OR 'cecal tumour':ab,ti,kw OR 'cecum tumor':ab,ti,kw OR 'cecum tumour':ab,ti,kw OR 'coecum tumor':ab,ti,kw OR 'coecum tumour':ab,ti,kw OR 'colon adenocarcinoma*':ab,ti,kw OR 'colon cancer*':ab,ti,kw OR 'colon mass (tumor)':ab,ti,kw OR 'colon neoplasia':ab,ti,kw OR 'colon neoplasm*':ab,ti,kw OR 'colon tumor':ab,ti,kw OR 'colon tumorigenesis':ab,ti,kw OR 'colon tumour':ab,ti,kw OR 'colon villous tumor':ab,ti,kw OR 'colon villous tumour':ab,ti,kw OR 'colonic cancer*':ab,ti,kw OR 'colonic mass (tumor)':ab,ti,kw OR 'colonic masses (tumor)':ab,ti,kw OR 'colonic neoplasia':ab,ti,kw OR 'colonic neoplasm*':ab,ti,kw OR 'colonic tumor':ab,ti,kw OR 'colonic tumorigenesis':ab,ti,kw OR 'colonic tumour':ab,ti,kw OR 'colorectal cancer*':ab,ti,kw OR 'colorectal carcinoma*':ab,ti,kw OR 'colorectal neoplasia':ab,ti,kw OR 'colorectal neoplasm*':ab,ti,kw OR 'colorectal tumor*':ab,ti,kw OR 'colorectal tumour':ab,ti,kw OR 'gut neoplasia':ab,ti,kw OR 'gut tumor':ab,ti,kw OR 'gut tumorigenesis':ab,ti,kw OR 'gut tumour':ab,ti,kw OR 'intestinal canal tumor':ab,ti,kw OR 'intestinal canal tumour':ab,ti,kw OR 'intestinal cancer*':ab,ti,kw OR 'intestinal neoplasia':ab,ti,kw OR 'intestinal neoplasm*':ab,ti,kw OR 'intestinal tract tumor':ab,ti,kw OR 'intestinal tract tumour':ab,ti,kw OR 'intestinal tumor':ab,ti,kw OR 'intestinal tumorigenesis':ab,ti,kw OR 'intestinal tumour':ab,ti,kw OR 'intestine neoplasia':ab,ti,kw OR 'intestine neoplasm*':ab,ti,kw OR 'intestine tumor':ab,ti,kw OR 'intestine tumour*':ab,ti,kw OR 'intestines cancer*':ab,ti,kw OR 'mesocolon tumor':ab,ti,kw OR 'mesocolon tumour':ab,ti,kw OR 'neoplasm of the cecum':ab,ti,kw OR 'neoplasma recti':ab,ti,kw OR 'neoplasms of the gut':ab,ti,kw OR 'neoplastic colon':ab,ti,kw OR 'neoplastic colonic':ab,ti,kw OR 'neoplastic colorectal':ab,ti,kw OR 'neoplastic intestinal':ab,ti,kw OR 'pararectal tumor':ab,ti,kw OR 'pararectal tumour':ab,ti,kw OR 'rectal cancer*':ab,ti,kw OR 'rectal mass (tumor)':ab,ti,kw OR 'rectal neoplasia':ab,ti,kw OR 'rectal neoplasm*':ab,ti,kw OR 'rectal tumor*':ab,ti,kw OR 'rectal tumour':ab,ti,kw OR 'rectum cancer*':ab,ti,kw OR 'rectum mass (tumor)':ab,ti,kw OR 'rectum neoplasia':ab,ti,kw OR 'rectum neoplasm*':ab,ti,kw OR 'rectum tumor':ab,ti,kw OR 'rectum tumour':ab,ti,kw OR 'retrorectal tumor':ab,ti,kw OR 'retrorectal tumour':ab,ti,kw OR 'tumor recti':ab,ti,kw OR 'tumour recti':ab,ti,kw | 566610 |
| #4 | 'colorectal':ab,ti,kw OR 'colon':ab,ti,kw OR 'colonic':ab,ti,kw OR 'rectum':ab,ti,kw OR 'rectal':ab,ti,kw OR 'bowel*':ab,ti,kw OR 'intestine*':ab,ti,kw OR 'sigmoid':ab,ti,kw | 1083892 |
| #5 | 'neoplas*':ab,ti,kw OR 'tumor*':ab,ti,kw OR 'tumour*':ab,ti,kw OR 'cancer*':ab,ti,kw OR 'carcinoma*':ab,ti,kw OR 'malignan*':ab,ti,kw OR 'adenocarcinoma*':ab,ti,kw OR 'adenom*':ab,ti,kw | 5588870 |
| #6 | #4 AND #5 | 542907 |
| #7 | #2 OR #3 OR #6 | 897390 |
| #8 | 'adavosertib'/exp OR 'aflibercept'/exp OR 'atezolizumab'/exp OR 'bevacizumab'/exp OR 'binimetinib'/exp OR 'capecitabine'/exp OR 'cetuximab'/exp OR 'dabrafenib'/exp OR 'encorafenib'/exp OR 'fluorouracil'/exp OR 'ipilimumab'/exp OR 'irinotecan'/exp OR 'lapatinib'/exp OR 'lenvatinib'/exp OR 'monoclonal antibody'/exp OR 'nivolumab'/exp OR 'oxaliplatin'/exp OR 'panitumumab'/exp OR 'pembrolizumab'/exp OR 'pertuzumab'/exp OR 'ramucirumab'/exp OR 'regorafenib'/exp OR 'temozolomide'/exp OR 'tipiracil'/exp OR 'trametinib'/exp OR 'trastuzumab'/exp OR 'trifluridine'/exp OR 'vemurafenib'/exp | 976851 |
| #9 | '1121b':ab,ti,kw OR '2c4':ab,ti,kw OR '5 hu hexal':ab,ti,kw OR '5fu':ab,ti,kw OR 'a168':ab,ti,kw OR 'abevmy':ab,ti,kw OR 'abp215':ab,ti,kw OR 'abp494':ab,ti,kw OR 'abp938':ab,ti,kw OR 'abp980':ab,ti,kw OR 'abx egf':ab,ti,kw OR 'accusite':ab,ti,kw OR 'act078':ab,ti,kw OR 'actino-hermal':ab,ti,kw OR 'adavosertib':ab,ti,kw OR 'adrucil':ab,ti,kw OR 'advecit':ab,ti,kw OR 'aflibercept':ab,ti,kw OR 'aflomin':ab,ti,kw OR 'agicil':ab,ti,kw OR 'ainex':ab,ti,kw OR 'aiv007':ab,ti,kw OR 'al3818':ab,ti,kw OR 'altl9':ab,ti,kw OR 'altuzan':ab,ti,kw OR 'alymsys':ab,ti,kw OR 'amg954':ab,ti,kw OR 'amt901':ab,ti,kw OR 'ankeda':ab,ti,kw OR 'anlotinib':ab,ti,kw OR 'anti-pdl1':ab,ti,kw OR 'apecitab':ab,ti,kw OR 'arry162':ab,ti,kw OR 'arry438162':ab,ti,kw OR 'aryotrust':ab,ti,kw OR 'askb1202':ab,ti,kw OR 'atezolizumab':ab,ti,kw OR 'atubri':ab,ti,kw OR 'avastin':ab,ti,kw OR 'ave0005':ab,ti,kw OR 'ave005':ab,ti,kw OR 'avegra':ab,ti,kw OR 'avt06':ab,ti,kw OR 'axiplatin':ab,ti,kw OR 'aybintio':ab,ti,kw OR 'azd1775':ab,ti,kw OR 'ba1101':ab,ti,kw OR 'ba1104':ab,ti,kw OR 'balimek':ab,ti,kw OR 'bambevi':ab,ti,kw OR 'bat1706':ab,ti,kw OR 'bay734506':ab,ti,kw OR 'bay865321':ab,ti,kw OR 'bcd021':ab,ti,kw OR 'bcd022':ab,ti,kw OR 'bcd201':ab,ti,kw OR 'bendaplatin':ab,ti,kw OR 'bephen':ab,ti,kw OR 'bevacizumab':ab,ti,kw OR 'bevagen':ab,ti,kw OR 'bevatas':ab,ti,kw OR 'bevax':ab,ti,kw OR 'bevz92':ab,ti,kw OR 'bi695502':ab,ti,kw OR 'binimetinib':ab,ti,kw OR 'blastomat':ab,ti,kw OR 'bms734016':ab,ti,kw OR 'bms936558':ab,ti,kw OR 'bow030':ab,ti,kw OR 'boyounuo':ab,ti,kw OR 'bp01':ab,ti,kw OR 'bp102':ab,ti,kw OR 'braftovi':ab,ti,kw OR 'bryxta':ab,ti,kw OR 'bs503a':ab,ti,kw OR 'bx2318':ab,ti,kw OR 'bxt2316':ab,ti,kw OR 'byvasda':ab,ti,kw OR 'c225':ab,ti,kw OR 'calmtop':ab,ti,kw OR 'campto':ab,ti,kw OR 'camptosar':ab,ti,kw OR 'camptothecin 11':ab,ti,kw OR 'capcel':ab,ti,kw OR 'capebina':ab,ti,kw OR 'capecel':ab,ti,kw OR 'capecitabine':ab,ti,kw OR 'capecitalox':ab,ti,kw OR 'capecite':ab,ti,kw OR 'capegard':ab,ti,kw OR 'capezam':ab,ti,kw OR 'capibine':ab,ti,kw OR 'capicet':ab,ti,kw OR 'capiibine':ab,ti,kw OR 'capnat':ab,ti,kw OR 'capoda':ab,ti,kw OR 'capostat':ab,ti,kw OR 'capox':ab,ti,kw OR 'capsy':ab,ti,kw OR 'capxcel':ab,ti,kw OR 'carac':ab,ti,kw OR 'caxeta':ab,ti,kw OR 'cbt124':ab,ti,kw OR 'ccrg81045':ab,ti,kw OR 'ccrg-81045':ab,ti,kw OR 'cdp1':ab,ti,kw OR 'cerex':ab,ti,kw OR 'cetuximab':ab,ti,kw OR 'ch225':ab,ti,kw OR 'chs2020':ab,ti,kw OR 'chs305':ab,ti,kw OR 'chs5217':ab,ti,kw OR 'cinkef u':ab,ti,kw OR 'citabin':ab,ti,kw OR 'cizumab':ab,ti,kw OR 'clonal antibody':ab,ti,kw OR 'cmab009':ab,ti,kw OR 'cmab819':ab,ti,kw OR 'coloxet':ab,ti,kw OR 'cpt11':ab,ti,kw OR 'crisapla':ab,ti,kw OR 'croloxat':ab,ti,kw OR 'cs1002':ab,ti,kw OR 'ctp06':ab,ti,kw OR 'ctp15':ab,ti,kw OR 'ctp16':ab,ti,kw OR 'ctp6':ab,ti,kw OR 'cyramza':ab,ti,kw OR 'da3111':ab,ti,kw OR 'dabrafenib':ab,ti,kw OR 'dacotin':ab,ti,kw OR 'dacplat':ab,ti,kw OR 'debio0507':ab,ti,kw OR 'dmb3111':ab,ti,kw OR 'dq2805':ab,ti,kw OR 'drb436':ab,ti,kw OR 'e7080':ab,ti,kw OR 'eb1003':ab,ti,kw OR 'ebeoxal':ab,ti,kw OR 'ecansya':ab,ti,kw OR 'effluderm':ab,ti,kw OR 'efudex':ab,ti,kw OR 'efudix':ab,ti,kw OR 'efurix':ab,ti,kw OR 'eg12014':ab,ti,kw OR 'egfr':ab,ti,kw OR 'elatofen':ab,ti,kw OR 'eloxatin':ab,ti,kw OR 'eloxatine':ab,ti,kw OR 'elplat':ab,ti,kw OR 'encorafenib':ab,ti,kw OR 'epidermal growthfactor receptor':ab,ti,kw OR 'equidacent':ab,ti,kw OR 'er203492-00':ab,ti,kw OR 'erbitux':ab,ti,kw OR 'eurofluor':ab,ti,kw OR 'eurofluor (drug)':ab,ti,kw OR 'euroxaliplatin':ab,ti,kw OR 'eylea':ab,ti,kw OR 'eylia':ab,ti,kw OR 'f6627':ab,ti,kw OR 'first-line treatment':ab,ti,kw OR 'fivoflu':ab,ti,kw OR 'fkb238':ab,ti,kw OR 'fluoro uracil':ab,ti,kw OR 'fluoroblastin':ab,ti,kw OR 'fluoroplex':ab,ti,kw OR 'fluoropyrimidine':ab,ti,kw OR 'fluorouracil':ab,ti,kw OR 'fluorouracile':ab,ti,kw OR 'fluoro-uracile icn':ab,ti,kw OR 'fluorouracil-gry':ab,ti,kw OR 'fluorouracilo':ab,ti,kw OR 'fluoruracil':ab,ti,kw OR 'fluouracil':ab,ti,kw OR 'fluoxan':ab,ti,kw OR 'flurablastin':ab,ti,kw OR 'fluracedyl':ab,ti,kw OR 'fluracil':ab,ti,kw OR 'fluracilium':ab,ti,kw OR 'fluril':ab,ti,kw OR 'fluro uracil':ab,ti,kw OR 'fluroblastin':ab,ti,kw OR 'fluroblastine':ab,ti,kw OR 'flurodex':ab,ti,kw OR 'folfiri':ab,ti,kw OR 'folfoxiri':ab,ti,kw OR 'fyb203':ab,ti,kw OR 'gb222':ab,ti,kw OR 'gbs004':ab,ti,kw OR 'gbs012':ab,ti,kw OR 'geneplatin':ab,ti,kw OR 'gessedil':ab,ti,kw OR 'gsk1120212':ab,ti,kw OR 'gsk2118436':ab,ti,kw OR 'gw2016':ab,ti,kw OR 'gw282974x':ab,ti,kw OR 'gw572016':ab,ti,kw OR 'haemato fu':ab,ti,kw OR 'hanbeitai':ab,ti,kw OR 'hd201':ab,ti,kw OR 'hd204':ab,ti,kw OR 'heloxatin':ab,ti,kw OR 'herceptin':ab,ti,kw OR 'herclon':ab,ti,kw OR 'hermyl1401o':ab,ti,kw OR 'herticad':ab,ti,kw OR 'hertraz':ab,ti,kw OR 'hervelous':ab,ti,kw OR 'herzuma':ab,ti,kw OR 'hlx02':ab,ti,kw OR 'hlx04':ab,ti,kw OR 'hlx12':ab,ti,kw OR 'hlx13':ab,ti,kw OR 'hot1010':ab,ti,kw OR 'hs627':ab,ti,kw OR 'human panitumumab antibody':ab,ti,kw OR 'hybridoma antibody':ab,ti,kw OR 'ibi305':ab,ti,kw OR 'ibi310':ab,ti,kw OR 'idb0072':ab,ti,kw OR 'ifacil':ab,ti,kw OR 'ihl305':ab,ti,kw OR 'imc1121b':ab,ti,kw OR 'imc225':ab,ti,kw OR 'imcc225':ab,ti,kw OR 'immunotherapy or targeted therapy':ab,ti,kw OR 'intp24':ab,ti,kw OR 'ipilimumab':ab,ti,kw OR 'ipique':ab,ti,kw OR 'irinotecan':ab,ti,kw OR 'irinotecan hydrochloride':ab,ti,kw OR 'irinotel':ab,ti,kw OR 'irrinotecan':ab,ti,kw OR 'jhl1149':ab,ti,kw OR 'jm83':ab,ti,kw OR 'js501':ab,ti,kw OR 'jtp74057':ab,ti,kw OR 'jy028':ab,ti,kw OR 'kanjinti':ab,ti,kw OR 'kapetral':ab,ti,kw OR 'keytruda':ab,ti,kw OR 'kimozo':ab,ti,kw OR 'kisplyx':ab,ti,kw OR 'kl140':ab,ti,kw OR 'krabeva':ab,ti,kw OR 'kyomarc':ab,ti,kw OR 'lambrolizumab':ab,ti,kw OR 'lapatinib':ab,ti,kw OR 'lapatinib tosylate':ab,ti,kw OR 'lenvatinib':ab,ti,kw OR 'lenvima':ab,ti,kw OR 'leucovorin or 5-fu':ab,ti,kw OR 'lextemy':ab,ti,kw OR 'lgx818':ab,ti,kw OR 'liploxa':ab,ti,kw OR 'lipoxal':ab,ti,kw OR 'l-ohp cpd':ab,ti,kw OR 'lumiere (drug)':ab,ti,kw OR 'ly01008':ab,ti,kw OR 'ly01012':ab,ti,kw OR 'ly01015':ab,ti,kw OR 'ly09004':ab,ti,kw OR 'ly2939777':ab,ti,kw OR 'ly3009806':ab,ti,kw OR 'm and b39831':ab,ti,kw OR 'mab c225':ab,ti,kw OR 'mabionvegf':ab,ti,kw OR 'mb 39831':ab,ti,kw OR 'mb02':ab,ti,kw OR 'mb39831':ab,ti,kw OR 'mbp426':ab,ti,kw OR 'mdx ctla 4':ab,ti,kw OR 'mdx010':ab,ti,kw OR 'mdx101':ab,ti,kw OR 'mdx1106':ab,ti,kw OR 'medoxa':ab,ti,kw OR 'mek162':ab,ti,kw OR 'mekinist':ab,ti,kw OR 'mektovi':ab,ti,kw OR 'methazolastone':ab,ti,kw OR 'mil60':ab,ti,kw OR 'mk1775':ab,ti,kw OR 'mk3475':ab,ti,kw OR 'mk7365':ab,ti,kw OR 'mk7902':ab,ti,kw OR 'monoclonal antibod*':ab,ti,kw OR 'mpdl3280a':ab,ti,kw OR 'mvasi':ab,ti,kw OR 'myl1401o':ab,ti,kw OR 'myl14020':ab,ti,kw OR 'myl1402o':ab,ti,kw OR 'myl1701p':ab,ti,kw OR 'naprocap':ab,ti,kw OR 'nc4016':ab,ti,kw OR 'neofluor':ab,ti,kw OR 'nivolumab':ab,ti,kw OR 'nk012 compound':ab,ti,kw OR 'nogron':ab,ti,kw OR 'nsc18913':ab,ti,kw OR 'nsc19893':ab,ti,kw OR 'nsc362856':ab,ti,kw OR 'nsc704865':ab,ti,kw OR 'nvp lgx818':ab,ti,kw OR 'ocufridine':ab,ti,kw OR 'ogivri':ab,ti,kw OR 'oksaliplatin':ab,ti,kw OR 'oksaliplatina':ab,ti,kw OR 'omnitarg':ab,ti,kw OR 'onbevzi':ab,ti,kw OR 'oncofu':ab,ti,kw OR 'onkofluor':ab,ti,kw OR 'ono4538':ab,ti,kw OR 'ono7702':ab,ti,kw OR 'ono7703':ab,ti,kw OR 'ons1045':ab,ti,kw OR 'ons1050':ab,ti,kw OR 'ons5010':ab,ti,kw OR 'ontruzant':ab,ti,kw OR 'opdivo':ab,ti,kw OR 'oplat':ab,ti,kw OR 'orp005':ab,ti,kw OR 'ot702':ab,ti,kw OR 'oxalatoplatinum':ab,ti,kw OR 'oxalatplatin':ab,ti,kw OR 'oxali':ab,ti,kw OR 'oxalip':ab,ti,kw OR 'oxaliplan':ab,ti,kw OR 'oxaliplatin':ab,ti,kw OR 'oxaliplatina':ab,ti,kw OR 'oxaliplatine':ab,ti,kw OR 'oxaliplatino':ab,ti,kw OR 'oxaliplatinum':ab,ti,kw OR 'oxaliprol':ab,ti,kw OR 'oxaliquid':ab,ti,kw OR 'oxalisan':ab,ti,kw OR 'oxalisin':ab,ti,kw OR 'oxalizor':ab,ti,kw OR 'oxaltic':ab,ti,kw OR 'oxaltina':ab,ti,kw OR 'oxaplamyl':ab,ti,kw OR 'oxaviatin':ab,ti,kw OR 'oyavas':ab,ti,kw OR 'panitumab':ab,ti,kw OR 'panitunumab':ab,ti,kw OR 'pbp1602':ab,ti,kw OR 'pbp1701':ab,ti,kw OR 'pbp2001':ab,ti,kw OR 'pbp2101':ab,ti,kw OR 'pbp2102':ab,ti,kw OR 'pembrolizumab':ab,ti,kw OR 'perjeta':ab,ti,kw OR 'pertuzumab':ab,ti,kw OR 'pf05280014':ab,ti,kw OR 'pf06439535':ab,ti,kw OR 'pf06811462':ab,ti,kw OR 'pf07263896':ab,ti,kw OR 'pf5280014':ab,ti,kw OR 'pf6439535':ab,ti,kw OR 'pf6811462':ab,ti,kw OR 'pf7263896':ab,ti,kw OR 'platox':ab,ti,kw OR 'plaxitin':ab,ti,kw OR 'plx4032':ab,ti,kw OR 'pmc901':ab,ti,kw OR 'pmc902':ab,ti,kw OR 'pobevcy':ab,ti,kw OR 'preveloda':ab,ti,kw OR 'pro169':ab,ti,kw OR 'pusintin':ab,ti,kw OR 'ql1101':ab,ti,kw OR 'ql1207':ab,ti,kw OR 'ql1209':ab,ti,kw OR 'r tpr 023':ab,ti,kw OR 'r05185426':ab,ti,kw OR 'r1273':ab,ti,kw OR 'r340':ab,ti,kw OR 'r435':ab,ti,kw OR 'r597':ab,ti,kw OR 'r7204':ab,ti,kw OR 'ramucirumab':ab,ti,kw OR 'rectoxal':ab,ti,kw OR 'regn3':ab,ti,kw OR 'regorafenib':ab,ti,kw OR 'resihance':ab,ti,kw OR 'rg1273':ab,ti,kw OR 'rg435':ab,ti,kw OR 'rg597':ab,ti,kw OR 'rg7204':ab,ti,kw OR 'rg7446':ab,ti,kw OR 'rhumab 2c4':ab,ti,kw OR 'rhumab-vegf':ab,ti,kw OR 'ribofluor':ab,ti,kw OR 'riboxatin':ab,ti,kw OR 'ridoca':ab,ti,kw OR 'ro0452317':ab,ti,kw OR 'ro091978':ab,ti,kw OR 'ro2 9757':ab,ti,kw OR 'ro4368451':ab,ti,kw OR 'ro4876646':ab,ti,kw OR 'ro5185426':ab,ti,kw OR 'ro5541267':ab,ti,kw OR 'ro7069680':ab,ti,kw OR 'ro7071618':ab,ti,kw OR 'ro7234952':ab,ti,kw OR 'rp46161':ab,ti,kw OR 'rp54780':ab,ti,kw OR 'rph001':ab,ti,kw OR 'rtpr023':ab,ti,kw OR 'samfenet':ab,ti,kw OR 'sb15':ab,ti,kw OR 'sb3':ab,ti,kw OR 'sb8':ab,ti,kw OR 'scb420':ab,ti,kw OR 'scd411':ab,ti,kw OR 'sch052365':ab,ti,kw OR 'sch52365':ab,ti,kw OR 'sch900475':ab,ti,kw OR 'sct501':ab,ti,kw OR 'sct510':ab,ti,kw OR 'si053':ab,ti,kw OR 'sibp01':ab,ti,kw OR 'sinoxal':ab,ti,kw OR 'sn 38':ab,ti,kw OR 'sn3811':ab,ti,kw OR 'snr1611':ab,ti,kw OR 'sok583a1':ab,ti,kw OR 'sr96669':ab,ti,kw OR 'stc103':ab,ti,kw OR 'sti001':ab,ti,kw OR 'stivant':ab,ti,kw OR 'stivarga':ab,ti,kw OR 'strentarga':ab,ti,kw OR 'syd977':ab,ti,kw OR 'syn112':ab,ti,kw OR 'tab008':ab,ti,kw OR 'tab014':ab,ti,kw OR 'tafinlar':ab,ti,kw OR 'tas1-462':ab,ti,kw OR 'tecentriq':ab,ti,kw OR 'tecntriq':ab,ti,kw OR 'temazol':ab,ti,kw OR 'temcad':ab,ti,kw OR 'temodal':ab,ti,kw OR 'temodar':ab,ti,kw OR 'temodex':ab,ti,kw OR 'temodol':ab,ti,kw OR 'temomedac':ab,ti,kw OR 'temostad':ab,ti,kw OR 'temoxol':ab,ti,kw OR 'temozo-cell':ab,ti,kw OR 'temozolomide':ab,ti,kw OR 'tft':ab,ti,kw OR 'thriherpine':ab,ti,kw OR 'tio217':ab,ti,kw OR 'tipiracil':ab,ti,kw OR 'tmt212':ab,ti,kw OR 'tmz bioshuttle':ab,ti,kw OR 'tmza-he':ab,ti,kw OR 'tolak':ab,ti,kw OR 'topotecin':ab,ti,kw OR 'tot102':ab,ti,kw OR 'trametinib':ab,ti,kw OR 'transplastin':ab,ti,kw OR 'trasturel':ab,ti,kw OR 'trastuzumab':ab,ti,kw OR 'trazimera':ab,ti,kw OR 'triflumann':ab,ti,kw OR 'trifluor thymidine':ab,ti,kw OR 'trifluoridine':ab,ti,kw OR 'trifluoro thymidine':ab,ti,kw OR 'trifluorodeoxythymidine':ab,ti,kw OR 'trifluorothymidine':ab,ti,kw OR 'trifluridine':ab,ti,kw OR 'trifuridine':ab,ti,kw OR 'triherpin':ab,ti,kw OR 'triherpine':ab,ti,kw OR 'trs003':ab,ti,kw OR 'tuznue':ab,ti,kw OR 'tx05':ab,ti,kw OR 'tx16':ab,ti,kw OR 'tykerb':ab,ti,kw OR 'tyverb':ab,ti,kw OR 'u101440e':ab,ti,kw OR 'ub921':ab,ti,kw OR 'uflahex':ab,ti,kw OR 'uraciflor':ab,ti,kw OR 'utoral':ab,ti,kw OR 'vascular endothelial growth factor*':ab,ti,kw OR 'vasculotropin trap':ab,ti,kw OR 'vectibex':ab,ti,kw OR 'vectibix':ab,ti,kw OR 'vegf*':ab,ti,kw OR 'vegzelma':ab,ti,kw OR 'velminox':ab,ti,kw OR 'vemurafenib':ab,ti,kw OR 'versavo':ab,ti,kw OR 'viromidin':ab,ti,kw OR 'virophta':ab,ti,kw OR 'viroptic':ab,ti,kw OR 'vivitra':ab,ti,kw OR 'w0090':ab,ti,kw OR 'wetlia':ab,ti,kw OR 'xabine':ab,ti,kw OR 'xaliplat':ab,ti,kw OR 'xalvobin':ab,ti,kw OR 'xdivane':ab,ti,kw OR 'xecap':ab,ti,kw OR 'xelazor':ab,ti,kw OR 'xelcip':ab,ti,kw OR 'xelocel':ab,ti,kw OR 'xeloda':ab,ti,kw OR 'xoplan':ab,ti,kw OR 'xtrudane':ab,ti,kw OR 'yervoy':ab,ti,kw OR 'zaltrap':ab,ti,kw OR 'zedora':ab,ti,kw OR 'zelboraf':ab,ti,kw OR 'zercepac':ab,ti,kw OR 'zerectum':ab,ti,kw OR 'zirabev':ab,ti,kw OR 'ziv aflibercept':ab,ti,kw OR 'zocitab':ab,ti,kw OR 'zrc113':ab,ti,kw OR 'zybev':ab,ti,kw | 809333 |
| #10 | #8 OR #9 | 1360796 |
| #11 | 'random':ab,ti,kw OR 'randomized':ab,ti,kw OR 'trail*':ab,ti,kw OR 'control':ab,ti,kw OR 'controlled':ab,ti,kw | 5754057 |
| #12 | #1 AND #7 AND #10 AND #11 | 12648 |

Cochrane Search Strategy

| Search number | Search | Results |
| --- | --- | --- |
| #1 | ('Metastases' OR 'Metastasis' OR 'metastas*' OR 'Metastatic'):ti,ab,kw | 51411 |
| #2 | [mh "colorectal tumor"] OR [mh "intestine tumor"] OR [mh "cecum tumor"] OR [mh "colon tumor"] OR [mh "rectum tumor"] | 11169 |
| #3 | ('Adenocarcinoma' OR 'bowel tumor' OR 'bowel tumour' OR 'caecal neoplasm' OR 'caecal tumor' OR 'caecal tumour' OR 'caecum tumor' OR 'caecum tumour' OR 'Cecal Cancer' OR 'Cecal Neoplasm*' OR 'cecal tumor' OR 'cecal tumour' OR 'cecum tumor' OR 'cecum tumour' OR 'coecum tumor' OR 'coecum tumour' OR 'Colon Adenocarcinoma*' OR 'Colon Cancer*' OR 'colon mass (tumor)' OR 'colon neoplasia' OR 'Colon Neoplasm*' OR 'colon tumor' OR 'colon tumorigenesis' OR 'colon tumour' OR 'colon villous tumor' OR 'colon villous tumour' OR 'Colonic Cancer*' OR 'colonic mass (tumor)' OR 'colonic masses (tumor)' OR 'colonic neoplasia' OR 'Colonic Neoplasm*' OR 'colonic tumor' OR 'colonic tumorigenesis' OR 'colonic tumour' OR 'Colorectal Cancer*' OR 'Colorectal Carcinoma*' OR 'colorectal neoplasia' OR 'colorectal neoplasm*' OR 'colorectal tumor*' OR 'colorectal tumour' OR 'gut neoplasia' OR 'gut tumor' OR 'gut tumorigenesis' OR 'gut tumour' OR 'intestinal canal tumor' OR 'intestinal canal tumour' OR 'Intestinal Cancer*' OR 'intestinal neoplasia' OR 'Intestinal Neoplasm*' OR 'intestinal tract tumor' OR 'intestinal tract tumour' OR 'intestinal tumor' OR 'intestinal tumorigenesis' OR 'intestinal tumour' OR 'intestine neoplasia' OR 'Intestine Neoplasm*' OR 'intestine tumor' OR 'intestine tumour*' OR 'Intestines Cancer*' OR 'mesocolon tumor' OR 'mesocolon tumour' OR 'neoplasm of the cecum' OR 'neoplasma recti' OR 'neoplasms of the gut' OR 'neoplastic colon' OR 'neoplastic colonic' OR 'neoplastic colorectal' OR 'neoplastic intestinal' OR 'pararectal tumor' OR 'pararectal tumour' OR 'Rectal Cancer*' OR 'rectal mass (tumor)' OR 'rectal neoplasia' OR 'Rectal Neoplasm*' OR 'Rectal Tumor*' OR 'rectal tumour' OR 'Rectum Cancer*' OR 'rectum mass (tumor)' OR 'rectum neoplasia' OR 'Rectum Neoplasm*' OR 'rectum tumor' OR 'rectum tumour' OR 'retrorectal tumor' OR 'retrorectal tumour' OR 'tumor recti' OR 'tumour recti'):ti,ab,kw | 42833 |
| #4 | ('colorectal' OR 'colon' OR 'colonic' OR 'rectum' OR 'rectal' OR 'bowel*' OR 'intestine*' OR 'sigmoid'):ti,ab,kw | 71050 |
| #5 | ('neoplas*' OR 'tumor*' OR 'tumour*' OR 'cancer*' OR 'carcinoma*' OR 'malignan*' OR 'adenocarcinoma*' OR 'adenom*'):ti,ab,kw | 266428 |
| #6 | #4 AND #5 | 34242 |
| #7 | #2 OR #3 OR #6 | 45893 |
| #8 | [mh Adavosertib] OR [mh Aflibercept] OR [mh atezolizumab] OR [mh Bevacizumab] OR [mh Binimetinib] OR [mh Capecitabine] OR [mh Cetuximab] OR [mh Dabrafenib] OR [mh encorafenib] OR [mh Fluorouracil] OR [mh Ipilimumab] OR [mh Irinotecan] OR [mh Lapatinib] OR [mh lenvatinib] OR [mh “monoclonal antibody”] OR [mh Nivolumab] OR [mh Oxaliplatin] OR [mh Panitumumab] OR [mh pembrolizumab] OR [mh pembrolizumab] OR [mh Pertuzumab] OR [mh ramucirumab] OR [mh Regorafenib] OR [mh Temozolomide] OR [mh Tipiracil] OR [mh trametinib] OR [mh Trastuzumab] OR [mh Trifluridine] OR [mh Vemurafenib] | 26458 |
| #9 | (‘1121B' OR '2C4' OR '5HUHexal' OR '5FU' OR 'a168' OR 'abevmy' OR 'abp215' OR 'abp494' OR 'abp938' OR 'abp980' OR 'ABXEGF' OR 'accusite' OR 'ACT078' OR 'actino-hermal' OR 'Adavosertib' OR 'adrucil' OR 'advecit' OR 'aflibercept' OR 'aflomin' OR 'agicil' OR 'ainex' OR 'aiv007' OR 'AL3818' OR 'altl9' OR 'altuzan' OR 'alymsys' OR 'amg954' OR 'amt901' OR 'ankeda' OR 'anlotinib' OR 'anti-PDL1' OR 'apecitab' OR 'arry162' OR 'arry438162' OR 'aryotrust' OR 'askb1202' OR 'atezolizumab' OR 'atubri' OR 'avastin' OR 'ave0005' OR 'ave005' OR 'avegra' OR 'avt06' OR 'axiplatin' OR 'aybintio' OR 'azd1775' OR 'ba1101' OR 'ba1104' OR 'balimek' OR 'bambevi' OR 'bat1706' OR 'bay734506' OR 'bay865321' OR 'bcd021' OR 'bcd022' OR 'bcd201' OR 'bendaplatin' OR 'bephen' OR 'bevacizumab' OR 'bevagen' OR 'bevatas' OR 'bevax' OR 'bevz92' OR 'bi695502' OR 'Binimetinib' OR 'blastomat' OR 'bms734016' OR 'bms936558' OR 'bow030' OR 'boyounuo' OR 'bp01' OR 'bp102' OR 'braftovi' OR 'bryxta' OR 'bs503a' OR 'bx2318' OR 'bxt2316' OR 'byvasda' OR 'c225' OR 'calmtop' OR 'campto' OR 'camptosar' OR 'Camptothecin11' OR 'capcel' OR 'capebina' OR 'capecel' OR 'capecitabine' OR 'capecitalox' OR 'capecite' OR 'capegard' OR 'capezam' OR 'capibine' OR 'capicet' OR 'capiibine' OR 'capnat' OR 'capoda' OR 'capostat' OR 'CAPOX' OR 'capsy' OR 'capxcel' OR 'carac' OR 'caxeta' OR 'cbt124' OR 'ccrg81045' OR 'CCRG-81045' OR 'cdp1' OR 'cerex' OR 'cetuximab' OR 'ch225' OR 'chs2020' OR 'chs305' OR 'chs5217' OR 'cinkefu' OR 'citabin' OR 'cizumab' OR 'clonalantibody' OR 'cmab009' OR 'cmab819' OR 'coloxet' OR 'cpt11' OR 'crisapla' OR 'croloxat' OR 'cs1002' OR 'ctp06' OR 'ctp15' OR 'ctp16' OR 'ctp6' OR 'cyramza' OR 'da3111' OR 'dabrafenib' OR 'dacotin' OR 'dacplat' OR 'debio0507' OR 'dmb3111' OR 'dq2805' OR 'drb436' OR 'e7080' OR 'eb1003' OR 'ebeoxal' OR 'ecansya' OR 'effluderm' OR 'efudex' OR 'efudix' OR 'efurix' OR 'eg12014' OR 'EGFR' OR 'elatofen' OR 'eloxatin' OR 'eloxatine' OR 'elplat' OR 'encorafenib' OR 'epidermalgrowthfactorreceptor' OR 'equidacent' OR 'erbitux' OR 'eurofluor' OR 'eurofluor(drug)' OR 'euroxaliplatin' OR 'eylea' OR 'eylia' OR 'f6627' OR 'first-linetreatment' OR 'fivoflu' OR 'fkb238' OR 'fluorouracil' OR 'fluoroblastin' OR 'fluoroplex' OR 'fluoropyrimidine' OR 'fluorouracil' OR 'fluorouracile' OR 'Fluoro-UracileICN' OR 'Fluorouracil-GRY' OR 'fluorouracilo' OR 'fluoruracil' OR 'fluouracil' OR 'fluoxan' OR 'flurablastin' OR 'fluracedyl' OR 'fluracil' OR 'fluracilium' OR 'fluril' OR 'flurouracil' OR 'fluroblastin' OR 'fluroblastine' OR 'Flurodex' OR 'FOLFIRI' OR 'FOLFOXIRI' OR 'fyb203' OR 'gb222' OR 'gbs004' OR 'gbs012' OR 'geneplatin' OR 'gessedil' OR 'gsk1120212' OR 'gsk2118436' OR 'gw2016' OR 'GW282974X' OR 'gw572016' OR 'HaematoFU' OR 'hanbeitai' OR 'hd201' OR 'hd204' OR 'heloxatin' OR 'Herceptin' OR 'herclon' OR 'hermyl1401o' OR 'herticad' OR 'hertraz' OR 'hervelous' OR 'herzuma' OR 'hlx02' OR 'hlx04' OR 'hlx12' OR 'hlx13' OR 'hot1010' OR 'hs627' OR 'HumanPanitumumabAntibody' OR 'hybridomaantibody' OR 'ibi305' OR 'ibi310' OR 'idb0072' OR 'ifacil' OR 'ihl305' OR 'IMC1121B' OR 'imc225' OR 'imcc225' OR 'immunotherapyortargetedtherapy' OR 'intp24' OR 'Ipilimumab' OR 'ipique' OR 'Irinotecan' OR 'IrinotecanHydrochloride' OR 'irinotel' OR 'Irrinotecan' OR 'jhl1149' OR 'jm83' OR 'js501' OR 'jtp74057' OR 'jy028' OR 'kanjinti' OR 'kapetral' OR 'Keytruda' OR 'kimozo' OR 'kisplyx' OR 'kl140' OR 'krabeva' OR 'kyomarc' OR 'lambrolizumab' OR 'lapatinib' OR 'lapatinibtosylate' OR 'lenvatinib' OR 'lenvima' OR 'lextemy' OR 'LGX818' OR 'liploxa' OR 'lipoxal' OR 'L-OHPCpd' OR 'lumiere(drug)' OR 'ly01008' OR 'ly01012' OR 'ly01015' OR 'ly09004' OR 'ly2939777' OR 'LY3009806' OR 'MandB39831' OR 'MAbC225' OR 'mabionvegf' OR 'mb39831' OR 'mb02' OR 'mb39831' OR 'mbp426' OR 'MDXCTLA4' OR 'MDX010' OR 'mdx101' OR 'MDX1106' OR 'medoxa' OR 'MEK162' OR 'mekinist' OR 'Mektovi' OR 'Methazolastone' OR 'mil60' OR 'MK1775' OR 'mk3475' OR 'mk7365' OR 'mk7902' OR 'MonoclonalAntibod*' OR 'MPDL3280A' OR 'Mvasi' OR 'myl1401o' OR 'myl14020' OR 'myl1402o' OR 'myl1701p' OR 'naprocap' OR 'nc4016' OR 'Neofluor' OR 'Nivolumab' OR 'NK012Compound' OR 'nogron' OR 'nsc18913' OR 'nsc19893' OR 'NSC362856' OR 'nsc704865' OR 'nvplgx818' OR 'ocufridine' OR 'ogivri' OR 'oksaliplatin' OR 'oksaliplatina' OR 'Omnitarg' OR 'onbevzi' OR 'oncofu' OR 'Onkofluor' OR 'ONO4538' OR 'ono7702' OR 'ono7703' OR 'ons1045' OR 'ons1050' OR 'ons5010' OR 'ontruzant' OR 'Opdivo' OR 'oplat' OR 'orp005' OR 'ot702' OR 'oxalatoplatinum' OR 'oxalatplatin' OR 'oxali' OR 'oxalip' OR 'oxaliplan' OR 'Oxaliplatin' OR 'oxaliplatina' OR 'Oxaliplatine' OR 'oxaliplatino' OR 'oxaliplatinum' OR 'oxaliprol' OR 'oxaliquid' OR 'oxalisan' OR 'oxalisin' OR 'oxalizor' OR 'oxaltic' OR 'oxaltina' OR 'oxaplamyl' OR 'oxaviatin' OR 'oyavas' OR 'panitumab' OR 'panitunumab' OR 'pbp1602' OR 'pbp1701' OR 'pbp2001' OR 'pbp2101' OR 'pbp2102' OR 'pembrolizumab' OR 'Perjeta' OR 'Pertuzumab' OR 'pf05280014' OR 'pf06439535' OR 'pf06811462' OR 'pf07263896' OR 'pf5280014' OR 'pf6439535' OR 'pf6811462' OR 'pf7263896' OR 'platox' OR 'plaxitin' OR 'PLX4032' OR 'pmc901' OR 'pmc902' OR 'pobevcy' OR 'preveloda' OR 'pro169' OR 'pusintin' OR 'ql1101' OR 'ql1207' OR 'ql1209' OR 'rtpr023' OR 'R05185426' OR 'r1273' OR 'r340' OR 'r435' OR 'r597' OR 'r7204' OR 'ramucirumab' OR 'rectoxal' OR 'regn3' OR 'regorafenib' OR 'resihance' OR 'rg1273' OR 'rg435' OR 'rg597' OR 'RG7204' OR 'RG7446' OR 'rhumab2C4' OR 'rhuMAb-VEGF' OR 'Ribofluor' OR 'riboxatin' OR 'ridoca' OR 'ro0452317' OR 'ro091978' OR 'ro29757' OR 'ro4368451' OR 'ro4876646' OR 'ro5185426' OR 'ro5541267' OR 'ro7069680' OR 'ro7071618' OR 'ro7234952' OR 'rp46161' OR 'rp54780' OR 'rph001' OR 'rtpr023' OR 'samfenet' OR 'sb15' OR 'sb3' OR 'sb8' OR 'scb420' OR 'scd411' OR 'sch052365' OR 'sch52365' OR 'sch900475' OR 'sct501' OR 'sct510' OR 'si053' OR 'sibp01' OR 'sinoxal' OR 'SN38' OR 'SN3811' OR 'snr1611' OR 'sok583a1' OR 'sr96669' OR 'stc103' OR 'sti001' OR 'stivant' OR 'Stivarga' OR 'strentarga' OR 'syd977' OR 'syn112' OR 'tab008' OR 'tab014' OR 'tafinlar' OR 'Tecentriq' OR 'tecntriq' OR 'temazol' OR 'temcad' OR 'temodal' OR 'Temodar' OR 'temodex' OR 'temodol' OR 'temomedac' OR 'temostad' OR 'temoxol' OR 'temozo-cell' OR 'Temozolomide' OR 'tft' OR 'thriherpine' OR 'tio217' OR 'Tipiracil' OR 'tmt212' OR 'TMZBioshuttle' OR 'TMZA-HE' OR 'tolak' OR 'topotecin' OR 'tot102' OR 'trametinib' OR 'transplastin' OR 'trasturel' OR 'Trastuzumab' OR 'Trazimera' OR 'Triflumann' OR 'trifluorthymidine' OR 'Trifluoridine' OR 'trifluorothymidine' OR 'trifluorodeoxythymidine' OR 'Trifluorothymidine' OR 'Trifluridine' OR 'trifuridine' OR 'triherpin' OR 'triherpine' OR 'trs003' OR 'tuznue' OR 'tx05' OR 'tx16' OR 'Tykerb' OR 'tyverb' OR 'u101440e' OR 'ub921' OR 'uflahex' OR 'uraciflor' OR 'utoral' OR 'vascularendothelialgrowthfactor*' OR 'vasculotropintrap' OR 'vectibex' OR 'Vectibix' OR 'VEGF*' OR 'vegzelma' OR 'velminox' OR 'Vemurafenib' OR 'versavo' OR 'Viromidin' OR 'virophta' OR 'viroptic' OR 'vivitra' OR 'w0090' OR 'wetlia' OR 'xabine' OR 'xaliplat' OR 'xalvobin' OR 'xdivane' OR 'xecap' OR 'xelazor' OR 'xelcip' OR 'xelocel' OR 'Xeloda' OR 'xoplan' OR 'xtrudane' OR 'Yervoy' OR 'Zaltrap' OR 'zedora' OR 'Zelboraf' OR 'zercepac' OR 'zerectum' OR 'zirabev' OR 'zivaflibercept' OR 'zocitab' OR 'zrc113' OR 'zybev'):ti,ab,kw | 53900 |
| #10 | #8 OR #9 | 66512 |
| #11 | ('Random' OR 'Randomized' OR 'trail*' OR 'Control' OR 'Controlled'):ti,ab,kw | 1449455 |
| #12 | #1 AND #7 AND #10 AND #11 | 6005 |

Web of Sciense Search Strategy

| Search number | Query | Results |
| --- | --- | --- |
| #1 | TS=(Metastases OR Metastasis OR metastas* OR Metastatic) | 682864 |
| #2 | (TS=(Metastases OR Metastasis OR metastas* OR Metastatic)) AND TS=(Adenocarcinoma OR bowel tumor OR bowel tumour OR caecal neoplasm OR caecal tumor OR caecal tumour OR caecum tumor OR caecum tumour OR Cecal Cancer OR Cecal Neoplasm* OR cecal tumor OR cecal tumour OR cecum tumor OR cecum tumour OR coecum tumor OR coecum tumour OR Colon Adenocarcinoma* OR Colon Cancer* OR colon mass (tumor) OR colon neoplasia OR Colon Neoplasm* OR colon tumor OR colon tumorigenesis OR colon tumour OR colon villous tumor OR colon villous tumour OR Colonic Cancer* OR colonic mass (tumor) OR colonic masses (tumor) OR colonic neoplasia OR Colonic Neoplasm* OR colonic tumor OR colonic tumorigenesis OR colonic tumour OR Colorectal Cancer* OR Colorectal Carcinoma* OR colorectal neoplasia OR colorectal neoplasm* OR colorectal tumor* OR colorectal tumour OR gut neoplasia OR gut tumor OR gut tumorigenesis OR gut tumour OR intestinal canal tumor OR intestinal canal tumour OR Intestinal Cancer* OR intestinal neoplasia OR Intestinal Neoplasm* OR intestinal tract tumor OR intestinal tract tumour OR intestinal tumor OR intestinal tumorigenesis OR intestinal tumour OR intestine neoplasia OR Intestine Neoplasm* OR intestine tumor OR intestine tumour* OR Intestines Cancer* OR mesocolon tumor OR mesocolon tumour OR neoplasm of the cecum OR neoplasma recti OR neoplasms of the gut OR neoplastic colon OR neoplastic colonic OR neoplastic colorectal OR neoplastic intestinal OR pararectal tumor OR pararectal tumour OR Rectal Cancer* OR rectal mass (tumor) OR rectal neoplasia OR Rectal Neoplasm* OR Rectal Tumor* OR rectal tumour OR Rectum Cancer* OR rectum mass (tumor) OR rectum neoplasia OR Rectum Neoplasm* OR rectum tumor OR rectum tumour OR retrorectal tumor OR retrorectal tumour OR tumor recti OR tumour recti) | 147166 |
| #3 | TS=(colorectal OR colon OR colonic OR rectum OR rectal OR bowel* OR intestine* OR sigmoid) | 896650 |
| #4 | TS=(colorectal OR colon OR colonic OR rectum OR rectal OR bowel* OR intestine* OR sigmoid) | 896650 |
| #5 | #3 AND #4 | 896650 |
| #6 | #2 OR #5 | 945544 |
| #7 | TS=(1121B OR 2C4 OR 5 HU Hexal OR 5FU OR a168 OR abevmy OR abp215 OR abp494 OR abp938 OR abp980 OR ABX EGF OR accusite OR ACT078 OR actino-hermal OR Adavosertib OR adrucil OR advecit OR aflibercept OR aflomin OR agicil OR ainex OR aiv007 OR AL3818 OR altl9 OR altuzan OR alymsys OR amg954 OR amt901 OR ankeda OR anlotinib OR anti-PDL1 OR apecitab OR arry162 OR arry438162 OR aryotrust OR askb1202 OR atezolizumab OR atubri OR avastin OR ave0005 OR ave005 OR avegra OR avt06 OR axiplatin OR aybintio OR azd1775 OR ba1101 OR ba1104 OR balimek OR bambevi OR bat1706 OR bay734506 OR bay865321 OR bcd021 OR bcd022 OR bcd201 OR bendaplatin OR bephen OR bevacizumab OR bevagen OR bevatas OR bevax OR bevz92 OR bi695502 OR Binimetinib OR blastomat OR bms734016 OR bms936558 OR bow030 OR boyounuo OR bp01 OR bp102 OR braftovi OR bryxta OR bs503a OR bx2318 OR bxt2316 OR byvasda OR c225 OR calmtop OR campto OR camptosar OR Camptothecin 11 OR capcel OR capebina OR capecel OR capecitabine OR capecitalox OR capecite OR capegard OR capezam OR capibine OR capicet OR capiibine OR capnat OR capoda OR capostat OR CAPOX OR capsy OR capxcel OR carac OR caxeta OR cbt124 OR ccrg81045 OR CCRG-81045 OR cdp1 OR cerex OR cetuximab OR ch225 OR chs2020 OR chs305 OR chs5217 OR cinkef u OR citabin OR cizumab OR clonal antibody OR cmab009 OR cmab819 OR coloxet OR cpt11 OR crisapla OR croloxat OR cs1002 OR ctp06 OR ctp15 OR ctp16 OR ctp6 OR cyramza OR da3111 OR dabrafenib OR dacotin OR dacplat OR debio0507 OR dmb3111 OR dq2805 OR drb436 OR e7080 OR eb1003 OR ebeoxal OR ecansya OR effluderm OR efudex OR efudix OR efurix OR eg12014 OR EGFR OR elatofen OR eloxatin OR eloxatine OR elplat OR encorafenib OR epidermal growthfactor receptor OR equidacent OR er203492-00 OR erbitux OR eurofluor OR eurofluor (drug) OR euroxaliplatin OR eylea OR eylia OR f6627 OR first-line treatment OR fivoflu OR fkb238 OR fluoro uracil OR fluoroblastin OR fluoroplex OR fluoropyrimidine OR fluorouracil OR fluorouracile OR Fluoro-Uracile ICN OR Fluorouracil-GRY OR fluorouracilo OR fluoruracil OR fluouracil OR fluoxan OR flurablastin OR fluracedyl OR fluracil OR fluracilium OR fluril OR fluro uracil OR fluroblastin OR fluroblastine OR Flurodex OR FOLFIRI OR FOLFOXIRI OR fyb203 OR gb222 OR gbs004 OR gbs012 OR geneplatin OR gessedil OR gsk1120212 OR gsk2118436 OR gw2016 OR GW282974X OR gw572016 OR Haemato FU OR hanbeitai OR hd201 OR hd204 OR heloxatin OR Herceptin OR herclon OR hermyl1401o OR herticad OR hertraz OR hervelous OR herzuma OR hlx02 OR hlx04 OR hlx12 OR hlx13 OR hot1010 OR hs627 OR Human Panitumumab Antibody OR hybridoma antibody OR ibi305 OR ibi310 OR idb0072 OR ifacil OR ihl305 OR IMC1121B OR imc225 OR imcc225 OR immunotherapy or targeted therapy OR intp24 OR Ipilimumab OR ipique OR Irinotecan OR Irinotecan Hydrochloride OR irinotel OR Irrinotecan OR jhl1149 OR jm83 OR js501 OR jtp74057 OR jy028 OR kanjinti OR kapetral OR Keytruda OR kimozo OR kisplyx OR kl140 OR krabeva OR kyomarc OR lambrolizumab OR lapatinib OR lapatinib tosylate OR lenvatinib OR lenvima OR Leucovorin or 5-FU OR lextemy OR LGX818 OR liploxa OR lipoxal OR L-OHP Cpd OR lumiere (drug) OR ly01008 OR ly01012 OR ly01015 OR ly09004 OR ly2939777 OR LY3009806 OR M and B39831 OR MAb C225 OR mabionvegf OR mb 39831 OR mb02 OR mb39831 OR mbp426 OR MDX CTLA 4 OR MDX010 OR mdx101 OR MDX1106 OR medoxa OR MEK162 OR mekinist OR Mektovi OR Methazolastone OR mil60 OR MK1775 OR mk3475 OR mk7365 OR mk7902 OR Monoclonal Antibod* OR MPDL3280A OR Mvasi OR myl1401o OR myl14020 OR myl1402o OR myl1701p OR naprocap OR nc4016 OR Neofluor OR Nivolumab OR NK012 Compound OR nogron OR nsc18913 OR nsc19893 OR NSC362856 OR nsc704865 OR nvp lgx818 OR ocufridine OR ogivri OR oksaliplatin OR oksaliplatina OR Omnitarg OR onbevzi OR oncofu OR Onkofluor OR ONO4538 OR ono7702 OR ono7703 OR ons1045 OR ons1050 OR ons5010 OR ontruzant OR Opdivo OR oplat OR orp005 OR ot702 OR oxalatoplatinum OR oxalatplatin OR oxali OR oxalip OR oxaliplan OR Oxaliplatin OR oxaliplatina OR Oxaliplatine OR oxaliplatino OR oxaliplatinum OR oxaliprol OR oxaliquid OR oxalisan OR oxalisin OR oxalizor OR oxaltic OR oxaltina OR oxaplamyl OR oxaviatin OR oyavas OR panitumab OR panitunumab OR pbp1602 OR pbp1701 OR pbp2001 OR pbp2101 OR pbp2102 OR pembrolizumab OR Perjeta OR Pertuzumab OR pf05280014 OR pf06439535 OR pf06811462 OR pf07263896 OR pf5280014 OR pf6439535 OR pf6811462 OR pf7263896 OR platox OR plaxitin OR PLX4032 OR pmc901 OR pmc902 OR pobevcy OR preveloda OR pro169 OR pusintin OR ql1101 OR ql1207 OR ql1209 OR r tpr 023 OR R05185426 OR r1273 OR r340 OR r435 OR r597 OR r7204 OR ramucirumab OR rectoxal OR regn3 OR regorafenib OR resihance OR rg1273 OR rg435 OR rg597 OR RG7204 OR RG7446 OR rhumab 2C4 OR rhuMAb-VEGF OR Ribofluor OR riboxatin OR ridoca OR ro0452317 OR ro091978 OR ro2 9757 OR ro4368451 OR ro4876646 OR ro5185426 OR ro5541267 OR ro7069680 OR ro7071618 OR ro7234952 OR rp46161 OR rp54780 OR rph001 OR rtpr023 OR samfenet OR sb15 OR sb3 OR sb8 OR scb420 OR scd411 OR sch052365 OR sch52365 OR sch900475 OR sct501 OR sct510 OR si053 OR sibp01 OR sinoxal OR SN 38 OR SN3811 OR snr1611 OR sok583a1 OR sr96669 OR stc103 OR sti001 OR stivant OR Stivarga OR strentarga OR syd977 OR syn112 OR tab008 OR tab014 OR tafinlar OR tas1-462 OR Tecentriq OR tecntriq OR temazol OR temcad OR temodal OR Temodar OR temodex OR temodol OR temomedac OR temostad OR temoxol OR temozo-cell OR Temozolomide OR tft OR thriherpine OR tio217 OR Tipiracil OR tmt212 OR TMZ Bioshuttle OR TMZA-HE OR tolak OR topotecin OR tot102 OR trametinib OR transplastin OR trasturel OR Trastuzumab OR Trazimera OR Triflumann OR trifluor thymidine OR Trifluoridine OR trifluoro thymidine OR trifluorodeoxythymidine OR Trifluorothymidine OR Trifluridine OR trifuridine OR triherpin OR triherpine OR trs003 OR tuznue OR tx05 OR tx16 OR Tykerb OR tyverb OR u101440e OR ub921 OR uflahex OR uraciflor OR utoral OR vascular endothelial growth factor* OR vasculotropin trap OR vectibex OR Vectibix OR VEGF* OR vegzelma OR velminox OR Vemurafenib OR versavo OR Viromidin OR virophta OR viroptic OR vivitra OR w0090 OR wetlia OR xabine OR xaliplat OR xalvobin OR xdivane OR xecap OR xelazor OR xelcip OR xelocel OR Xeloda OR xoplan OR xtrudane OR Yervoy OR Zaltrap OR zedora OR Zelboraf OR zercepac OR zerectum OR zirabev OR ziv aflibercept OR zocitab OR zrc113 OR zybev) | 1302516 |
| #8 | TS=(Random OR Randomized OR trail* OR Control OR Controlled) | 8841429 |
| #9 | #1 AND #6 AND #7 AND #8 | 12488 |
